# Effects of liberal versus restrictive transfusion strategies on intermittent hypoxaemia in extremely low birthweight infants: secondary analyses of the ETTNO randomised controlled trial

**DOI:** 10.1136/archdischild-2024-327643

**Published:** 2025-03-26

**Authors:** Axel R Franz, Corinna Engel, Dirk Bassler, Mario Rüdiger, Ulrich H Thome, Rolf F Maier, Ingeborg Krägeloh-Mann, Jochen Essers, Christoph Bührer, Hans-Jörg Bittrich, Claudia Roll, Thomas Höhn, Harald Ehrhardt, Ralf Boettger, Hans Thorsten Körner, Anja Stein, Patrick Neuberger, Tine Brink Henriksen, Gorm Greisen, Christian F Poets, for the ETTNO Investigators, Christian A Maiwald

**Affiliations:** 1 Center for Pediatric Clinical Studies, University of Tübingen, Tubingen, Germany; 2 Neonatology, University Children’s Hospital Tübingen, Tübingen, Germany; 3 Neonatology, UniversitatsSpital Zurich Klinik fur Neonatologie, Zurich, Switzerland; 4 Department for Neonatology and Paediatric Intensive Care Medicine, Medizinische Fakultät, Technische Universitat Dresden, Dresden, Germany; 5 Neonatology, University of Leipzig, Leipzig, Germany; 6 Faculty of Medicine, Children’s Hospital, Philipps University Marburg, Marburg, Germany; 7 University Children’s Hospital Tuebingen, Tuebingen, Germany; 8 Neonatology and Pediatric Intensive Care Medicine, Ulm University Medical Center, Ulm, Germany; 9 Neonatology, Charité Universitätsmedizin Berlin, Berlin, Germany; 10 Neonatology, Helios Klinik Erfurt, Erfurt, Germany; 11 Neonatology and Paediatric Intensive Care, Vest Childern’s Hospital Datteln, University Witten-Herdecke, Datteln, Germany; 12 University Hospital Düsseldorf Neonatology and Pediatric Intensive Care, Düsseldorf, Germany; 13 General Pediatrics & Neonatology, Justus Liebig University Giessen Faculty of Medicine, Giessen, Germany; 14 Universitatsklinikum Magdeburg, Magdeburg, Germany; 15 Neonatology, Klinikum Bremen-Mitte gGmbH, Bremen, Germany; 16 Pediatrics-Neonatology, University Hospital Essen, Essen, Germany; 17 Neonatology, Olgahospital Stuttgart, Stuttgart, Germany; 18 Child and Adolescent Health, Aarhus University Hospital, Aarhus N, Denmark; 19 Neonatology, Rigshospitalet, Copenhagen, Denmark; 20 Neonatology, University of Tübingen, Tubingen, Germany

**Keywords:** Paediatrics, Infant Development

## Abstract

**Objectives:**

To compare the effect of liberal versus restrictive transfusion strategies on the proportion of time (%time) spent with intermittent hypoxaemia (IH, ie, arterial haemoglobin oxygen saturation measured by pulse oximetry (SpO_2_) <80% lasting ≥60 s) in the ‘Effects of Transfusion Thresholds on Neurocognitive Outcome’ (ETTNO) population, and to investigate whether infants with above-median exposure to IH might benefit more from liberal transfusion strategies than those with lower exposure.

**Design, setting, patients:**

Secondary analysis in all 554/1013 infants of <1000 g birth weight recruited into the ETTNO trial (mean gestational age 26.2 weeks) with >80% completeness of SpO_2_ recordings during postnatal days 8–49.

**Intervention:**

Randomly assigned liberal (n=268) or restrictive (n=286) transfusion strategies, defining transfusion triggers based on postnatal age and health status.

**Main outcome measures:**

%time with IH, rate and mean duration of IH episodes during postnatal days 8–49. Interaction between exposure to IH and transfusion strategies with respect to ETTNO’s composite primary outcome, death or disability at 24 months corrected age.

**Results:**

The median (quartile 1–quartile 3) %time with IH was similar between treatment groups (0.91% (0.13%–2.83%) with liberal vs 0.79% (0.16%–2.44%) with restrictive transfusions). There was no interaction between exposure to IH and transfusion strategies on outcome at 24 months.

**Conclusions:**

In infants <1000 g birth weight, a liberal transfusion strategy did not reduce IH. Blood transfusions should not be administered ‘liberally’ to reduce IH or to improve neurocognitive outcome in infants with above-average exposure to IH.

**Trial registration number:**

NCT01393496.

**WHAT IS ALREADY KNOWN ON THIS TOPIC**

- In extremely preterm infants, liberal versus restrictive transfusion strategies do not improve short-term and neurocognitive outcomes. Intermittent hypoxaemia is associated with adverse outcomes, and short-term observations suggest that transfusion may improve intermittent hypoxaemia.

**WHAT THIS STUDY ADDS**

- The liberal transfusion strategy did not reduce the burden of intermittent hypoxaemia during postnatal days 8–49. There was no interaction between burden of intermittent hypoxaemia and randomly assigned transfusion strategies with respect to outcome at 24 months corrected age.

**HOW THIS STUDY MIGHT AFFECT RESEARCH, PRACTICE OR POLICY**

- Liberal transfusions did not improve intermittent hypoxaemia during postnatal days 8–49. There was no benefit from liberal transfusions with respect to outcome at 24 months in the subgroup of infants with exposure to intermittent hypoxaemia above the median.

## Introduction

 Extremely low birthweight (ELBW) infants, that is, those with birth weight <1000 g, uniformly develop ‘anaemia of prematurity’, caused by developmentally regulated ‘physiological’ and non-physiological, iatrogenic and morbidity-related factors, reviewed by Widness.^1^ About 50%–80% of ELBW infants studied in 2006–2007 received one or more red blood cell transfusions (RBCTs) during their initial hospitalisation.^2 3^

Additionally, these infants experience intermittent hypoxaemia (IH), predominantly caused by recurrent apnoea due to immature respiratory neuronal network (reviewed by Di Fiore *et al* and Martin *et al*^4 5^), but also secondary to active exhalation^6^ or squirming. Short-term, pretransfusion/post-transfusion observational studies indicated that the frequency and depth of IH may acutely respond to RBCT.^7^,^9^

The combination of anaemia and IH may impair oxygen supply to the brain and hence contribute to prematurity-related cognitive and motor deficits, while IH might be better tolerated at higher haemoglobin concentrations preserving arterial blood oxygen content and oxygen delivery during IH.

However, two recent randomised controlled trials on liberal versus restrictive transfusion strategies in ELBW infants, together enrolling >2800 infants, did not show any benefit of more liberal transfusions on neurocognitive development at 24 months corrected age or on important short-term outcomes such as rates of common complications of prematurity.^10 11^ However, these trials did not specifically address the role of transfusions to prevent insufficient oxygen supply to the brain due to combined anaemia and IH.

Consequently, this secondary analysis of the ‘Effects of Transfusion Thresholds on Neurocognitive Outcome in ELBW infants’ (ETTNO) randomised controlled trial^10^ aimed to compare the effects of liberal versus restrictive transfusion strategies on the proportion of time (%time) with IH and to verify interactions between IH and transfusion strategies with respect to outcome.

## Methods

### Trial design and oversight

The underlying multicentre, open, randomised, parallel-group comparison of liberal versus restrictive RBCT strategies was conducted in 36 European neonatal intensive care units. The study protocol^12^ (also provided as online online supplemental file 2) and main results^10^ have been published.

### Patients

The only inclusion criterion for ETTNO was a birth weight of 400–999 g.

Exclusion criteria were gestational age (GA) at birth >29+6/7 weeks and major anomalies. For multiple pregnancies, only the eligible sibling with the lowest birth order was enrolled.

For this secondary analysis, only infants with continuous recordings of arterial haemoglobin oxygen saturation determined by pulse oximetry (SpO_2_) for >80% of the time during postnatal days 8–49 were included by a priori decision (to avoid bias from non-representative SpO_2_ data). Incomplete SpO_2_ recordings were caused by early transfer, technical issues and non-availability of staff to read-out oximeters. Thirty-two of 36 centres contributed data.

### Trial procedures

As previously described,^10^ infants were randomly assigned at a mean (SD) age of 2.5 (0.7) days to one of two treatment groups. The RBCT haematocrit trigger thresholds prescribed from randomisation to discharge home (or transfer) depended on postnatal age and current state of health (‘critical’ vs ‘non-critical’). In the ETTNO trial, a ‘critical’ state of health indicating higher transfusion triggers in both groups was defined (among other criteria) by ‘>6 nurse-documented apnoeas requiring intervention or >4 hypoxaemic episodes to SpO_2_ <60% per 24 hours’.^10^ If indicated, a dose of 20 mL/kg of standard leucocyte-depleted erythrocyte concentrate was administered. Participating centres chose SpO_2_ target ranges, which were classified as ‘lower’ if the centre value was <91% and ‘higher’ if ≥91%.^10^

### SpO_2_ data recording and processing

At all participating centres, SpO_2_ was recorded at 2–4 s averaging and a sampling rate of 1 every 6 s using SET (Masimo Inc, Irvine, California) implemented in Radical 7C pulse oximeters (software versions 7619, 7620, 7720 and 7740) and read-out every 5–7 days.

Downloaded SpO_2_ data were screened for validity; invalid measurements (ie, duplicate recordings, recordings with SpO_2_=0%, and those flagged by the monitor for displaced sensor, ambient light, interference, or low perfusion) were discarded.

### Definition of analysis intervals

Data were extracted for postnatal days 8–49 (grouped into three intervals: postnatal days 8–21, 22–35 and 36–49), because IH largely occurs beyond day 7 and has its maximum at 4–5 weeks.^13^,^15^

### Definition of IH events

IH events considered for analysis were those with arterial haemoglobin oxygen saturation (SpO_2_) <80% lasting ≥60 s (≥10 consecutive data points), because these had previously been found associated with adverse outcomes.^15^ Durations of eligible IH events were summed up and divided by the length of the respective observational interval to obtain %time with IH.

### Outcomes

First, the effect of the randomly assigned transfusion thresholds on %time with IH as well as number and mean duration of IH episodes were evaluated.

Second, interactions between exposure to IH and assigned transfusion thresholds with respect to the long-term composite primary outcome of the ETTNO trial, that is, death or neurodevelopmental impairment (NDI) at 24 (±1) months corrected age, were evaluated. NDI was defined as any of the following: cognitive deficit (defined as Mental Developmental Index (MDI) score on the Bayley Scales of Infant Development (second edition, <85 or similar), cerebral palsy (defined according to the network 'Surveillance of CP in Europe'^16 17^) or severe visual or hearing impairment.

Additional predefined outcome variables were cognitive deficit and cerebral palsy as components of the above composite endpoint. Subgroups with above/below median exposure to IH were evaluated for effects of randomly assigned transfusion thresholds on these outcomes.

### Statistical analysis

All analyses were secondary and hence exploratory.

Burden of IH (cumulative %time, number and mean duration of episodes with SpO_2_<80% (and, post hoc, SpO_2_<60%) lasting for ≥60 s) was compared between treatment groups using the Wilcoxon test.

Univariate and multiple logistic regression using factors, such as treatment group, GA at birth (<26 versus ≥26 weeks), sex, small for gestational age (birth weight SD score <−1.28 vs ≥−1.28 (ie, </≥ 10^th^ percentile)), any antenatal corticosteroids (yes/no) and multiple birth (yes/no) were calculated to identify influencing factors for all outcome variables using backward selection and keeping the factor ‘%time with SpO_2_<80% (episodes ≥60 s) above/below the median’ in the model. All influencing factors were evaluated for potential collinearities and for interactions with IH if appropriate.

Statistical analyses were performed using SAS V.9.4.

## Results

**Figure 1:**
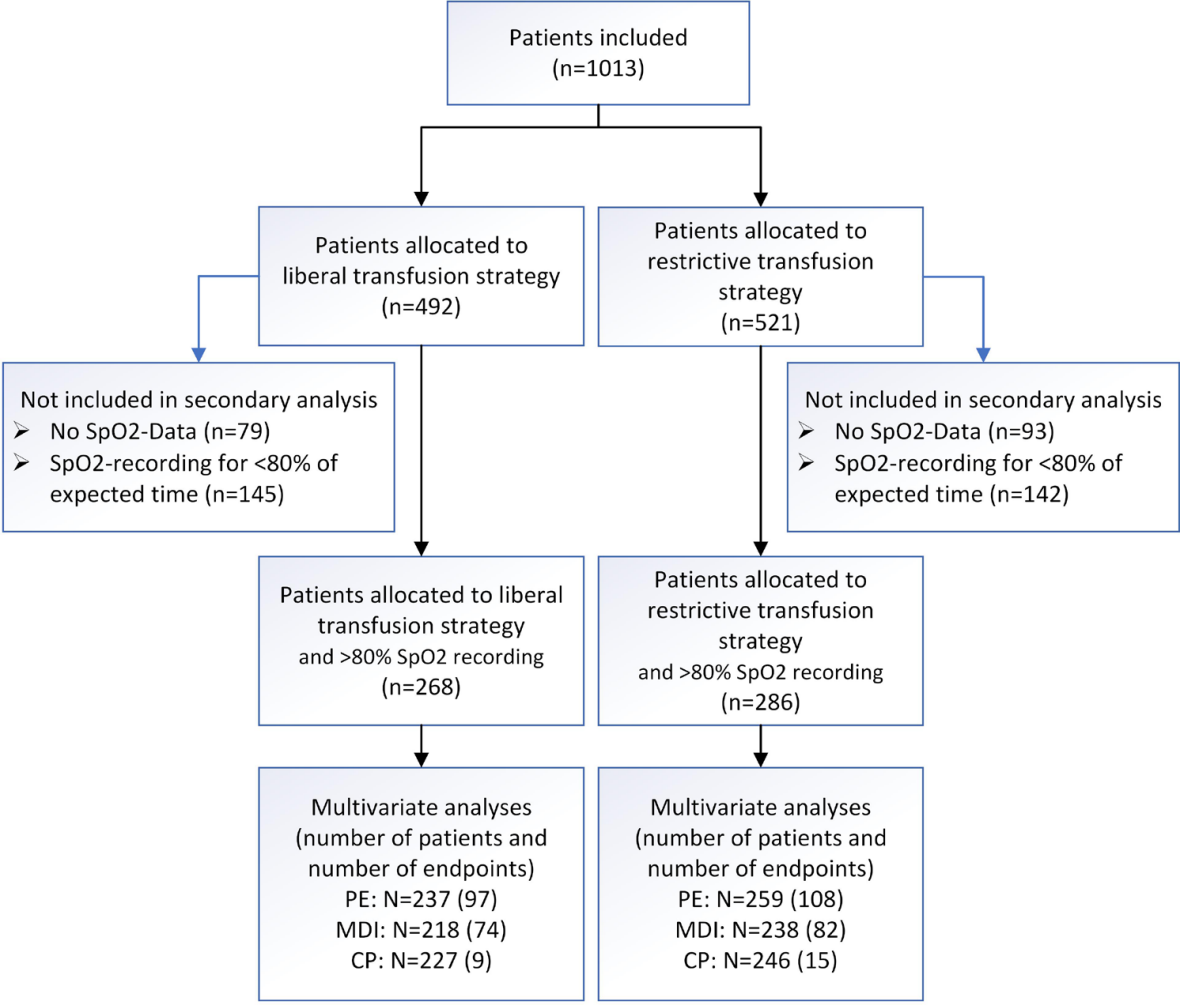
Flow diagram. Number of infants included in the analysis of effects of transfusion thresholds on intermittent hypoxaemia and in multivariate analyses are indicated by treatment group; number of infants with respective endpoint is in parenthesis. PE refers to primary endpoint of the underlying ETTNO trial (ie, death or neurodevelopmental impairment at 24 months corrected age); MDI refers to cognitive deficit indicated by a Bayley second edition Mental Developmental Index score <85 at 24 months corrected age; CP refers to cerebral palsy at 24 months corrected age. CP, cerebral palsy; ETTNO, Effects of Transfusion Thresholds on Neurocognitive Outcome; MDI, Mental Developmental Index; PE, primary endpoint; SpO_2_, oxygen saturation.

Between 14 July 2011 and 14 November 2014, 1013 infants were enrolled (492 assigned to liberal and 521 to restrictive thresholds). Of these, 286 infants from the restrictive and 268 from the liberal group had >80% of continuous SpO_2_ recordings during postnatal days 8–49 (figure 1). In 172 patients, SpO_2_ recordings were not attempted (insufficient staff for downloads), and in 287, SpO_2_ recordings amounted to≤80%.

There were no between-treatment group differences regarding baseline characteristics for infants contributing SpO_2_ data and no differences between those included or excluded from this secondary analysis (table 1).

**Table 1:** Patient characteristics

Patient characteristics	Included with >80% of time with continuous SpO_2_ data		Excluded for ≤80% of time with continuous SpO_2_ data		All
	Liberal (n=268)	Restrictive (n=286)	Liberal (n=224)	Restrictive (n=235)	(n=1013)
Gestational age at birth (weeks)					
Median (Q1–Q3)	26.1 (25.0–27.4)	26.3 (25.0–27.6)	26.0 (24.9–27.4)	26.6 (24.9–27.7)	26.2 (24.9–27.6)
Gestational age at birth <26 weeks					
n (%)	117 (43.7)	122 (42.7)	108 (48.2)	93 (39.6)	440 (43.4)
Birth weight (g)					
Median (Q1–Q3)	740 (630–895)	745 (630–880)	758 (640–902)	780 (630–900)	750 (630–890)
Birth weight stratum <750 g					
n (%)	141 (52.6)	146 (51.1)	109 (48.7)	109 (46.4)	505 (49.9)
Birth weight SDS <−1.68					
n (%)	33 (12.3)	32 (11.2)	22 (9.8)	27 (11.5)	114 (11.3)
Female					
n (%)	146 (54.5)	140 (49.0)	100 (44.6)	123 (52.3)	509 (50.3)
Singleton					
n (%)	222 (82.8)	221 (77.3)	183 (61.7)	186 (79.2)	812 (80.2)
Any antenatal corticosteroids					
n (%)	227 (84.7)	245 (85.7)	206 (91.5)	206 (87.7)	884 (87.3)

Q1–Q3, Quartile 1–Quartile 3.

SDS, standard deviation score; SpO_2_, oxygen saturation.

The distribution of measured SpO_2_ values and haemoglobin concentrations according to transfusion strategy and postnatal age are depicted in online supplemental eFigure 1.

The burden of IH, evaluated as cumulative %time, number and mean duration of IH episodes, was similar for infants in the liberal and in the restrictive transfusion strategy group (table 2 and online supplemental eTable 1). The highest burden of IH occurred during postnatal days 8–35 and in infants with lower gestational age (online supplemental eFigure 2).

**Table 2:** Effects of transfusion strategy on the burden of intermittent hypoxaemia

Burden of intermittent hypoxaemia	Contributing >80% of time with continuous SpO_2_ data		P value
	Liberal (n=268)	Restrictive (n=286)	
Proportion of time contributing SpO_2_(%)			
Median (P10–P90)	93 (83–99)	92 (83–99)	
Proportion of time with SpO_2_ <80% (%) only episodes ≥60 s			
Median (Q1–Q3)	0.91 (0.13–2.83)	0.79 (0.16–2.44)	0.75
No. of events with SpO_2_ <80% (n) only episodes ≥60 s			
Median (Q1–Q3)	236 (36–602)	203 (45–551)	0.73
Mean duration of events* with SpO_2_ <80% (s) only episodes ≥60 s			
Median (Q1–Q3) (no. of patients analysed)†	122 (106–369) (n=257)	122 (104–532) (n=280)	0.66
Proportion of time with SpO_2_ <60% (%) only episodes ≥60 s			
Median (Q1–Q3)	0.02 (0.00–0.09)	0.02 (0.00–0.10)	0.60
No. of events with SpO_2_<60% (n) only episodes ≥60 s			
Median (Q1–Q3)	4 (0–28)	6 (0–29)	0.58
Mean duration of events* with SpO_2_ <60% (s) only episodes ≥60 s			
Median (Q1–Q3) (no. of patients analysed)†	100 (86–116) (n=190)	101 (86–116) (n=209)	0.76

Data are depicted as median (quartile 1–quartile 3).

Note: 1% time is ~600 min (or ~10 hours) in the 6 weeks observational period (6 weeks=60 480 min or 1008 hours).

The respective data for infants contributing continuous SpO_2_ data for ≤80% of time are provided in online supplemental eTable 1.

* The mean duration of events was only calculated in patients with at least one event >60 s.

† No. of patients analysed is provided if deviating from the overall number of patients included (excluding patients without event).

SpO_2_, oxygen saturation.

In the subgroup of infants with above-median exposure to IH, the rates of death or NDI were 66 (54%)/122 in the liberal versus 63 (50%)/126 in the restrictive group (table 3).

**Table 3:** Primary and key secondary outcomes of ETTNO by exposure to intermittent hypoxaemia and transfusion thresholds

	%time with IH ≤median		%time with IH >median	
	Liberal	Restrictive	Liberal	Restrictive
Primary outcome				
n/N (%)	31/115 (27.0%)	45/133 (33.8%)	66/122 (54.1%)	63/126 (50.0%)
OR (95% CI) p value*	0.72 (0.42–1.25) p=0.24		1.18 (0.72–1.94) p=0.52	
Cerebral palsy				
n/N (%)	5/110 (4.5%)	5/126 (4.0%)	4/117 (3.4%)	10/120 (8.3%)
OR (95% CI) p value*	1.15 (0.32–4.09) p=0.83		0.39 (0.12–1.28) p=0.12	
MDI <85†				
n/N (%)	23/106 (21.6%)	35/122 (28.7%)	51/112 (45.5%)	47/116 (40.5%)
OR (95% CI) p value*	0.69 (0.38–1.26) p=0.23		1.23 (0.73–2.08) p=0.44	

Subgroups of exposure to IH as defined as above/below median for %time with SpO_2_ <80% for events ≥60 s. There was no significant interaction of exposure to IH and treatment, that is, no difference in treatment effect between subgroups, as assessed by multiple logistic regression (p values for interaction ‘IH * transfusion threshold group’ for models with factors treatment group and exposure to IH (above/below median) were 0.19, 0.22 and 0.16 for the primary endpoint, cerebral palsy and MDI <85, respectively).

* Logistic regression with factor treatment group.

† MDI Score of the Bayley Scales of Infant and Toddler Development (2nd edition).

ETTNO, Effects of Transfusion Thresholds on Neurocognitive Outcome; MDI, Mental Developmental Index.

When controlling for potentially influencing factors selected from gestational age, sex, small for gestational age, antenatal corticosteroids, multiple birth and transfusion strategy in multiple logistic regression, no interaction between exposure to IH and treatment group related to outcome was detected. Details on the ‘best’ models are provided in online supplemental eTable 2.

## Discussion

This secondary analysis of the ETTNO trial compared effects of liberal versus restrictive transfusion strategies on burden of hypoxaemia (%time with SpO_2_ <80%/ArtO_2_Hb <6.4 g/dL for episodes lasting ≥60 s) and evaluated the data for an interaction between burden of hypoxaemia and transfusion thresholds related to predefined outcomes.

The randomly assigned transfusion strategies applied in ETTNO had no impact on the %time with SpO_2_ <80% during postnatal days 8–49. Considering previous reports on a reduction of IH in the first 1–3 days after transfusion,^7^,^9^ our data suggest that such benefit must be transient and that liberal transfusions, resulting in an overall mean haemoglobin increase of ~1 g/dL^10^ will not reduce IH during postnatal days 8–49. Hence, liberal transfusion strategies as in ETTNO should not be adopted to reduce IH (even though a RBCT in an anaemic infant with severe IH may acutely/transiently reduce the burden of IH).

Evaluating how the burden of hypoxaemia and transfusion thresholds might interact related to predefined long-term outcomes in the ETTNO cohort, we found no statistically significant interaction in multiple logistic regression models and no benefit from liberal transfusions in the subgroup of infants with above-median exposure to IH.

Why did the liberal transfusion strategy, which effectively improved haematocrit and hence oxygen-carrying capacity, not reduce rates of adverse outcomes?^10 11^ Potential explanations include:

1. Previously reported associations between IH and adverse outcomes are not causal, and IH may be a consequence of factors (particularly low gestational age) leading to an adverse outcome rather than the cause of adverse outcome, that is, IH is an indicator of brain immaturity/vulnerability.
2. Liberal transfusions, although improving concentrations of haemoglobin, do not improve oxygen delivery to the brain because physiological compensatory mechanisms (increased cardiac output^18^ or oxygen extraction,^19^ cerebral vasodilation, etc) sufficiently protect the brain from hypoxic insult, at least within the transfusion policies applied in ETTNO.
3. Liberal transfusions, although improving oxygen transport to the brain, do not improve outcome because RBCT have yet undetermined adverse effects (eg, increased hyperoxic injury, increased load of oxygen radicals, etc) that offset any benefit.

Explanation (1) above (‘no causal relation’) is supported by the finding that the main predictor for adverse outcome was low gestational age and this was also strongly associated with increased hypoxaemic burden (eg, because of more severe lung disease and more immature respiratory control). To illustrate these considerations, a simplified hypothetical model of prematurity-related hypoxic brain injury and the potential impacts of blood transfusions is provided in online supplemental eFigure 3.

### Strengths and limitations

Strengths of our study are that data were from a rigorously performed large multicentre RCT, relied on a large patient cohort and had predefined and monitored outcome variables.

Because of missing continuous documentation of FiO_2_, our data did not permit to differentiate SpO_2_ >97% attributable to healthy lungs and normal gas exchange (likely harmless) and hyperoxia/hyperoxaemia due to inadequately high FiO_2_. Consequently, %time in SpO_2_ target and %time with hyperoxaemia could not be calculated. Compared with Canadian Oxygen Trial (COT),^15^ SpO_2_ was less rigorously recorded (not part of the primary intervention) and due to early transfers, recordings that lasted beyond postnatal day 49 were limited. Overall, only 55% of the primary ETTNO cohort was included, but these infants seemed to be representative of the whole cohort; table 4 tabulates risks of bias. Finally, the Bayley MDI Score used to assess cognitive development has limited accuracy.^20^

**Table 4:** Risks of bias

Type of bias	Judgement ETTNO main trial	Support for judgement (see reference 10)	Judgement This secondary analysis	Support for judgement
Random sequence generation (*selection bias*)	Low risk	‘… randomly assigned. The random sequence was computer generated …’	Low risk	See main trial.
Allocation concealment (*selection bias*)	Low risk	‘Allocation concealment was ensured using sequentially numbered, sealed, opaque envelopes’.	Low risk	See main trial.
Blinding of participants and personnel (*performance bias*)	High risk	‘Caregivers were not blinded to treatment’.	High risk	See main trial.
Blinding of outcome assessment (*detection bias*)	Low-medium risk	‘Outcome assessors (paediatric neurologists, psychologists, ophthalmologists, etc) were not aware of the treatment group.’	Low-medium risk	SpO_2_ data recorded by pulse oximeters are difficult to manipulate. For clinical outcomes, see main trial.
Incomplete outcome data (*attrition bias*)	Low risk	‘The primary outcome of death or neurodevelopmental impairment at 24 months of corrected age was ascertained in 450 (91.5%) patients and 478 (91.7%) patients in the liberal and restrictive threshold groups, respectively’.	Medium risk	Predefined agreement to limit study to infants with recording of >80% of expected SpO_2_ data, which resulted in attrition to 544 of 1013 enrolled infants. Authors argue that analysis of more incomplete SpO_2_ recordings may introduce bias from non-representative SpO_2_ data. The remaining sample appears to be representative for the whole ETTNO cohort based on key risk variables at study entry. The burden of intermittent hypoxaemia is similar in included and excluded infants. Rates of clinical outcomes are also similar to the main cohort.
Selective reporting (*reporting bias*)	Low risk	Study registered before the start of enrolment. Study protocol and statistical analysis plan finalised before the start of analysis published along with the main report.	Low-medium risk	Predefined agreement to limit study to infants with recording of >80% of expected SpO_2_ data. Data were provided for those infants with less complete SpO_2_ recordings in supplement.

## Conclusions

Among infants with birth weights <1000 g, liberal RBCT compared with restrictive transfusions did not affect the burden of IH. Liberal transfusions, while increasing haematocrit and hence oxygen-carrying capacity, did not improve 24 months outcomes in the subgroup of infants with above-median exposure to IH, and no interaction between transfusion thresholds and IH was noted on these outcomes.

Nevertheless, we cannot rule out that a subset of ELBW infants with extremely high exposure to IH while anaemic may benefit from a more liberal transfusion policy than applied in the restrictive group in ETTNO, especially beyond postnatal weeks 5–7. Such a strategy, however, would require careful verification in an appropriately powered trial.

## Supplementary material

10.1136/archdischild-2024-327643 online supplemental file 1

10.1136/archdischild-2024-327643 online supplemental file 2

## Data Availability

No data are available.
