# Supplementary material for: Effects of liberal versus restrictive transfusion strategies on intermittent hypoxaemia in extremely low birthweight infants: secondary analyses of the ETTNO randomised controlled trial
Source: Arch Dis Child Fetal Neonatal Ed. 2025 Mar 26;110(6):e327643. doi: 10.1136/archdischild-2024-327643 (PMC12573360; doi:10.1136/archdischild-2024-327643)
Supplement: online supplemental file 1 [file fetalneonatal-110-6-s001.pdf]

Supplemental Material

Liberal vs restrictive transfusion strategies, intermittent hypoxemia and outcome in preterm infants

Secondary analyses of the ETTNO randomized-controlled trial

Axel R. Franz[1,2], MD; Corinna Engel[1], PhD; Dirk Bassler[3], MD; Mario Rüdiger[4], MD; Ulrich H. Thome[5], MD; Rolf F. Maier[6], MD; Ingeborg Krägeloh-Mann[7], MD; Jochen Essers[8], MD; Christoph Bührer[9], MD; MD; Hans-Jörg Bittrich[10], MD; Claudia Roll[11], MD; Thomas Höhn[12], MD; Harald Ehrhardt[8,13], MD; Ralf Böttger[14] MD; Hans Thorsten Körner[15], MD; Anja Stein[16], MD; Patrick Neuberger[17], Tine Brink Henriksen[18], MD; Gorm Greisen[19], MD; MD; Christian F. Poets[2], MD; for the ETTNO Investigators

eTable 1: **Burden of Intermittent Hypoxemia in Infants Contributing SpO_2 data for ≤80% of Expected Time (During Days 8-49)**

Burden of Intermittent Hypoxemia		
	**Contributing ≤80% of time with continuous SpO_2 data***	
	**Liberal (n=145)**	**Restrictive (n=142)**
**Proportion of time contributing SpO_2 [%]**		
Median (P10-P90)	61.5 (26 – 78)	57 (24 – 77)
**Proportion of Time with SpO_2 <80% [%] only episodes ≥ 60s**		
Median (Q1-Q3)	0.88 (0.13-2.60)	0.71 (0.11-2.68)
**No. of Events with SpO_2 <80% [n] only episodes ≥ 60s**		
Median (Q1-Q3)	121 (20-369)	108 (21-356)
**Mean Duration of Events** ** **with SpO_2 <80% [sec] only episodes ≥ 60s**		
Median (Q1-Q3) (No. of patients analysed)***	120 (100-144) (n=141)	117 (101-147) (n=137)
**Proportion of Time with SpO_2 <60% [%] only episodes ≥ 60s**		
Median (Q1-Q3)	0.02 (0.00-0.09)	0.01 (0.00-0.12)
**No. of Events with SpO_2 <60% [n] only episodes ≥ 60s**		
Median (Q1-Q3)	4 (0-15)	3 (0-22)
**Mean Duration of Events** ** **with SpO_2 <60% [sec] only episodes ≥ 60s**		
Median (Q1-Q3) (No. of patients analysed)***	100 (87-118) (n=94)	103 (83-124) (n=96)

Data is depicted as Median (Quartile1 - Quartile3).

* = Limited to patients with any SpO_2 data

** = Mean duration of events was only calculated in patients with at least 1 event >60s

*** = No. of patients analyzed is provided if deviating from overall number of patients included (excluding patients without event).

Note: 1% time is ~600min (or ~10h) in the 6 weeks observational period (6 weeks =60,480min or 1008h).

eTable 2: **Best Models resulting from Multiple Logistic Regression**

Analyses of full models by backward selection

	Odds-Ratio Estimate (Wald 95%-CI)	p
**Death or Disability (n=496)**		
Gestational Age (<26wks vs. ≥26wks)	2.90 (1.87 - 4.50)	<0.0001
Sex (female vs. male)	0.56 (0.38 - 0.82)	0.0031
Small for Gestational Age ($SDS_{birth\ weight}$ ≥-1.28 vs. <-1.28)	0.43 (0.24 - 0.77)	0.0042
%-time with SpO_2<80% (≤median vs. >median)	0.71 (0.46 - 1.10)	0.1246
**Cognitive Deficit (MDI<85) (n=456)**		
Gestational Age (<26wks vs. ≥26wks)	2.81 (1.74 – 4.53)	<0.0001
Small for Gestational Age ($SDS_{birth\ weight}$ ≥-1.28 vs. <-1.28)	0.42 (0.23 – 0.77)	0.0055
Sex (female vs. male)	0.59 (0.40-0.89)	0.0126
%-time with SpO_2<80% (≤median vs. >median)	0.78 (0.48 - 1.26)	0.3033
**Cerebral Palsy (n=473)**		
%-time with SpO_2<80% (≤median vs. >median)	0.70 (0.31 - 1.62)	0.4101

Multiple logistic regression analyses were performed using factors treatment (liberal vs. restrictive), gestational age at birth (<26 versus ≥26 weeks), sex, small for gestational age (defined as a birth weight standard deviation score <-1.28 versus ≥-1.28), any antenatal corticosteroids (yes/no), multiple birth (yes/no) to identify important predictors / potential influencing factors for all outcome variables using backward selection and retaining burden of intermittent hypoxemia (%-time with SpO_2<80% (limited to events ≥60s) below/above the median).
All influencing factors were evaluated for potential collinearities and interactions (with special interest in the interaction treatment * %time with SpO_2<80%), but no statistically significant collinearities or interactions were identified.

eFigure 1

Distribution of measured SpO_2-values and hemoglobin concentrations by transfusion strategy and week of postnatal age

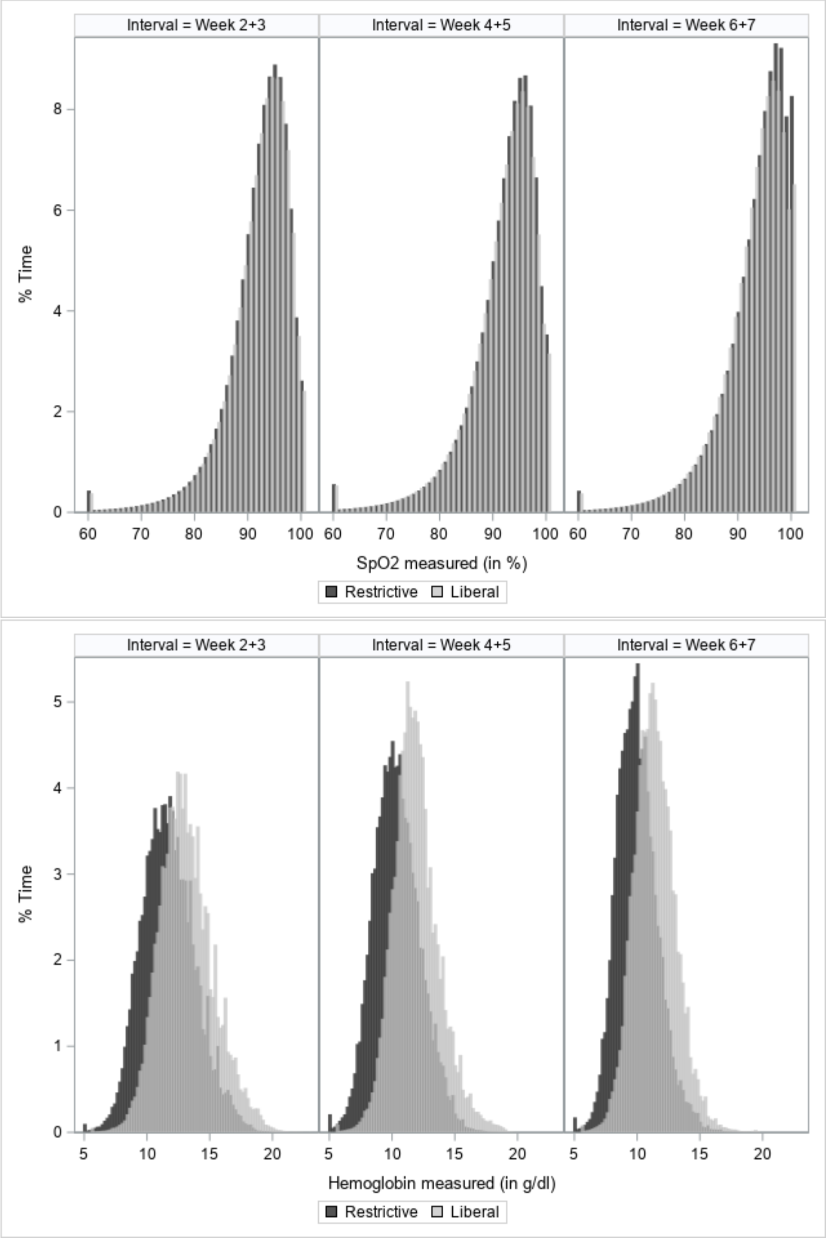

Panel A: %time with individual SpO_2 values (in %) by transfusion strategy and week of postnatal age were virtually identical (and therefore largely overlapping) in both treatment groups. Panel B: %time with individual hemoglobin concentrations (in g/dl) by transfusion strategy and week of postnatal age.
Week = week of postnatal age; restrictive transfusion strategy = dark grey, liberal = light grey. In Panel B, medium grey indicates the overlap of the light grey and dark grey to display differences in the respective frequencies.

eFigure 2: **Exposure to Intermittent Hypoxemia for Infants with and without Adverse Primary Endpoint 'Death or Neurodevelopmental Impairment' by Treatment Group, Gestational Age at Birth, and Postnatal Age**

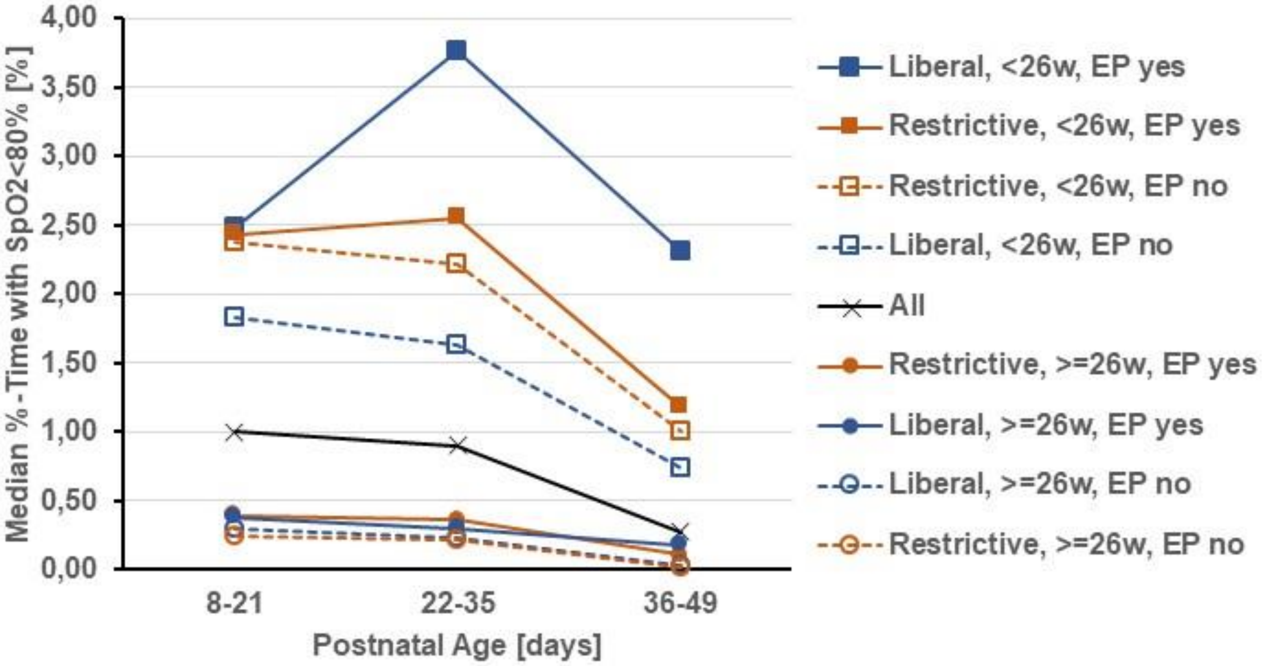

For three postnatal time intervals, the degree of exposure (%-time) to SpO_2<80% limited to episodes ≥60s is depicted for subgroups stratified by transfusion threshold group (liberal versus restrictive), gestational age at birth (< 26 weeks (<26w) versus ≥26 weeks (≥26w)), and presence or absence of the adverse primary endpoint (death or neurodevelopmental impairment) ("EP yes" versus "EP no").

eFigure 3: **Simplified Hypothetical Concept of Prematurity-Related Hypoxic Brain Injury and the Potential Impacts of Intermittent Hypoxemia and Blood Transfusions**

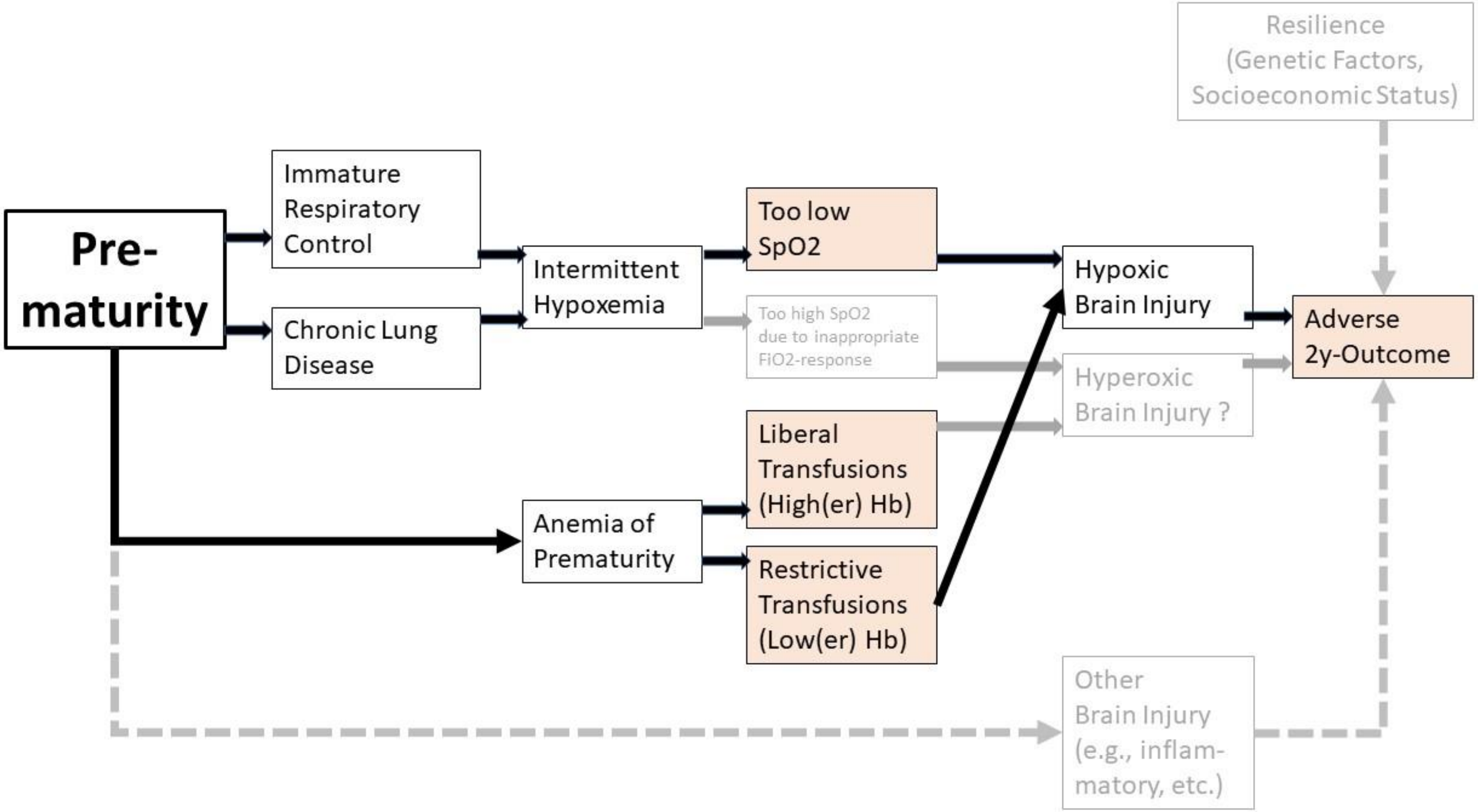

Parameters observed or randomly allocated in this study marked in orange.
