# Supplementary material for: Effects of liberal versus restrictive transfusion strategies on intermittent hypoxaemia in extremely low birthweight infants: secondary analyses of the ETTNO randomised controlled trial
Source: Arch Dis Child Fetal Neonatal Ed. 2025 Mar 26;110(6):e327643. doi: 10.1136/archdischild-2024-327643 (PMC12573360; doi:10.1136/archdischild-2024-327643)
Supplement: online supplemental file 2 [file fetalneonatal-110-6-s002.pdf]

Protocol

Effects of transfusion thresholds on neurocognitive outcome of extremely low birth weight infants (ETTNO)

a blinded randomized controlled multicenter trial

Sponsor's Study Code:	DFG Fr 1455/6-1
EudraCT-Number:	2010-021576-28
Phase:	IV (a study according to §4 para. 23 AMG)
Intervention / Control Intervention:	Implementation of ”liberal” versus ”restrictive” guidelines for red blood cell transfusions in extremely low birth weight infants. Because neither ”liberal” nor ”restrictive” guidelines for red blood cell transfusions can be considered ”standard” therapy in preterm infants, one will serve as control for the other.
Indication:	Treatment of anemia of prematurity and prevention of long-term neuro-developmental sequelae of prematurity
Design:	Prospective, observer-blinded, parallel group, randomized, controlled multicenter trial
Principal Investigator:	Axel Franz, Priv.-Doz. Dr. med.<br>Universitätskinderklinik, Abt. Neonatologie<br>Calwerstr. 7, 72076 Tübingen
Sponsor:	Universitätsklinikum Tübingen<br>represented by M. Bamberg, Prof. Dr. med. and G. Sonntag<br>and delegated Sponsorship to<br>Universitätskinderklinik, Abt. Neonatologie<br>represented by Christian-F. Poets, Prof. Dr. med.<br>Calwerstr. 7, 72076 Tübingen
Version/Date:	1.3 / 18.05.2011

ETTNO is supported by the Deutsche Forschungsgemeinschaft (DFG Fr 1455/6-1).
This protocol is the property of the Pediatric Center for Clinical Studies at the University Children's Hospital of Tuebingen. The recipient agrees that no unpublished information contained herein will be published or disclosed without prior written approval of the Pediatric Center for Clinical Studies and the Principal Investigator, except that this protocol may be disclosed to the appropriate Independent Ethics Committee and the appropriate Authority.

Universitätskinderklinik, Abt. Neonatologie
Calwerstr. 7, 72076 Tübingen

18.05.2011

Date	PD Dr. med. Axel Franz<br>Principal Investigator ETTNO

II. Study Synopsis

**PRINCIPAL AND COORDINATING INVESTIGATOR**	Axel Franz, Priv.-Doz. Dr. med.<br>Universitätskinderklinik, Abt. Neonatologie<br>Calwerstr. 7, 72076 Tübingen<br>Tel. 07071-29-82211 (Pforte), Fax: 07071-29-3969<br>e-mail: axel.franz@med.uni-tuebingen.de
**TITLE OF STUDY**	**Effects of transfusion thresholds on neurocognitive outcome of extremely low birth weight infants (ETTNO)<br>a blinded randomized controlled multicenter trial**
**CONDITION**	Extremely low birth weight, anemia of prematurity, impaired neurodevelopment
**OBJECTIVE(S)**	To compare the effect of restrictive versus liberal red blood cell transfusion thresholds on long-term neurodevelopmental outcome in extremely low birth weight infants
**INTERVENTION(S)**	Experimental intervention: Implementation of ”liberal” versus ”restrictive” guidelines for red blood cell transfusions in extremely low birth weight infants<br>Control intervention: Because neither ”liberal” nor ”restrictive” guidelines for red blood cell transfusions can be considered ”standard” therapy in preterm infants, one will serve as control for the other<br>Follow-up per patient: until 24 months of age corrected for prematurity<br>Duration of intervention per patient: during the initial hospitalization until discharge
**KEY INCLUSION AND EXCLUSION CRITERIA**	Key inclusion criteria: Preterm infants with a birth weight of 400-999g<br>Key exclusion criteria: Missing parental consent, gestational age ≥ 30 weeks, or congenital anomalies
**OUTCOME(S)**	Primary efficacy endpoint: Incidence of death or major neurodevelopmental impairment determined at 24 months of age corrected for prematurity<br>Key secondary endpoint(s): Incidences of the individual components of the composite primary outcome, mental and physical developmental index scores of the Bayley Scales of Infant Development (II edition), growth, and duration of respiratory support and hospital stay<br>Assessment of safety: Incidences of diseases of prematurity, and of all adverse events
**STUDY TYPE**	Prospective, observer blinded, parallel group randomized controlled multicenter trial
**STATISTICAL ANALYSIS**	Descriptive analysis of baseline characteristics.<br>Efficacy: Intention-to-treat (primary) and per-protocol (secondary) analysis of primary and secondary outcome variables by logistic regression if binary and by analysis of variance if quantitative<br>Safety: Safety analysis of all adverse events (including all major diseases of prematurity) based on all individuals included in the study<br>Exploratory analyses: a series of pre-defined subgroup analyses with descriptive statistics and simultaneous evaluation of subgroup variables in logistic regression models
**SAMPLE SIZE**	To be assessed for eligibility: (n = 1415)<br>To be allocated to trial (i.e., randomized): (n = 920)<br>To be analysed: (n = 780, i.e., 2 x 390)
**TRIAL DURATION**	Trial set-up starting: January 2010<br>First patient in to last patient out: July 1st 2011 - September 30th 2015<br>Duration of the entire trial: 5.5 years
**PARTICIPATING CENTERS**	To be involved (n): 29 Level III (German Level I) NICUs

III. Table of Contents

I. Title Page / Signature 1
II. Study Synopsis 2
III. Table of Contents 3
IV. Index of Figures 6
V. List of Abbreviations 7

1. Summary 8
1.1. Plain Word Summary 8
1.2. Key Words 8
1.3. Intervention Scheme / Trial Flow 8

2. Sponsor, Investigators, and Study Administrative Structure 9
2.1. Sponsor 9
2.2. Principal investigator 9
2.3. Steering Committee 9
2.4. Participating Centers and Local Principal Investigators 10
2.5. Independent Data Monitoring Committee 11
2.6. Monitoring 11
2.7. Data Management 11
2.8. Funding 12

3. Introduction / The Medical Problem 13

4. Study Objectives 15
4.1. Primary Endpoint 16
4.2. Determination of the Primary Endpoint 16
4.3. Secondary Endpoints 17
4.4. Determination of the Secondary Endpoints 18

5. Investigational Plan 20
5.1. Overall Study Design and Plan Description 20
5.2. Time Schedule 20

5.3. Study Population 23
5.3.1. Selection of Study Population 23
5.3.1.1. Inclusion Criteria 23
5.3.1.2. Exclusion Criteria 23
5.3.1.3. Justification of Inclusion and Exclusion Criteria 23
5.3.2. Sample Size and Power Calculation 24
5.3.3. No Interim Analysis 24
5.3.4. Compliance / Rate of loss to follow up 24
5.3.5. Feasibility of Recruitment 24
5.3.6. Screening Log and Documentation of Non-Recruitment 25
5.3.7. Removal of Patients from Therapy or Follow-up 26
5.3.8. Premature Withdrawal of a Patient from the Study by the Parents 26
5.3.9. Closure of Study Centers 26
5.3.10. Premature Termination of the Study / Stopping Rules 27
5.3.10.1. Premature Termination of the Study for Safety 27
5.3.10.2. Premature Termination of the Study for Unfeasibility 27
5.3.10.3. Premature Termination of the Study for Efficay 27

5.4. Treatments 28
5.4.1. Investigational Medical Procedures 28
5.4.2. Justification of Investigational and Control Intervention 29
5.4.3. The Investigational Medical Product and its Manufacturer 29
5.4.4. Preparation, Labeling, and Administration of the Investigational Medical Product 30
5.4.5. Storage, Distribution, and Return of the Investigational Medical Product 31

5.5. Methods against Bias 32
5.5.1 Methods of Assigning Patients to Treatment Groups / Randomization 32
5.5.2. Blinding 32
5.5.3. Further Efforts to Avoid Bias 32

5.6. Prior and Concomitant Therapy 33
5.6.1. Delayed Cord Clamping 33
5.6.2. Iron supplementation 33
5.6.3. Protein Supplementation 33
5.6.4. Folic Acid and Vitamin B12 Supplementation 33
5.6.5. Reduction and Documentation of Iatrogenic Blood Loss 34
5.6.6. No Erythropoietin 34
5.6.7. RBCT before Randomization 34
5.6.8. Iron Supplementation after Discharge 34
5.6.9. Co-interventions Proven to Affect Long-term Outcome 34
5.6.10. Oxygen Saturation Targets 34
5.6.11. Other Co-interventions 35

5.7. Treatment and Follow-up after Completion of the Trial 35

5.8. Compliance with Treatment / Study Protocol and Follow-up 35

5.9. Scheduled Examinations 37
5.9.1. Routine Examinations 37
5.9.2. Study-Driven Examinations 39
5.9.2.1. Study-Driven Blood samples 39
5.9.2.1. Study-Driven Urine samples 40
5.9.2.2. Study-Driven Measurements of Cerebral Oxygenation 41

5.10. Documentation 42
5.10.1. Documentation of Raw Data 42
5.10.2. Database, Remote Data Entry, eCRF 42

5.11. Data Management 44

5.12. Data Quality Assurance / Monitoring 45
5.12.1. Monitoring 45
5.12.2. Compliance with the Protocol 46

6. Ethical and Legal Aspects 47
6.1. Ethical Considerations 47
6.2. Risk / Benefit Consideration for the Individual Participant and Potential Additional Burden Caused by Participation in the Clinical Trial 47
6.2.1. Risks and Benefits of Liberal versus Restrictive Transfusion Thresholds 48

6.2.2. Risks and Benefits of Longterm Follow-Up 48
6.3. Ethics Committee (EC) 49
6.4. Ethical Conduct of the Study 49
6.5. Regulatory Authority Approvals/Authorizations/Registration 49
6.6. Subject Information (here: Parents Information) and Consent (here: Parental Consent) 50
6.7. Insurance 51
6.8. Confidentiality 51
6.9. Conflict of Interest 51
6.10. Financial Compensation for Study Participants 52

7. Safety 53
7.1. Independent Data Monitoring Committee (IDMC) 53
7.2. Safety Analyses 53
7.3. Members of the IDMC 55

8. Statistical Methods 56
8.1. Statistical and Analytical Plans 56
8.1.1. Study Populations 56
8.1.2. Description of the Study Population 57
8.1.3. Primary Endpoint 57
8.1.4. Secondary Endpoints 58
8.1.5. Subgroup Analyses 58
8.1.6. Further Explorative Analyses 58
8.1.7. No Interim Analysis for Efficacy 60
8.1.8. Resposible Biostatistician 60

9. Adverse Events 61
9.1. Warnings/Precautions 61

9.2. Adverse Event Definitions according to ICH GCP 61
9.2.1. Adverse Event 61
9.2.2. Serious Adverse Event / Definition of Seriousness 61
9.2.3. Expected Adverse Events 62
9.2.4. Unexpected Adverse Event 62
9.2.5. Relationship of Adverse Event to Investigational Medical Intervention (here: RBCT according to either RBCT guideline) 62
9.2.6. Adverse Reaction 63
9.2.7. Suspected Expected Serious Adverse Reactions (SESARs) 63
9.2.8. Suspected Unexpected Serious Adverse Reactions (SUSARs) 63

9.3. Management of Adverse Events 64

9.4. Documentation of Adverse Events 64

9.5. Reporting of Serious Adverse Events 65
9.5.1. Notification of the Ethics Committee and Competent Authority 65
9.5.2. Information of the Investigators 66
9.5.3. Notification of the Marketing Authorisation Holder 66
9.5.4. Notification Summary 67

9.6. Annual Safety Reports 68
9.6.1. Content of the annual safety report of a clinical trial 68

9.6.2. Reporting time frame for annual safety report 68

10. Use of Data and Publication 69
10.1. Reports 69
10.1.1. Interim Reports 69
10.1.2. Notification of Completion of the Clinical Trial 69
10.1.3. Final Report 69

10.2. Publication 69

11. Changes in the Conduct of the Study or Planned Analysis / Amendments 70

12. List of Appendices 71

13. References 72

IV. Index of Figures

Figure 1: Trial Flow 8
Figure 2: Anticipated Study Milestones 21
Figure 3: Study Time Table 22

V. List of Abbreviations

AE	Adverse Event
BPD	Bronchopulmonary Dysplasia
CPAP	Continuous Positive Airway Pressure
CPCS	Center for Pediatric Clinical Studies
CRF	Case Report Form
EC	Ethics Committee
ELBW	Extremely Low Birth Weight
GA	Gestational Age
GMFCS	Gross Motor Function Classification Scale
IDMC	Independent Data Monitoring Committee
ITT	Intention-To-Treat
IVH	Intraventricular Hemorrhage
MDI	Mental Developmental Index
NEC	Necrotizing Enterocolitis
NICU	Neonatal Intensive Care Unit
PDA	Patent Ductus Arteriosus
PDI	Physical Developmental Index
PEI	Paul Ehrlich Institut
PP	Per-Protocol
PVL	Periventricular Leukomalacia
RBCT	Red blood cell transfusion
ROP	Retinopathy of Prematurity
SAE	Serious Adverse Event
SCPE	Surveillance of Cerebral Palsy in Europe
SESAR	Suspected Expected Serious Adverse Reaction
SUSAR	Suspected Unexpected serious Adverse Reaction
TRALI	Transfusion Related Acute Lung Injury

1. Summary

1.1. Plain Word Summary

Extremely low birth weight (ELBW) infants uniformly develop anemia of prematurity and frequently require multiple red blood cell transfusions (RBCT) during neonatal intensive care. The criteria currently applied to indicate RBCT in this population are based on expert opinion rather than evidence and conclusive data of long-term effects of RBCT practices do not exist. Both, giving RBCT to improve oxygen carrying capacity and restricting RBCT to avoid RBCT associated risks and costs potentially impair long-term development. The proposed blinded randomized controlled trial was designed and will be powered to compare the effect of restrictive versus liberal red blood cell transfusion guidelines on long-term neurodevelopmental outcome in ELBW infants. ELBW infants will be randomized to receive RBCT according to liberal or restrictive RBCT guidelines, which both reflect current practice in Germany and aim for a clinically relevant difference in mean hemoglobin concentrations. The primary outcome measure is the incidence of death or major neurodevelopmental impairment determined at 24 months of age corrected for prematurity. Key secondary outcomes are the incidences of individual components of the composite primary outcome, the mental and physical developmental index scores of the Bayley Scales of Infant Development (II edition), and growth. Safety analyses will assess the incidences of all major diseases of prematurity. The results of this trial may help to improve the quality of life of these patients and reduce long-term health care costs.

1.2. Key Words

extremely low birth weight infant, anemia of prematurity, blood transfusion, neurodevelopmental outcome

1.3. Intervention Scheme / Trial Flow

Figure 1: Trial Flow

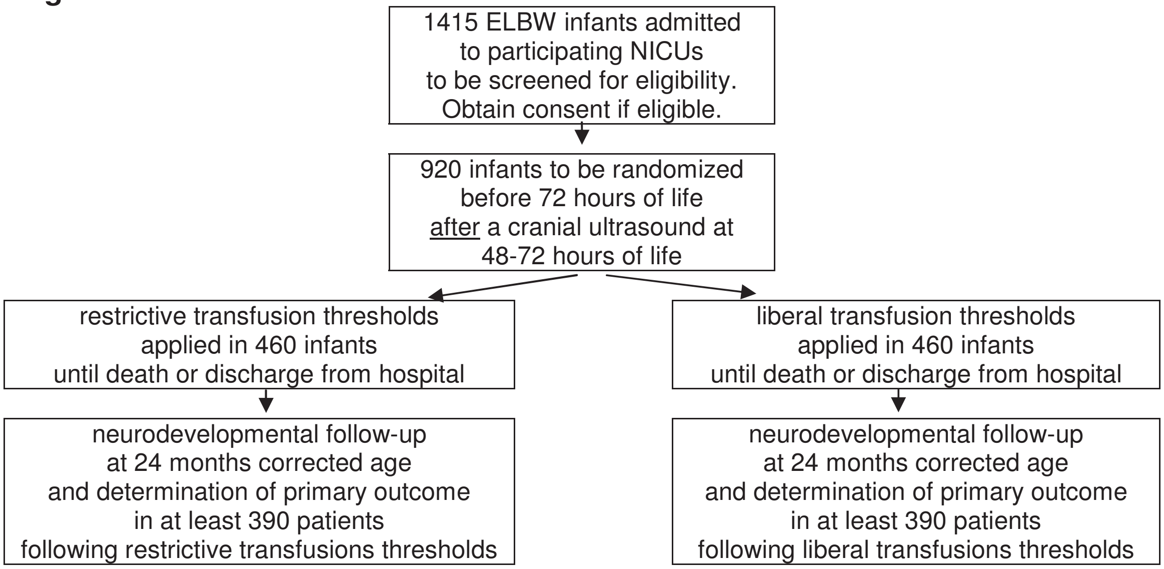

2. Sponsor, Investigators, and Study Administrative Structure

2.1. Sponsor

The Sponsor is:

Universitätsklinikum Tübingen
represented by Prof. Dr. med. M. Bamberg and G. Sonntag
Geissweg 3
72076 Tübingen

and delegated Sponsorship to:
Abt. Kinderheilkunde IV, Neonatologie
represented by Prof. Dr. med. Christian-F. Poets
Calwerstr. 7
72076 Tübingen

2.2. Principal investigator

The Principal Coordinating Investigators is:

PD Dr. med. Axel Franz
University of Tuebingen
Dept. of Pediatrics, Neonatology
Calwerstraße 7
72076 Tübingen
telephone: +49-7071-29-0 (ask to page Dr. Franz at #2322)
fax: +49-7071-29-3969
e-mail: axel.franz@med.uni-tuebingen.de

He is responsible for the overall trial conduct and decisions; i. e., he has the responsibility as sponsor of the trial.

2.3. Steering Committee

Central project management will be supported by the Principal Co-Investigators, who form together with the Principal Coordinating Investigator and the Trial Statistician the Steering committee:

PD Dr. med. Dirk Bassler
University of Tuebingen
Dept. of Pediatrics, Center for Pediatric Clinical Studies
Calwerstraße 7
72076 Tübingen
telephone: +49-7071-29-0 (ask to page Dr. Bassler at #2299)
fax: +49-7071-29-3969
e-mail: dirk.bassler@med.uni-tuebingen.de

Prof. Dr. med. Rolf Maier
University of Marburg
Neonatology and Neuropediatrics
Baldingerstraße
35043 Marburg
telephone: 49-6421-28-66229
fax: 49-6421-28-68970
e-mail: rolf.maier@med.uni-marburg.de

Prof. Dr. med. Christian-F. Poets
University of Tuebingen
Dept. of Pediatrics, Neonatology
Calwerstraße 7
72076 Tübingen
telephone: +49-7071-29-84742
fax: +49-7071-29-3969
e-mail: christian-f.poets@med.uni-tuebingen.de

Prof. Dr. med. Ingeborg Krägeloh-Mann
University of Tuebingen
Dept. of Pediatrics, Neurology
Hoppe-Seyler-Str. 1
72076 Tübingen
telephone: +49-7071-29-84735
fax: +49-7071-29-5473
e-mail: ingeborg.kraegeloh-mann@med.uni-tuebingen.de

Prof. Dr. med. Mario Rüdiger
University of Dresden
Dept. of Pediatrics, Neonatology
Fetscherstraße 74
01307 Dresden
telephone: +49-351-458-3640
fax: +49-351-458-5358
e-mail: mario.ruediger@uniklinikum-dresden.de

Prof. Dr. med. Ulrich Thome
University of Leipzig
Dept. of Pediatrics, Neonatology
Liebigstraße 20a
04103 Leipzig
telephone: +49-341-97-26021
fax: +49-341-97-23579
e-mail: ulrich.thome@medizin.uni-leipzig.de

Statistician: Prof. Dr. biol. hum. Martina Kron
University of Ulm
Dept. of Biometry and Medical Documentation
89070 Ulm
telephone: 49-731-500-26904
fax: 49-731-500-26902
e-mail: martina.kron@uni-ulm.de

2.4. Participating Centers and Local Principal Investigators:

The list of participating centers and local principal investigators is attached in the appendix.

The local principal investigators have the responsibility for the conduct of the trial according to the protocol and for the neonatal care of the patient. The trial will involve 25 German level III (German level I) neonatal centers.

2.5. Independent Data Monitoring Committee

Members of the Independent Data Monitoring Committee (IDMC) will be designated by the steering committee. The IDMC is established to assess the progress of the clinical trial and safety issues. The IDMC is not responsible to assess critical efficacy endpoints because first measurements of endpoints will be performed when recruitment and study treatment have been terminated. The IDMC is independent from the Steering Committee as well as from the supporting parties. The IDMC monitors the cumulative safety data for evidence of treatment harm and benefit. The IDMC may give advice in the interest of health care to continue, modify or terminate the trial. Modification of the trial might be e.g., termination of a treatment arm of the trial before complete recruitment of patients because of unacceptably high rates of serious adverse events.
Also, unfeasibility for successful termination of the study may lead to premature termination. (For reasons see also section 4.5.2). Throughout the study the IDMC will especially monitor the incidence rates of suspected unexpected serious adverse reactions (SUSAR) and the incidence rates of serious adverse events (SAE). Analysis of these data will be performed by an independent statistician who is not involved in this trial and who is member of the IDMC. The IDMC will not otherwise be involved in the conduct of the trial.
The IDMC members are listed separately.

2.6. Monitoring:

The steering committee assigned the Center for Pediatric Clinical Studies (CPCS) with the conduct of the monitoring of the trial. The infrastructure and personal for data management will be provided by the

Center for Pediatric Clinical Studies
Dr. Joachim Riethmüller
University of Tuebingen
Dept. of Pediatrics
Hoppe-Seyler-Str.1
72076 Tübingen
telephone: +49-7071-29-81391
fax: +49-7071-29-4450
e-mail: joachim.riethmueller@med.uni-tuebingen.de

The CPCS will assign qualified personal for onsite monitoring that is not otherwise involved in the conduct of the trial.

2.7. Data Management:

The infrastructure and personal for data management will also be provided by the

Center for Pediatric Clinical Studies
Biometry
Dr. Corinna Engel
University of Tuebingen
Dept. of Pediatrics,

Hoppe-Seyler-Str.1
72076 Tübingen
telephone: +49-7071-29-89111
fax: +49-7071-29-NN
e-mail: corinna.engel@med.uni-tuebingen.de

The CPCS will assign qualified personal for data management and provide adequate software support.

2.8. Funding

Funding will be provided within the joined project “Klinische Studien” of the Deutsche Forschungsgemeinschaft (DFG) and the Bundesministerium für Bildung und Forschung (BMBF). Grant code is DFG Fr1455/6-1.
The ancillary study A, „Effects of intermittent hypoxemic episodes *and* anemia on neurocognitive outcome” is supported with pulse oximeter equipment by Masimo Corp. Irvine, CA.

3. Introduction / The Medical Problem

Extremely low birth weight (ELBW) infants uniformly develop anemia of prematurity and frequently require multiple red blood cell transfusions (RBCT) during neonatal intensive care (1-3). The criteria currently applied to indicate RBCT in this population are based on expert opinion rather than evidence and conclusive data of long-term effects of RBCT practices do not exist. Both, giving RBCT to improve oxygen carrying capacity and restricting RBCT to avoid RBCT associated risks and costs potentially impair long-term development.

Although RBCT are a universally accepted part of the treatment of preterm infants, RBCT practices vary greatly between neonatal intensive care units (NICUs) in the absence of uniformly accepted physiologic or evidence-based RBCT criteria (1; 4-8).
The short-term effects of RBCT on outcome measures such as apnea, weight gain, heart rate, and oxygen consumption have been studied in a few small randomized, mostly uncontrolled trials and several observational studies with controversial results (reviewed in (9)) – conclusive data of long-term effects of RBCT practices do not exist according to recent systematic reviews (10).
Reducing the number of RBCT will reduce the risk of transmission of Cytomegalo, Hepatitis, Human Immune Deficiency Viruses, and other infectious agents (11) and may reduce costs. Since frequent RBCT may be associated with retinopathy of prematurity (ROP) (12-14) and bronchopulmonary dysplasia (BPD) (15; 16), reducing RBCT may be even more important in this vulnerable population. Reducing RBCT will also reduce the incidence of all transfusion associated complications which are 3 times more common in infants than in adults (17). Although transfusion related lung injury (TRALI) (18) has yet been unreported in preterm infants, these infants may indeed be at high risk of this complication because pulmonary endothelial activation and neutrophil sequestration are common findings in respiratory distress, ventilator-induced lung injury, and bronchopulmonary dysplasia, and the transfusion of biologic response modifiers (including lipids contained in red blood cell concentrates) may activate these adherent neutrophils, resulting in endothelial damage, capillary leak, and TRALI (19). A very preliminary, single-center analysis of RBCT in newborn infants observed as many as 23 acute pulmonary deteriorations in 148 episodes of RBCT (20), emphasizing the need for cautious application and careful re-evaluation of RBCT in this age group.
It has been shown previously, that ‘restrictive’ RBCT guidelines effectively reduce the number of RBCT administered to preterm infants (21), and we have previously shown that ‘restrictive’ RBCT guidelines are feasible in very low birth weight infants and may result in very low numbers and low cumulative volumes of RBCT similar to those reported in studies on erythropoietin administration in these infants (22).
However, reducing RBCT by accepting low hemoglobin concentrations carries the risk of at least temporarily insufficient oxygen transport to vital organs and impaired outcome. There is no evidence that achieving a reduction in RBCT by accepting ‘restrictive’ RBCT guidelines (i.e., by accepting very low hemoglobin levels) is safe and ultimately benefits the patient.
In a recent randomized trial of restrictive versus liberal RBCT guidelines in 100 infants with a birth weight of 500-1300g, restrictive RBCT guidelines were associated with a marginally increased incidence of apnea (23). The study did not provide data on long-term neurocognitive outcome but the authors reported an increased incidence of brain injury (intraventricular hemorrhage (IVH) grade 4 or periventricular leukomalacia (PVL)) with restrictive RBCT guidelines. The study was criticized because a) the primary endpoint for sample size determination was the number of transfusions given and not a

parameter of outcome, b) the combined outcome of IVH 4° and PVL had not been a pre-defined outcome measure, c) IVH usually occurs at a time when infants had not yet been enrolled into that study, d) there was an excess of male patients in the restrictive transfusion group and male infants tend to have poorer outcomes, e) only 52% of the patients had a late cranial ultrasound required to assess the true incidence of PVL (24-26).
In another recent randomized trial of restrictive versus liberal RBCT guidelines in 451 infants with a birth weight of <1000g, restrictive RBCT guidelines were associated with fewer transfusions but no differences in the primary outcome of death or survival with ROP≥3, BPD, or brain injury on ultrasound was found (27). This study was criticized for the fact that the mean difference in hemoglobin levels between both treatment groups was only marginal with 1g/dl and that the mean hemoglobin levels were high in both groups (10g/dl versus 11g/dl), and did not reflect the range of RBCT guidelines currently proposed (28). Neurodevelopmental outcome of these infants at 18-22 months corrected age was reported very recently (29): there was no statistically significant difference in the combined outcome of death or severe adverse neurodevelopmental outcome. However, posthoc analyses showed that the proportion of infants with cognitive delay defined as a Mental Developmental Index (MDI) <85 was lower and the mean MDI score was higher in the liberal transfusion group, raising concern that restrictive transfusion guidelines may result in impaired neurodevelopment.

Because oxygen transport is not only compromised during anemia but also during intermittent hypoxemic episodes which are extremely common in very preterm infants, there may be an interaction between the frequency, severity, and duration of these hypoxemic episodes and the degree of anemia affecting neurodevelopmental outcome. However, there is no data to assess the combined effect of intermittent hypoxia and anemia on long-term outcome of preterm infants so far.

In conclusion, there is insufficient evidence to decide whether preterm infants should be treated according to liberal or restrictive RBCT guidelines (10). The long-term safety and efficacy of 'restrictive' RBCT practices can only be evaluated in an adequately powered, large, randomized, controlled trial with long-term neurodevelopmental follow-up and with a sufficient difference in mean hemoglobin levels between both treatment arms to reflect the range of RBCT guidelines currently applied.
Furthermore, data is needed 1) to assess the effect of hypoxemic episodes on the neurodevelopmental outcome in preterm infants with different degrees of anemia, 2) to assess whether alternative markers indicating insufficient oxygen transport are more predictive of a clinical benefit from RBCT than the hematocrit itself, 3) to evaluate whether the above described association of frequent blood transfusions with diseases of prematurity is indeed caused by an increased load of free oxygen radicals and hence associated with an increased production peroxidation products, and 4) elucidate the incidence of transfusion complications and particularly TRALI in ELBW infants. These later subordinate research questions are addressed in ancillary studies of the ETTNO trial also described herein.

4. Study Objectives

This study was designed to answer the following primary (i.e., a)) and secondary (i.e., b)) research questions:

Primary outcome:
a) Do 'liberal' RBCT practices that intend to keep the hematocrit levels ≥ 28% at all time during the initial hospitalization improve or impair long-term outcome (i.e., reduce or increase the incidence of death or major neurodevelopmental impairment evaluated at 24 months corrected age) in extremely low birth weight infants if compared with 'restrictive' RBCT guidelines that accept hematocrit levels as low as 21% (according to the RBCT guidelines described in detail below)?

Secondary outcomes:
b) Do RBCT-guidelines have an effect on short-term outcomes such as in-hospital growth and mortality, and the incidences of the major diseases of prematurity (i.e., BPD, ROP, necrotizing enterocolitis (NEC), intestinal perforation, brain injury on cranial ultrasound, and patent ductus arteriosus (PDA) requiring therapy), or long-term outcomes such as growth and mortality until follow-up, the mental and physical developmental index scores, and the incidences of the individual components of the primary composite outcome?

Trying to optimize the gain of knowledge, ETTNO includes a series of ancillary studies which address the following subordinate research questions (i.e., sub-a – sub-d). Participation in the ancillary studies B, C, and D is optional depending on manpower and device availability in each center:

Ancillary study A ("Effects of intermittent hypoxemic episodes *and* anemia on neurocognitive outcome") aims to answer the following research question:
sub-a1) Do RBCT guidelines and resulting hemoglobin concentrations have an impact on the effect of intermittent hypoxemic episodes on neurocognitive outcome or retinopathy of prematurity?
sub-a2) Are low oxygen content rather than low oxygen saturation values indicative for poor neurocognitive outcome or retinopathy of prematurity.

Ancillary study B ("Better indicators than hematocrit to predict a short-term clinical benefit from RBCT") aims to answer the following research question:
sub-b1) Do concentrations of VEGF (30) in plasma and urine predict the "need of RBCT" (defined as a pre-defined response of clinical signs of anemia to the RBCT) with a higher precision than the hematocrit (the concentration of hemoglobin)?
sub-b2) Does the cerebral oxygen saturation measured by near infrared spectroscopy (31) predict the "need of RBCT" (defined as a pre-defined response of clinical signs of anemia to the RBCT) with a higher precision than the hematocrit (the concentration of hemoglobin)?

Ancillary study C ("Effects of transfusion thresholds on urinary peroxidation products") aims to answer the following research question:
sub-c) Do RBCT-guidelines influence markers of oxidative stress and are such markers related to complications of prematurity (ROP, BPD, ventricular dilatation, etc.) or neurocognitive outcome?

Ancillary study D ("Evaluation of the incidence of transfusion complications and particularly transfusion related lung injury (TRALI) in preterm infants") aims to answer the following research question:

sub-d) What is the incidence of transfusion complications and TRALI in ELBW infants?

4.1. Primary Endpoint

The primary outcome measure of this study will be the long-term neurocognitive development measured as the incidence of death or major neurodevelopmental impairment determined at 24 months of age corrected for prematurity (where major neurodevelopmental impairment is defined as any of the following: cognitive delay defined as mental developmental index (MDI) score of the Bayley Scales of Infant Development (2nd edition) < 85, cerebral palsy, or severe visual or hearing impairment (as defined below)).

The long-term neurocognitive development of preterm infants determines their ability to lead their lives independently without assistance. In a recent review on outcome measures in randomized controlled trials in preterm infants, long-term neuro-developmental outcome measures were clearly favored as most meaningful (32). The working group 'Frühe Hirnläsionen' of the German 'Fachgesellschaft' for Neuropediatrics and the working goup 'Neuroprotektion' (including Neonatologists, Perinatologists and Neuropediatricians) recommended a systematic neurocognitive evaluation of preterm infants applying the Bayley Scales of Infant Development (2nd edition) at 24 months corrected age (33). At this age, most preterm infants will walk and the MDI score will be reasonably predictive for very longer-term outcome and overall academic achievements.

The cut-off of MDI<85 for the definition of a cognitive delay is meaningful, as infants who remain at a level of more than one standard deviation below the mean are likely to fail or will at least have major difficulties with regular school education. If an intervention increases the proportion of infants above this cut-off, it will likely reduce the need for special education and life-long assistance for these individuals and thereby substantially reduce special education and social care costs for society.

Because the MDI score may fail to capture important competing outcome events such as death or severe physical or neurosensory impairments that preclude psychomotor testing, a composite outcome measure (also including death, severe visual impairment, severe hearing impairment, and cerebral palsy) will be evaluated as primary endpoint.

4.2. Determination of the Primary Endpoint

Study children will be assessed at 23 to 25 months corrected age. Outcome assessments will be conducted by centrally trained pediatric neurologists and certified psychologists, who are blinded to the patients' group assignment. To determine neurodevelopmental outcome, all components of the Bayley Scales of Infant Development (2nd edition) will be applied from which the mental developmental index (MDI) is selected for evaluation of cognitive function, and cognitive delay will be defined as an MDI<85 for the primary outcome.

Children whose severe cognitive impairment or disability precludes the use of the Bayley Scales of Infant Development will be assigned an MDI score of 30 if minimal speech and the ability for minimal communication with the parents are present, and a MDI score of 20 if no speech is present but at least minimal sensory or motor achievements are elicited.

For children lost to follow-up, a score will be imputed based on details obtained from the Pediatrician caring for the infant: severely retarded infants whom the Pediatrician rates

as non-testable will be imputed by 30 if minimal speech and the ability for minimal communication with the parents are present, and by 20 if no speech is present but at least minimal sensory or motor achievements are elicited. Scores of 50 – 80 will be allocated according to a-priori determined criteria in the assessment of the Pediatrician.
A general history and a physical and neurological examination will be used to determine the presence of cerebral palsy. Cerebral palsy will be diagnosed if the child has a non-progressive motor impairment characterized by abnormal muscle tone and impaired range or control of movements, according to the criteria defined by the European network 'Surveillance of CP in Europe' (34; 35). The Gross Motor Function Classification System (GMFCS) (36) will be used for the functional assessment of cerebral palsy.

A visual and hearing assessment will also be performed. Severe visual impairment will be defined as the best corrected vision in the better eye of visual acuity of 6m/60m. Severe hearing impairment was defined as a hearing loss requiring amplification or the insertion of a cochlear implant.

The composite primary outcome will be considered present if 1 or more of the individual components of the composite outcome are known to be present, or absent if all components are known to be absent. If no component was present, 1 or more missing components will cause the primary outcome to be deemed missing.

4.3. Secondary Endpoints (including endpoints of ancillary studies)

Secondary endpoints are the individual components of the composite primary outcome described above, the incidence of cognitive delay defined as MDI<70 (to match the more restrictive anglo-american definition), the MDI score, and the physical developmental index (PDI) score.

Further secondary endpoints are measures of growth at discharge, length of hospital stay, and the time intervals to the last discontinuation of positive pressure respiratory support, respiratory stimulation with methylxanthines, and gavage feeding.

Further analyses will be performed to detect differences in the incidence of adverse events including all major diseases of prematurity (i.e., BPD, ROP, necrotizing enterocolitis (NEC), intestinal perforation, brain injury on cranial ultrasound, and PDA requiring therapy, nosocomial infections) between the treatment groups during the treatment phase – these analyses have also been reported by previous trials (23; 27).

Finally, follow-up at 5.5-6 years of age is also intended and a separate protocol and fund application will be developed as soon as appropriate.

Endpoints of ancillary study A)
Neurocognitive outcome as described in 4.1. (i.e., the primary endpoint of ETTNO and the individual components thereof).

Endpoints of ancillary study B):
Concentrations of VEGF in plasma and parameters of cerebral oxygenation determined at the time of RBCT, along with the clinical response to RBCT.

Endpoints of ancillary study C)

Markers of oxidative stress determined from weekly urine samples throughout the study.

Endpoints of ancillary study D)
Incidence of transfusion reactions, particularly respiratory and gastrointestinal morbidity associated with RBCT.

4.4. Determination of the Secondary Endpoints (including endpoints of ancillary studies)

The individual components of the primary endpoint are determined as described in 4.2.

Growth will be determined as length, weight, and head circumference at 36 weeks postmenstrual age and will be uniformly converted to standard deviation scores.

The length of hospital stay will be determined in days from birth to final discharge home including the duration of care after referral to other centers.

The duration of positive pressure respiratory support will be determined in days from birth until last discontinuation of CPAP or mechanical ventilation, and will not take into account periods of positive pressure respiratory support for less than 48 hours associated with a surgical intervention (e.g., inguinal herniotomy before discharge) or immunizations.

The duration of respiratory stimulation with methylxanthines will be determined in days from birth until last discontinuation of caffeine, caffeine citrate, or theophylline, and will not take into account respiratory stimulation with methylxanthines for less than 48 hours associated with a surgical intervention (e.g., inguinal herniotomy before discharge) or immunizations.

The duration of gavage feeding will be determined in days from birth until last discontinuation of gavage feeding prior to discharge.

The incidence of treated PDA will be measured. Indomethacin, ibuprofen, and surgical ligation will be considered as treatment. Prophylactic indomethacin within the first 3 days of life will not be considered as treatment, but will be recorded as baseline variable (as intervention before randomization). The decision to treat a PDA will be left to the local physician.

NEC (modified Bell stage ≥ IIA) or intestinal perforation will be diagnosed at surgery, at autopsy, or by either the finding of pneumatosis intestinalis, hepatobiliary gas, or free intraperitoneal air on abdominal x-ray, or by demonstration of gas bubbles in the portal vein on abdominal ultrasound.

ROP will be diagnosed at routine ophthalmologic examinations, beginning at a postmenstrual age of 32 weeks. The severity of ROP will be graded according to the international classification (37). The most severe grade of ROP as well as any treatment for ROP will be recorded, and the incidence of ROP > grade 2 and of ROP requiring therapy according to the guidelines of the German Societies of Ophthalmology and Neonatology will be determined.

The presence of BPD will be determined at 36 weeks postmenstrual age according to the physiological definition of Walsh et al. (38).

The presence of brain injury on cranial ultrasound will be evaluated centrally by personnel who is blinded to the patients' treatment group assignment on brain ultrasounds performed at 48-72h (for baseline description), at 7 and 28 days of life, and at discharge, based on: presence of germinal matrix or intra-ventricular hemorrhage (according to the classification of Papile (39)), intra-parenchymal echodensity, porencephalic cyst, periventricular leukomalacia (according to the classification of De Vries (40)), or ventriculomegaly on the "worst" cranial ultrasound.

The incidence of nosocomial infection will be measured. Blood culture-proven sepsis, clinical sepsis, and pneumonia are recorded according to the German NEOKISS infection surveillance criteria.

Endpoints of ancillary study A)
The parameters of neurocognitive outcome are determined as described in 4.2.

Endpoints of ancillary study B):
The response to RBCT as an indicator for a degree of anemia indeed requiring transfusion will be defined by the presence of at least 2 of the following:

a) mean HR during 24 hours post-transfusion is more than 15% decreased if compared to the mean HR during 24 h pre-transfusion
b) did the number of desaturations to less than 80% (irrespective to pathophysiology) are decrease by >20% (and at least by more than 5 episodes) during the during 24 hours post-transfusion if compared to the number of desaturations to less than 80% during 24 hours pre-transfusion
c) weight gain increased by >10g/day during the during the 7 days post-transfusion if compared to the 7 days pre-transfusion
d) the amount of milk fed by bottle (rather than by NG-tube) increased by >40ml/kg/d during the 7 days post-transfusion if compared to the 7 days pre-transfusion
e) a decrease in serum lactate from values > 3mmol/l during 24 h pre-transfusion to values < 2mmol/l during the during 24 hours post-transfusion.

Endpoints of ancillary study C)
Urinary concentrations of Malondialdehyde, 3-Nitrotyrosine, 2-Hydroxynonenal, 8-Isoprostane, and 8-Hydroxy-2'-desoxyGuanosin of weekly urine samples will be determined by tandem-mass spectrometry available at the University of Marburg and at the University of Tuebingen by personnel who is blinded to the patient's treatment group assignment and outcome.

Endpoints of ancillary study D)
The incidence of transfusion reactions (temperature instability, tachycardia (>180/min), arterial hypotension requiring intervention, deterioration of gas exchange, or rash in immediate timely association with a RBCT) will be recorded.

Furthermore, the incidence of gastrointestinal morbidity and confirmed NEC (confirmed at surgery, autopsy, by the presence of pneumatosis intestinalis, hepatobiliary gas, free intraperitoneal gas) during the 48 hours pre- and post-transfusion will be documented.

5. Investigational Plan

5.1. Overall Study Design and Plan Description

This is an observer-blinded, randomized, controlled, parallel group multicenter trial.

920 preterm infants with a birth weight of 400-999g will be randomized on day 3 of life to either restrictive or liberal transfusion thresholds during their initial hospitalization which will trigger red blood cell transfusions either at lower or higher hematocrit (or haemoglobin) values. The primary outcome will be a composite of death or neurodevelopmental impairment determined by trained personal who are blinded to the patients' treatment group assignment at 24 months of age corrected for prematurity. Central evaluation of head ultrasounds by a pediatric radiologist blinded to the patients' treatment group assignment will reduce bias in this key secondary outcome measure.

Allocation concealment will be ensured using consecutively numbered sealed opaque envelops which have been checked by diaphanoscopy for completely obscuring treatment allocation. The random sequence lists are created using the software „Randlist Version 1.2“ created by DatInf GmbH Tuebingen and stored securely at a password protected drive at the Center for Pediatric Clinical Trials at the University Children's Hospital of Tuebingen which is not accessible for the personal involved in the conduct of the trial. The envelopes are filled and verified by personal also not involved in the study at the Center for Pediatric Clinical Trials at the University Children's Hospital of Tuebingen.

5.2. Time Schedule

Time schedule:

Trial set-up starting:	01.01.2010
First patient in:	01.07.2011
Inclusion last patient (Randomisation):	30.06.2007
Last patient out:	30.09.2015
Database closure:	31.12.2015
Termination statistical analysis:	31.03.2016
Study report:	31.03.2016

A detailed description of anticipated milestones is depicted on the following page in figure 2.

Figure 2: Anticipated Study Milestones

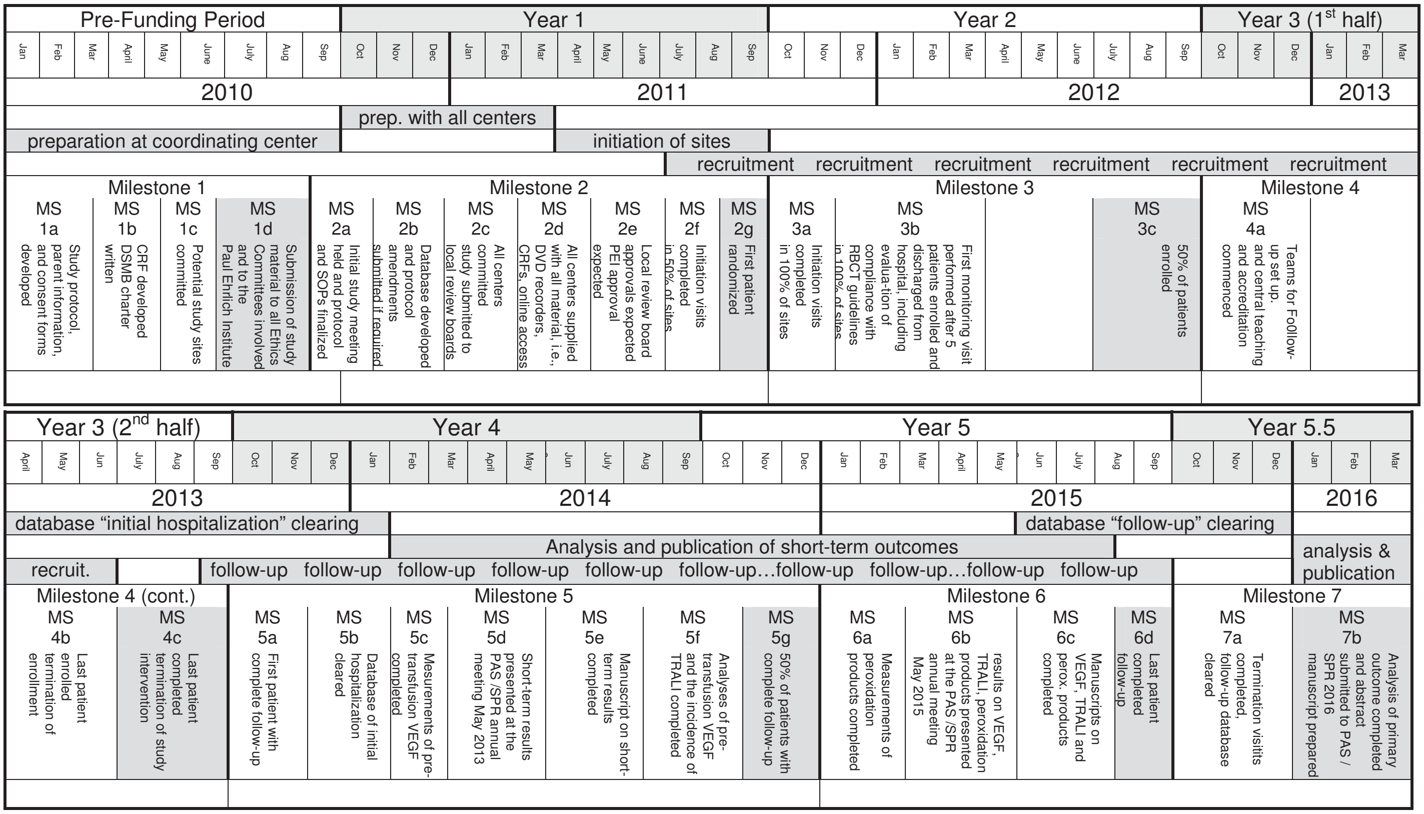

Figure 3: Study Time Table for the individual patient

AGE	Day of Life				Discharge	3-6 months corrected age	12 months	24 months corrected age
	1-2	48-72 hours	3	…				
	Hospital Admission							
ACTION	Screen for eligibility.<br><br>If eligible: approach & inform parents.	Perform cranial ultrasound<br><br>Randomize patient if still eligible and if consent was obtained	Follow RBCT guidelines	Follow RBCT guidelines until discharge from hospital in all patients.<br><br>In all patients until discharge:<br>Iron and protein supplementation as detailed below.<br><br>Neonatal care according to awmf- and center guidelines as detailed below.<br><br>Monitor blood count, growth, markers of oxidative stress, … as detailed below.<br><br>Document all adverse events as detailed below.	Remind parents of FU.<br><br>Inform Pediatrician about study and FU.<br><br>Continue iron supple-mentation until 12 months as recommen-ded.	Call parents<br><br>Fill in 6-months questionnaire<br><br>Remind parents of FU.	Send birth day card and 12- months questionnaire along with free return envelope.<br><br>Remind parents of FU.	Obtain anthropo-metric data.<br><br>Perform formal neurological examination.<br><br>Perform Bayley Scales of Infant development.<br><br>Measure visual acuity.<br><br>Fill in 24-months questionnaire.

5.3. Study Population

5.3.1. Selection of Study Population

5.3.1.1. Inclusion Criteria

- Infants with a birth weight of 400 - 999g,
- male or female,

5.3.1.2. Exclusion Criteria

- Missing written parental consent.
- Gestational age > 29 + 6/7 weeks
- Major congenital anomalies (including chromosomal aberrations, cyanotic congenital heart defects, syndromes likely affecting long-term outcome, and major congenital malformations requiring surgical correction during newborn period).
- Infants who died before 48 hours, infants in whom the clinical decision to withhold intensive care was made, infants who were not considered viable
- Participation in another study with ongoing use of an unlicensed investigational product from 28 days before study enrollment until the end of the study

5.3.1.3. Justification of Inclusion and Exclusion Criteria

The research question underlying this clinical trial can not be answered by studying animal or adult humans, but can only be answered by a study in the population concerned: i.e, premature infants at risk of both, frequent blood transfusions for anemia of prematurity and impaired neurodevelopmental outcome. The results of the trial may help to improve long-term outcome of premature infants and to reduce society's health care costs.

The study population (infants with a birth weight of 400 - 999g) will consist of the vast majority of extremely preterm infants at risk for the need of multiple RBCT and impaired neurocognitive development. Birth weight rather than gestational age limits were chosen, because (a) birth weight can be determined more reliably and (b) blood volume and iron stores at birth, which are both major determinants of transfusion requirements, are related to birth weight rather than gestational age (41; 42).

Infants with a birth weight of <400g are excluded to increase the homogeneity of the patient population: There will only be very few infants with a birth weight <400g during the study period and they are not representative for heavier extremely low birth weight infants because they suffer extremely high mortality and complication rates and neurocognitive impairment is extremely common in these infants – and both high mortality and disability rates are predominantly un-related to transfusions thresholds.

Infants with a birth weight ≥ 1000g rarely receive blood transfusions today and are at a much lower risk of impaired neurodevelopment and would therefore dilute any treatment effect. On the other side, transfusion limits proven to be safe at the end of the proposed study will with high certainty also be safe for these less fragile infants and can therefore be extrapolated to them.

ELBW Infants with a gestational age ≥30 weeks are excluded (a) because these infants likely do not require any transfusions at all and (b) because any long-term neurodevelopmental impairment is likely caused by severe intrauterine growth retardation (43) and not by the effect of transfusion guidelines.

Infants with congenital anomalies (defined as chromosomal anomalies, syndromes likely affecting long-term outcome, major malformations requiring surgical correction during

newborn period, or cyanotic heart defects) are excluded for three reasons: (a) these infants are not representative of the entire population of ELBW infants, (b) it is anticipated that these conditions will strongly impair the long-term neurodevelopmental outcome in affected infants, thereby masking a moderately small effect of the treatments studied in this trial, (c) the transfusion trigger thresholds implemented are not adequate for cyanotic heart disease. The proposed study population matches the study population of the PINT study (27) with very minor modifications, thereby facilitating later meta-analyses.
To enable optimal recruitment, randomization will be performed between 48-72 hours of age after written informed parental consent was obtained and after a cranial ultrasound was performed at > 48 hours of age. By this point in time most severe intraventricular hemorrhages will have occurred and their incidence as well as their effect on long-term neurocognitive development will most likely not be affected by the RBCT guidelines. Infants with severe intra-/periventricular hemorrhage will not be excluded because (a) the differentiation of grade 2 and 3 hemorrhage and the differentiation of periventricular edema and periventricular bleeding is not always obvious, and (b) the results of the study will be more representative of the entire population of ELBW infants. However, a pre-defined subgroup analysis will be performed in infants without IVH>2° according to central reading of the 48-72 hours ultrasound. This design will avoid the critiques brought forward against a previous randomized trial (23) detailed above.

5.3.2. Sample Size and Power Calculation

The required sample size was calculated for the hypothesis underlying research question

a) "Do 'liberal' versus 'restrictive' RBCT improve or impair long-term outcome (i.e., reduce or increase the incidence of death or major neurodevelopmental impairment evaluated at 24 months corrected age) in extremely low birth weight infants?"

based on the data recently reported for PINT participants (29):

Calculations were based on a X^2-test assuming a power of 80%, a two-sided significance level of 5%, an incidence of death or major neurodevelopmental impairment (where cognitive delay is defined as MDI<85) of 128/208 (61%) vs. 109/213 (51%) in the restrictive threshold and the liberal threshold group respectively (29). Based on these assumptions, 390 patients are required in each arm (i.e., 390 patients with 'restrictive' RBCT guidelines and 390 with 'liberal' RBCT guidelines) to detect this absolute risk reduction of 10 percentage points.

5.3.3. No Interim Analysis

An interim analysis of the primary outcome measure is not planned because recruitment will be completed before the first results of long-term follow-up become available.

5.3.4. Compliance / Rate of loss to follow up

Based on our recently completed study of long-term follow-up at 5.5 years corrected age in very low birth weight infants (44; 45), we conservatively assume a 15% loss to follow-up rate for NICU survivors until 24 months corrected age. Consequently, approximately 920 have to be enrolled into the trial to ascertain the primary outcome in 780 patients.

5.3.5. Feasibility of Recruitment

As described in detail above, a total of 920 patients have to be enrolled into the trial to ensure that the primary outcome will be ascertained in at least 780 patients.

Based on our previous multicenter trials in similar populations (46-48), we very conservatively assume that approximately 65% of infants who meet the birth weight criteria and consequently will be screened for eligibility will finally be enrolled into the trial and randomized. Consequently, to enroll 920 infants, a total of 1410 preterm infants need to be screened.

For comparison: the PINT study recruited 451 (65%) of 694 eligible infants (27), and achieved ascertainment of the primary composite outcome in 430 (95%) of 451 enrolled infants (29).

Based on the numbers of infants born in the 28 participating centers, which have already committed themselves to participate, it is obvious that ~1800 infants will be screened within a recruitment period of 24 months, and based on the estimated number of infants enrolled into the trial per year provided by the local principal investigators up to 1266 patients could be enrolled in 24 months (i.e., the anticipated recruitment period is less than 24 months).

The numbers of eligible / likely recruitable infants per trial site are listed in the appendix according to the data provided by the local investigators in their declaration of commitment.

Trial sites were chosen from the large level III (German level I, i.e. "Kliniken der Maximalversorgung") neonatal units (most of them University based), which have participated previously in multicenter randomized trial such as the European erythropoietin trials, the PHELBI-trial, the NEuroSIS, or the NINSAPP-trial.

Participation of many sites in ongoing multicenter trials (PHELBI, NINSAPP, NEuroSIS, …) required the delay of the onset of recruitment to mid 2011. Earlier start is possible (and desired), if these ongoing trials complete their recruitment earlier.

5.3.6. Screening Log and Documentation of Non-Recruitment

All patients fulfilling the inclusion criteria will be documented in a 'Screening-Log'. Patients fulfilling the inclusion criteria who are not randomized are documented along with the reason for non-recruitment.

The number of patients fulfilling the inclusion criteria and not fulfilling any exclusion criterion who are not randomized should not exceed 50% of all eligible patients treated in each center.

5.3.7. Removal of Patients from Therapy or Follow-up

The local principal investigator and his local team of neonatologists are responsible for adequate treatment according to the study protocol.

As will be described in detail below (4.6.1. Investigational Medical Procedure), exceptions from the transfusion guidelines are permitted in case of pre-defined and unforeseen emergencies. The patient will be maintained in the study and the outcome will be included in the intention-to-treat analysis.

The local principal investigator may decide to take a patient of the trial if the patient meets important exclusion criteria which he has not been aware of at the time of enrolment. I.e., if a cyanotic heart defect requiring higher transfusion trigger thresholds to maintain systemic oxygen transport is detected after randomization, the patient is taken of the study transfusion guideline and is transfused at the discretion of the attending Neonatologist / Cardiologist. In that case the patient is followed according to protocol and the data is evaluated according to the intention-to-treat principle.
Relevant other medical events may lead to the decision to withdraw a patient from the study. Premature withdrawal of a patient from the study should be based on the mutual agreement between the steering committee and the local principal investigator.
All reasons for withdrawal/drop-out of a patient will be documented.

Regardless of the underlying reasons, parents have the right to withdraw their infant from the study at any time (see 5.2.8. below).

5.3.8. Premature Withdrawal of a Patient from the Study by the Parents

If parents withdraw consent, i.e., withdraw their infant prematurely from the trial, this will terminate further trial participation and consequently will terminate further application of the investigational RBCT-guidelines. The patient will be offered adequate medical treatment as appropriate without disadvantage from withdrawal from the study.
According to German legislation, the data collected so far will be entered in the database and analyzed according to the intention-to-treat principle. The parents are informed about this fact and have to give consent to this fact on the ICF.
The infant is offered the same follow-up examination as the study infants and if consent of the parents is obtained, the results will also be analysed according to the intention-to-treat principle.

5.3.9. Closure of Study Centers

The steering committee may decide to exclude participating centers of further participation on the basis of fraud or non-compliance with the study protocol or International guidelines for GCP of the participating center or the local investigator, respectively. This decision can only be taken after consulting the IDMC.

Study centers or investigators may stop recruiting patients to this study when the investigator finds inclusion of patients into this trial no more ethical for medical or organizational reasons. In this case, the local principal investigator has to give detailed reasons to the steering committee and IDMC. Both steering committee and IDMC have to

decide in this case whether to close the study center and whether to prematurely terminate the whole study.

5.3.10. Premature Termination of the Study / Stopping Rules

Premature termination of the study should always be based on the mutual agreement between steering committee and IDMC.

5.3.10.1. Premature Termination of the Study for Safety

The trial will be stopped by the coordinating investigator, on the advice of the IDMC if the risk-benefit ratio of the intervention (i.e., of either transfusion threshold) is significantly changed based on new published data becoming available definitely proofing the superiority of either intervention so that the pursuit of the trail may harm the patients.

Any complication occurring during the care of an infant enrolled in this trial will have to be reported to the coordinating investigator and the IDMC, who will continuously keep track of the incidence of such events in both study groups. (Please refer to the Pharmacovigilance Manual for details on classification of adverse events and for the time frame acceptable for reporting of adverse events.)

The coordinating investigator will stop the trial on advice of the IDMC at any time before complete recruitment of the patients in case major complications (i.e., major diseases of prematurity such as PDA requiring treatment, ROP, BPD, NEC, intestinal perforation, and brain injury on cranial ultrasound) or complications directly related to the intervention (i.e., transfusion-related adverse events) occur more frequently in one of the treatment groups.

The IDMC may also give advice in the interest of health to modify or terminate the study at any time before complete recruitment of patients if unacceptably high rates of suspected unexpected serious adverse reactions or of any serious adverse events are noted in any treatment group. (Please refer to the IDMC-Manual for details.)

5.3.10.2. Premature Termination of the Study for Unfeasibility

Unfeasibility for successful termination of the study (poor recruitment) may lead to premature termination by the coordinating investigator after consulting the steering committee and the IDMC.

5.3.10.3. Premature Termination of the Study for Efficacy

An interim analysis of efficacy is not intended because the first follow-up data will become available after the end of the recruitment period of 24 months. Premature termination for efficacy is therefore not possible.

5.4. Treatments

5.4.1. Investigational Medical Procedures

The investigational intervention is the implementation of 'restrictive' and 'liberal' (i.e., 'non-restrictive') RBCT guidelines.
Patients will be randomly allocated at day 3 of life to one of two parallel groups, i.e., to be treated according to 'restrictive' RBCT guidelines or according to 'liberal' RBCT guidelines. These guidelines will be implemented from randomization until discharge home, i.e., exclusively during the initial hospitalization.

The following thresholds for RBCT will be applied:

	Restrictive RBCT Thresholds		***Liberal RBCT Thresholds***	
state of health	'critical' venous hct	'non-critical' venous hct	'critical' venous hct	'non-critical' venous hct
before randomization:	<41%	<35%	<41%	<35%
after randomization:				
4-7 days of age	<34%	<28%	<41%	<35%
8-21 days of age	<30%	<24%	<37%	<31%
>21 days of age	<27%	<21%	<34%	<28%

Where a '*critical* state of health' is defined as the presence of any of the following:

- requirement of mechanical ventilation (any mode, excluding CPAP)
- requirement of CPAP with FiO_2>0,25 for >12h per 24h
- PDA requiring therapy
- more than 6 apnea that require stimulation per 24h or more than 4 desaturations to SpO_2<60% per 24h despite methylxanthines and CPAP
- acute sepsis or acute NEC requiring inotropic or vasopressor support

Arterial hematocrit values will also be accepted as transfusion triggers, however capillary hematocrit values will not be accepted.

Centers may choose to use trigger thresholds of haemoglobin concentrations (Hb) rather than of hematocrit values (as listed above). The respective Hb-trigger thresholds are derived by dividing the hematocrit value by 3. These Hb-triggers have then to be used throughout the study.

All RBCT are administered at doses of 12 ml / kg body weight of packed red blood cells (hematocrit 100%), i.e., 20ml/kg of a red blood cell unit with an hematocrit of 60%, over 6 hours (49).

Exceptions from these guidelines are permitted (but are not obligatory) in case of acute massive pulmonary, gastrointestinal, or any other major hemorrhage (estimated blood loss > 10% of the infants blood volume), unexplained lactic acidosis (arterial lactate > 4mmol/l), major surgery, and unforeseen emergencies but all theses exceptions have to be reported to and discussed with the coordinating investigator to reduce contamination.

5.4.2. Justification of Investigational and Control Intervention

In the absence of other established and universally accepted indicators for the need of transfusion (such as peripheral oxygen extraction (53; 54) or concentrations of VEGF (30)) these guidelines are based on venous hematocrit levels. *Hematocrit* has been chosen instead of hemoglobin to avoid confusion based on different units used for hemoglobin (i.e., g/dl vs. g/l vs. mmol/l). *Venous* hematocrit was chosen as indicator, because capillary hematocrits differ significantly from venous hematocrits in neonates (55) and only venous hematocrits have been shown to correlate with red blood cell volume (56). In contrast to capillary hematocrit values which will not be accepted, arterial hematocrit values will also be accepted as transfusions triggers.

The selected transfusion thresholds were chosen taking into account a) the range of current medical practice, b) pathophysiological considerations, c) the experience from adult and pediatric intensive care trials, and d) avoidance of criticism of previous trials and facilitation of later patient data metanalyses.

a) The range of current medical practice

Both, the restrictive and the liberal RBCT guidelines which will be compared in this trial reflect current clinical practice and represent a condensation of previously published recommendations (4; 6; 22; 50-52; 88).
The restrictive RBCT thresholds are essentially the same as in the PINT study (27), thereby facilitating later meta-analyses. It appears that there is currently consensus that RBCT guidelines for preterm infants should not be more restrictive at present (59).
Furthermore, up-to-date, yet unpublished data of an international survey on transfusion trigger thresholds which was coordinated by Haresh Kirpalani (University of Pennsylvania) to which the Coordinating Investigator contributed proves that the chosen transfusion guidelines reflect current practice. This data will be presented for the first time at the Jahrestagung der Gesellschaft für Neonatologie und Pädiatrische Intensivmedizin in Mannheim on May 28th 2011.
966 Neonatologists (including 109 Neonatologist from Germany) participated in this survey. The Neonatologists were asked for their hemoglobin trigger threshold in infants with a birth weight < 1000g, in several scenarios with different postnatal ages and different degrees of respiratory support. The following graphs exemplarily show the responses to four of these scenarios and include the transfusion thresholds that will be applied during ETTNO (left arrow: restrictive guidelines; right arrow: liberal guidelines) – based on a conversion from hematocrit [%] to hemoglobin [g/dl] by division by 3.

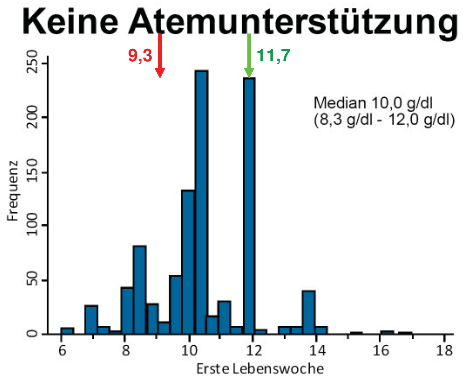

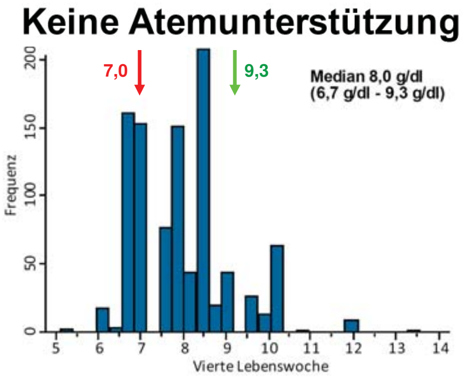

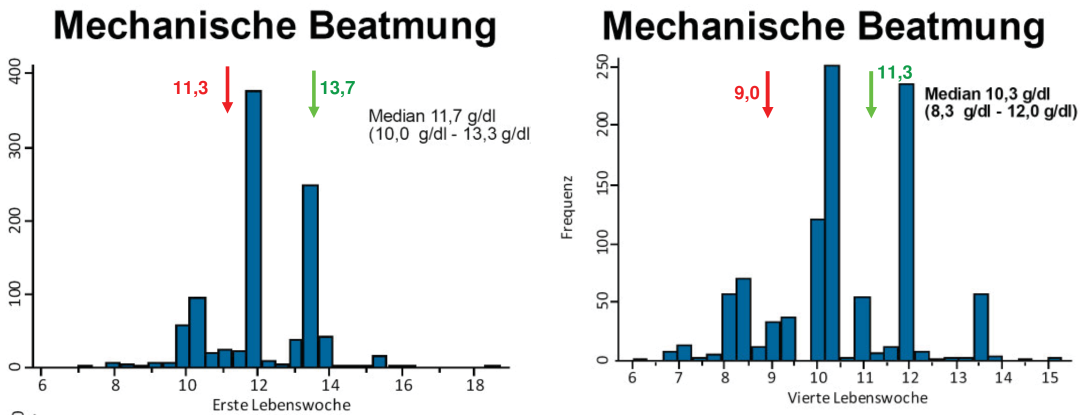

It is obvious from the graphs that the chosen transfusion thresholds 1) represent current practice, 2) are placed on either side of the median transfusion threshold reported in the survey, and 3) by far do not reflect the extremes of transfusion thresholds reported.
It is also obvious from the graphs that transfusions thresholds vary greatly. This enormous variation can best be explained by the lack of evidence (i.e., the existing equipoise) on which transfusion threshold are most appropriate for preterm infants, highlighting the urgent need for the ETTNO trial.
Finally, the chosen transfusion thresholds are within the ranges suggested by a very recent expert summary of the available evidence (88).

b) Pathophysiological considerations
In contrast to trials in adult and pediatric intensive care, several transfusion threshold were chosen for ETTNO in either treatment arm depending 1) on postnatal age and 2) on cardiorespiratory status.
Based on the modified and simplified Fick equation:

$$VO2 = CO * Hb * (SaO2 – SvO2) * k$$

where VO2 is oxygen consumption, CO is cardiac output, Hb is hemoglobin concentration, and SaO2 and SvO2 are arterial and mixed venous oxygen saturation, low hemoglobin levels can be compensated by an increased cardiac output or an increased arterial-venous oxygen extraction. This indeed occurs in preterm infants as demonstrated by Bell et al. (89) and Alkalay et al. (90).
Lower transfusion trigger thresholds with increasing postnatal age were chosen, taking into account that cardiac adaptation to anemia by increase in heart rate is somewhat more limited in neonates than in older patients because of the higher baseline heart rate and by increase in stroke volume and cardiac output requires time, i.e., increasing postnatal age.
And higher transfusion thresholds with more critical cardiorespiratory status of the child were chosen to take into account that systemic perfusion (the fraction of the cardiac output that does not shunt through a patent duct) and arterial oxygen saturation and hence the ability to compensate for anemia may be compromised because of cardiorespiratory disease. Or, as the Iowa group puts it: “It is not known, however, whether the most critically ill preterm infants – whom we did not study – have this same adaptive capability“ [as the healthy ones studied in their recent report on physiological effects of packed red blood cell transfusion in preterm infants] (89).
Both, variation of transfusion threshold with postnatal age and with cardiorespiratory status again reflect current practice (compare references (4; 6; 22; 50-52)) – and can also be

seen in the above depicted graphs and the following graph summarizing answers to the above described international survey:

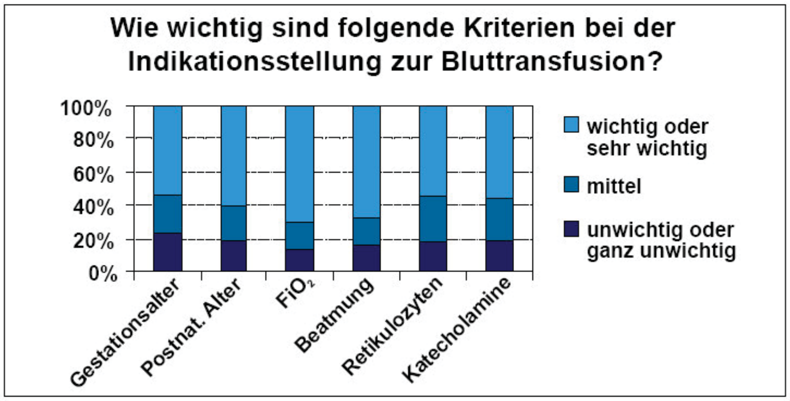

c) The experience from adult and pediatric intensive care trials

The restrictive RBCT thresholds reflect the observation from pediatric and adult intensive care that accepting hemoglobin concentrations as low as 7g/dl (i.e., a hematocrit of 21%) are safe or even beneficial in stable patients (57; 58).

d) Avoidance of criticism of previous trials and facilitation of patient data metanalyses

Care has been taken that these guidelines will result in a clinically relevant difference in mean hemoglobin concentrations between both treatment groups. For each clinical situation, the difference in the hematocrit threshold between restrictive and liberal RBCT guidelines is 7%, i.e., a difference in mean hemoglobin concentrations of about 2.3g/dl can be expected between the treatment groups. Consequently, the liberal RBCT guidelines are "more liberal" than those applied in the PINT study and the expected difference in mean hemoglobin concentrations is substantially larger than the 1g/dl observed in the PINT study (27). Aiming for this larger difference between the treatment groups takes into account the criticism brought forward against the PINT trial (28) and will improve recognition of differing outcomes and differing risks for complications if those exist. Furthermore, an individual patient data meta-analysis (which we intend to perform at the conclusion of our trial) will be able to elucidate whether the 'more' liberal RBCT guidelines applied in this trial will have different effects compared with the 'less' liberal RBCT guidelines of the PINT study because both will be compared with similar restrictive guidelines.

The lack of conclusive data on the effect of transfusion thresholds on neurocognitive development has been described in detail in **section 3 The Medical Problem**.

5.4.3. The Investigational Medical Product and its Manufacturer

For RBCT only standard ABO- and Rh-compatible, leukocyte-depleted and (in most instances) irradiated red blood cell concentrates will be administered within the marketing authorization ("Zulassung"). Exemplarily, a summary of product characteristics ("Fach- und Gebrauchsinformation") is provided in the appendix to the protocol for the irradiated red blood cell concentrate administered at the University childrens Hospital of Tuebingen.

These standard red blood cell concentrates will be provided by the local blood bank of each study center and have been produced and labeled by the local blood bank or the local manufacturer according to their production standard approved by the Paul Ehrlich Institute will be administered.

The list of the local blood banks and the appropriate authorization codes of the regulatory authority ("Zulassungs-Nummern des Paul-Ehrlich-Instituts") of the red blood cell concentrates is provided in the appendix.
Photocopies of the "Herstellungserlaubnis" of these red blood cell concentrates are available upon request.

5.4.4. Preparation, Labelling, and Administration of the Investigational Medical Product

The standard red blood cell concentrates will be manufactured and labeled by the local supplier of blood products according to procedures previously approved by the Paul Ehrlich Institute.

The local blood bank of each center will select and cross-match each red blood cell concentrate according to German legislation and guidelines, i.e.,:
a) according to National law ("Transfusionsgesetz") (60)
b) according to National guidelines ("Richtlinien der Bundesärztekammer zur Gewinnung von Blut und Blutbestandteilen und zur Anwendung von Blutprodukten (Hämotherapie)" (61) and „Querschnitts-Leitlinie zur Therapie mit Blutkomponenten und Plasmaderivaten" (62)).
c) according to local Standard Operating Procedures where appropriate, e.g., for the University Hospital of Tuebingen according to the Dienstanweisung zur „Vorbereitung und Durchführung von Bluttransfusionen am Universitätsklinikum Tübingen" (63).

There won't be any study-specific label on the red blood cell concentrates, because these blood products are not in any way different from standard blood products, do not differ between the study groups, and are administered exclusively within the marketing authorization (i.e., the "Zulassung"). The only investigational procedure is the different hematocrit threshold triggering the indication for the RBCT.

The administration also strictly follows the above referenced laws and guidelines, with the only exception that the transfusion triggers (i.e., the investigational intervention in this study described above under heading 5.4.1.) differ to some degree from the non-evidence-based transfusion triggers outlined in the "Querschnitts-Leitlinie zur Therapie mit Blutkomponenten und Plasmaderivaten" (62). The reasons for this exception are explained in detail under heading 1. „The Medical Problem" and under heading 5.4.2. "Justification of Investigational and Control Intervention".

The local investigator and his team are responsible for:
- Receiving parental consent for administration of blood products
- Adequate control of compatibility of the blood unit delivered and adequate bedside testing to verify compatibility and exclude incorrect administration
- Documentation in the chart of the indication for the RBCT, the blood unit and the amount administered, and timing of the administration, the bedside test and its result

- Adequate monitoring of the patient's well being during the transfusion

5.4.5. Storage, Distribution, and Return of the Investigational Medical Product

The standard red blood cell concentrates will be stored, distributed, and returned according to the National and local guidelines referenced above (60-62).

5.5. Methods Against Bias

5.5.1 Methods of Assigning Patients to Treatment Groups / Randomization

On day three of life (between 48 and 72 hours of life), randomization will be performed using consecutively numbered, sealed, opaque envelopes, which have been produced using a computer generated randomization sequence (using the software „Randlist Version 1.2“ purchased from DatInf GmbH Tübingen). The envelopes are filled and verified by personal not involved in the conduct of the study at the Center for Pediatric Clinical Trials at the University Children's Hospital of Tuebingen, and the random sequence lists are also not accessible for personal involved in the conduct of the study.
The randomization will be stratified for center and birth weight groups (400-749g / 750 – 999g). A variable block size will be applied.
Every patient will be assigned a unique identification number for pseudo-anonymous data entry and analysis.

5.5.2. Blinding

This is an observer-blinded study.
The personal performing the neurocognitive evaluation (including physical and neurological examination, anthropometric measurements, and assessments of hearing and vision) of the infants at 24 months corrected age (and - if additional funding is approved - at 6 years corrected age) as well as the personal performing the laboratory analyses of peroxidation products and VEGF will be blinded to the patient's group assignment (i.e., assignment to either the 'restrictive' or the 'liberal' RBCT guideline).

5.5.3. Further Efforts to Avoid Bias

Further efforts made to avoid bias are described in detail elsewhere but are summarized for completeness here:

a) RBCT guidelines, iron supplementation, protein and vitamin supplementation, delayed cord clamping and as far as possible blood sampling routines and principles of neonatal care will be standardized among all participating centers. The cumulative sampling blood loss will be documented in every patient (see 5.6. Concomitant Therapy).
b) In each center, all eligible infants are documented in a 'Screening-Log' along with reasons for non-participation if applicable.
c) Patient data will be entered into the study data base continuously throughout the trial using web-based, password-protected, electronic case report forms (see chapter 5.9.2. Database, Remote Data Entry, eCRF). Data entry will be monitored to ensure completeness. Queries and discrepancies will also be resolved continuously.
d) In addition to an initiation visit and a site-closure visit, on-site-visits will be performed to monitor at least 25% of case report forms (depending on the quality of the data and the completeness and the reliability of the case report forms from that center) and to check the consent forms, the in- and exclusion criteria, the primary outcome, and few key secondary outcome of all cases.
e) Study meetings with updates on recruitment and study related problems will be held at regular intervals.
f) The personnel filling the electronic case report forms and the personnel conducting the follow-up examinations including the Bayley Scales will be trained and/or accredited centrally. The reference and training manual "Surveillance of Cerebral Palsy in Europe", a video based training tool for the standardization of the diagnosis of cerebral palsy, is provided to all participating centers (64).

g) Cranial ultrasound scans will be evaluated centrally (to avoid inter-observer variability) by personnel blinded to the patients' treatment group assignment.
h) Finally, for confirmatory efficacy analysis the data of the intention-to-treat population will be used.

5.6. Prior and Concomitant Therapy

Interventions which have previously been proven efficacious for the prevention and treatment of anemia of prematurity will be standardized for all study participants by implementation of a uniform treatment guideline (SOP (standard operating procedure) Delayed Cord Clamping, SOP Iron Supplementation, SOP Protein Supplementation, and SOP Folic Acid and Vitamin B12 Supplementation detailed below) for all participating centers.

5.6.1. SOP Delayed Cord Clamping

Delayed cord clamping is recommended for all preterm infants with an anticipated birth weight < 1500g (65).
All centers participating in the study have to implement the following:

- a routine of reminding the obstetrician of the benefits of delayed cord clamping
- Recommend a delay in cord clamping for at least 30 up to 45 sec after delivery of the infant provided it is possible to hold the infant below the placenta.
- Recommend tilting of the operating table to the side of the obstetrician holding the infant in case of a cesarean section and seating of the obstetrician while holding the child.
- Recommend shielding of the infant against evaporation and heat loss by a plastic sac, a plastic wrap or a warmed blanket.
- If delayed cord clamping is not possible for any reason, milking of the umbilical cord towards the infant should be recommended.

5.6.2.SOP Iron Supplementation

Enteral iron supplementation reduces the need for blood transfusions in preterm infants (66).
All centers participating in the study have to implement the following:

- Start of iron supplementation as soon as 100 ml/kg/d of milk are tolerated at a daily dose of at least 2mg/kg/d. The dose is increased to at least 4mg/kg/d as soon as the infant tolerates 150ml/kg/d of milk feeds.
- Discharge the infants at an oral dose of at least 4mg/kg and recommend to gradually taper the dose to 2mg/kg/d throughout the first year of life.

5.6.3. SOP Protein Supplementation

Protein supplementation improves erythropoiesis (i.e, hematocrit and reticulocyte count) in preterm infants (67).
All centers participating in the study have to implement the following:

- Enteral and parenteral protein supplementation is increased until 4-4.5g/kg/d of protein intake are achieved (68; 69).
- Protein supplementation can be tapered in thriving infants to 3.5-4.0g/kg/d after reaching a weight of >1.0kg (69).
- Protein supplementation can be further tapered to 3.0-3.5g/kg/d after reaching discharge provided the infant continues to thrive (69).

5.6.4. SOP Folic Acid and Vitamin B12 Supplementation

Folic acid and Vitamin B12 supplementation improve erythropoiesis in preterm infants (70).

All centers participating in the study have to implement the following:

- Enteral and parenteral vitamin supplementation is given in a dose of about 40µg/kg/d folic acid and 0,5µg/kg/d Vitamin B12 (corresponding to 1ml/kg/d Soluvit N).

5.6.5. Reduction and Documentation of Iatrogenic Blood Loss

Reduction of iatrogenic blood loss is also effective for prevention of anemia of prematurity and impacts transfusion requirements (71). Therefore, reduction of iatrogenic blood loss will be strongly encouraged throughout the trial. Blood sampling losses will be recorded throughout the initial hospitalization.

5.6.6. No Erythropoietin

The application of erythropoietin, which is not routinely used in most German NICUs, will be prohibited during the study for the following 3 reasons: (a) administration of erythropoietin results only in a small reduction of donor exposure and is associated with ROP according to a recent systematic review (72), (b) erythropoietin will stimulate erythropoiesis and potentially increase haemoglobin levels particularly in infants randomized to restrictive transfusion thresholds. It may thereby diminish the difference in the mean haemoglobin concentrations between the treatment groups and obscure treatment effects, and (c) based on observations that erythropoietin and the erythropoietin receptor are expressed in the developing human brain and that erythropoietin has neuroprotective properties in vitro and in animal models of brain injury, erythropoietin may theoretically have neuroprotective effects in preterm infants (reviewed in (73)) and may thereby attenuate any treatment effect on neurocognitive development.

5.6.7. RBCT before Randomization

Prior to randomization, i.e., until day 2 of life, RBCT can be administered to maintain the hematocrit >40% in infants requiring mechanical ventilation and >34% in spontaneously breathing infants (not obligatory).

5.6.8. Iron Supplementation after Discharge

After discharge from the hospital, enteral iron supplementation should be continued at a minimum of 2mg/kg/d throughout the first year of life according to the recommendations of the American Academy of Pediatrics.

5.6.9. Co-interventions Proven to Affect Long-term Outcome

Further co-interventions are common in the target population of extremely low birth weight infants, who require intensive support for multiple organs. Treatments proven to positively affect long-term outcome (i.e., antenatal steroids (74), prophylactic surfactant before 30 weeks gestation (75), caffeine for apnea of prematurity (76)) will be standardized for the study. Furthermore a guideline restricting postnatal administration of dexamethasone, which is known to adversely affect neurodevelopmental outcome (77), will be implemented.

5.6.10. Oxygen Saturation Targets

Physiological considerations suggest that a given patient may tolerate lower hemoglobin levels better if the degree of oxygen saturation of hemoglobin is higher. The existing evidence on risks and benefits of higher or lower oxygen saturation targets for preterm infants is still controversial (78-81), and there is no data available that suggests that there is an interaction between the degree of anemia and the effects of oxygen saturation

targets. Therefore, heterogeneity of oxygen saturation targets between centers will be accepted during this study. The applied oxygensaturation targets in each center will be documented and checked throughout the study on monitoring visits.
Sub group analyses will be performed comparing the effects between centers with higher and lower oxygen saturation targets and patients with frequent or infrequent oxygen desaturations (according to the pulse oximetry data recorded, see 5.8.1. routine examinations).

5.6.11. Other Co-interventions

Heterogeneity between centers will be accepted for treatments of unproven long-term benefit. Although this heterogeneity of patient care may obscure a subtle treatment effect, heterogeneity of patient care (at least to a certain degree) is also considered to be a strength of multicenter trials: If a treatment effect can be observed despite heterogeneity of patient care this treatment effect is robust – and the results of the study can be generalized to other settings.
All these other treatments are therefore not regulated by the study protocol but are left to the participating centers according to current guidelines (at www.awmf-online.de and according to current evidence: http://www.nichd.nih.gov/cochrane/cochrane.cfm).

5.7. Treatment and Follow-up after Completion of the Trial

The investigational transfusion trigger thresholds will be applied until discharge home. The timing of discharge will be determined by the local attending neonatologist according to standard care guidelines of the respective center.

After discharge home, all patients will be treated with standard treatment (if any treatment is required at all) and continued care is in the responsibility of the Pediatrician of the family in cooperation with the local study center.
Follow-up is provided by the local study center according to the local standards, e.g., in Tuebingen the first visit in the Follow-up clinic is scheduled at three months corrected age and further follow-up is provided according to the needs of the patient and family.
The follow-up visit at 24 months corrected age with standardized neurological examination and neuro-developmental assessment will be performed according to German regulations (i.e., according to G-BA: "Anlage 1 zur Vereinbarung über Maßnahmen zur Qualitätssicherung der Versorgung von Früh- und Neugeborenen" vom 20. September 2005).
Further standard follow-up will be provided by the Pediatrician ("Vorsorgeuntersuchung") and additional specialized neurodevelopmental / pulmonary / cardiac / ... follow-up will be provided by the local study center if required.

5.8. Compliance with Treatment / Study Protocol and Follow-up

RBCT will be administered to preterm infants during their initial hospital stay (until discharge home), consequently patient compliance will not be a problem.

Monitoring will be performed to ensure compliance of the investigators with the protocol. Especially, in- and exclusion criteria, written informed parental consent forms, and the primary endpoint will be checked in every patient (100% source data verification). The steering committee or its designees (clinical monitors) may visit participating centers to control adherence to the protocol. A detailed Monitoring Manual is attached in the Appendix.

Measures will be taken to increase compliance with the follow-up visit as follows:

a) the parents will be reminded at discharge from hospital and the address of parents and grandparents will be noted in the patients chart,
b) the Pediatrician will be informed of the follow-up in the discharge summary,
c) the parents will be contacted by phone call or short-term follow-up visit (at the discretion of the local study center) at 6 and 18 months,
d) a birthday card will be sent with a very short questionnaire and a free return envelope and
e) a written invitation to the follow-up visit will be sent followed by phone calls to reconfirm participation.
f) Finally, home visits will be arranged for patients in whom the parents refuse follow-up in the out-patient department.

5.9. Scheduled Examinations

The treatment period of the study is completed before the preterm infants are discharged from the hospital, consequently there are no study visits during the treatment period.

There will be only one study visit after discharge form the neonatal intensive care unit at the end of the follow-up period at 24 (23-25) months corrected age for assessment of the primary outcome (see 4.1. and 4.2. “Primary Endpoint” and 5.8.1. “Routine Examinations”).

Finally, follow-up at 5.5-6 years of age is also intended and a separate protocol and fund application will be developed as soon as appropriate.

5.9.1. Routine Examinations

During the treatment period, all infants enrolled in the study are cared for in a level III neonatal intensive care unit (according to German legislation: “Level 1 Perinatalzentren”). Continuous monitoring of heart and respiratory rate and oxygen saturation will be implemented routinely as indicated. Furthermore blood pressure, blood gas, urine output and hematological and biochemical examinations will be performed at the discretion of the attending Neonatologist to guide neonatal intensive care.

Arterial hemoglobin oxygen saturation by pulse oximetry (SpO2)

- will be monitored continuously as standard of care in all study centers from birth until discharge from hospital is foreseeable, when this type of monitoring is discontinued at the discretion of the attending Neonatologist.
- oxygen saturation targets are chosen and implemented according to the standard operating procedure of the neonatal intensive care unit / at the discretion of the attending Neonatologist.

Study-driven deviation from standard care (ancillary Study A):

- a study pulse oximeter is connected to the patient and by a “sat-share-cable” hooked into the central monitoring system. The internal memory of the pulse oximeter is set to store a reading every 2 sec (follow instructions for “trend configuration” provided with the pulse oximeter). The alarm of the study pulse oximeters is turned off – and alarm settings (according to the local NICU SOP) are implemented at the central monitoring device.
- the trend data (i.e., the internal memory of the study pulse oximeter) is retrieved once a week (follow instructions for “data download” provided with the pulse oximeter) via RS232-to-USB-cable to a local computer, and heart rate, oxygen saturation, perfusion index and pleth variability index data (stored at 2 sec intervals) are sent by e-mail as data file (txt-file) in a pseudoanonymized fashion labeled only with the patient’s ETTNO-study number to

PD Dr. med. Axel Franz
University of Tuebingen
Dept. of Pediatrics, Neonatology
Calwerstraße 7
72076 Tübingen
telephone: +49-7071-29-0 (ask to page Dr. Franz at #2322)
fax: +49-7071-29-3969
e-mail: axel.franz@med.uni-tuebingen.de

Whole blood counts:

- will be determined at the discretion of the attending Neonatologist.

Study-driven deviation from standard care:

- for study purposes whole blood counts will always be determined with reticulocytes and reticulocyte hemoglobin content (this will not require any additional blood volume).
- these clinically indicated whole blood counts will be documented for the study to proof compliance with the RBCT guidelines.

Blood gas analyses before and after blood transfusions:

- will be determined at the discretion of the attending Neonatologist.

Study-driven deviation from standard care (ancillary study D):

- if available these clinically indicated blood gas analyses will be documented for the study for the evaluation of disturbances of gas exchange associated with TRALI.

Ultrasound examination of the brain:

- these assessments are standard of care and are completely pain free.

Study-driven deviation from standard care:

- for better comparability these examinations will be performed at four pre-determined time points: i.e., at 48-72h (for baseline description before randomization), at 7 (±2) and 28 (±4) days of life, and at less than 2 weeks before discharge (i.e., at 34-37 weeks postmenstrual age).
- these cranial ultrasound examinations are recorded as loops in the coronal plain from front to back and in the sagital/para-sagital plain from right to left. The loops will be stored as avi-files on DVDs and sent for central re-evaluation by personnel who is blinded to the patients' treatment group assignment.
- DVDs labeled with ETTNO-study number and the date of the examination are sent by standard mail to:

PD Dr. med. Axel Franz
University of Tuebingen
Dept. of Pediatrics, Neonatology
Calwerstraße 7
72076 Tübingen
telephone: +49-7071-29-0 (ask to page Dr. Franz at #2322)
fax: +49-7071-29-3969
e-mail: axel.franz@med.uni-tuebingen.de

Clinical, neurocognitive, psychomotor and anthropometric assessment at follow-up:

- these assessments are standard of care at 24 months in Germany and according to current regulations (Gemeinsamer Bundesausschuss: "Anlage 1 zur Vereinbarung über Maßnahmen zur Qualitätssicherung der Versorgung von Früh- und Neugeborenen" vom 20. September 2005) all perinatal centers are obliged to ensure this assessment in all infants with a birth weight < 1500g.
- determination of the primary outcome is therefore not associated with additional burden to the infants enrolled in the study or their families.

Study-driven deviation from standard care:

- for the study, the assessment will be strictly formalized (as detailed above under 3.2. Determination of primary outcome) and the training of the personal who will perform this assessment will be centralized. The local principal investigators have to ensure that the personal will be blinded for the patient's group assignment.

5.9.2. Study-Driven Examinations

For surveillance of the treatment and for evaluation of the secondary study endpoints, the following blood, saliva and urine samples are scheduled throughout the initial hospital admissions. All blood samples are taken during clinically indicated venipuncture/arterial blood sampling procedures or are scheduled in time windows that will allow sampling along with blood test that are clinically indicated to guide intensive care of these high risk infants **(i.e., there will not be any additional venipuncture / capillary blood sampling caused by the study!)**.

Participation in this part of the study is optional for the participating study centers.

5.9.2.1. Study-Driven Blood samples

The following blood samples are scheduled just prior to and after RBCT that have been ordered by the treatment team according to the study guidelines. The samples will be taken together with the bedside test which is required before RBCT anyway (i.e., there won't be any additional venipuncture caused by the study).

The blood loss caused by these samples will be replaced immediately by adding 0,5ml to the scheduled RBCT (i.e., if 20ml blood were to be administered in a 1kg child, than 20,5ml will be administered instead to replace the study-related blood loss immediately). Consequently, parents can be assured that no additional RBCT will ever be required because of blood sampling for the study.

Furthermore, the purely study related blood samples are only 0,2ml of whole blood. In comparison with an average *daily* iatrogenic blood loss of 0,67ml/kg throughout the hospital stay documented in one of our previous multicenter trials (47) this amount of study associated additional blood loss would be negligible – if it was not replaced anyway (as described above).

Pre-transfusion blood sample: (200µl EDTA-blood) for determination of VEGF before transfusion (ancillary study B):

Aliquots of 100µl EDTA plasma of the pre-transfusion sample are labeled with the ETTNO study number and the date of blood sampling. The samples are then frozen at -20 and shipped (after prior notice by telephone call) in batches on dry ice by overnight express delivery for determination of VEGF (using a commercially available enzyme-linked immunosorbent assay (RD Systems, Minneapolis, MN) to :

Prof. Dr. med. Mario Rüdiger
University of Dresden
Dept. of Pediatrics, Neonatology
Fetscherstraße 74
01307 Dresden
telephone: +49-351-458-3640
fax: +49-351-458-5358
e-mail: mario.ruediger@uniklinikum-dresden.de

5.9.2.1. Study-Driven Urine samples

Urine samples (1ml) will be collected at day of life 3 (72-96 hours of life) and day of life 7 (120-168 hours of life), and thereafter weekly until 36 weeks of postmenstrual age or until discharge (whichever is first). In many centers, clinically indicated urine samples are collected anyway weekly in preterm infants to guide Calcium and Phosphorous supplementation.
Urine collection will only be performed non-invasively, unless the infant requires catheterization for other indications.

Urine samples should be marked with the ETTNO-study number and the date of sampling. Thereafter, samples have to be centrifuged (10min at 3000rpm) and the supernatants have to be divided into 3 aliquots and deep frozen at -80℃ until analysis.
Frozen urine samples are sent in batches on dry ice by overnight express delivery to

A) the Laboratory for Eicosanoids and Mass-Spectrometry (Dr. rer. nat. Horst Schweer) at the Department of General Pediatrics at the University of Marburg (Department Head Prof. Dr. Maier).

Dr. rer. nat. Horst Schweer
University of Marburg
Neonatology and Neuropediatrics
AG Instr. Analytik
Baldingerstraße
35043 Marburg
telephone: 49-6421-28-66229
fax: 49-6421-28-68970
e-mail: horst.schweer@staff.uni-marburg.de

Dr. rer. nat. Schweer will perform the analyses for Malondialdehyde, 3-Nitrotyrosine, 4-Hydroxynonenal, 8-Hydroxy-2'-desoxyGuanosin, and 8-Isoprostane by gas-(or liquid-)-chromatography – tandem mass-spectrometry.

and
B) and C) to

PD Dr. med. Axel Franz
University of Tuebingen
Dept. of Pediatrics, Neonatology
Calwerstraße 7
72076 Tübingen
telephone: +49-7071-29-0 (ask to page Dr. Franz at #2322)
fax: +49-7071-29-3969
e-mail: axel.franz@med.uni-tuebingen.de

Dr. Axel Franz will arrange for determination of the Kreatinin concentration for normalization of all measurements to the Kreatinin concentration.

5.9.2.2. Study-Driven Measurements of Cerebral Oxygenation

A limited number of centers in which the cerebral oxymeter is available will participate in ancillary study B2. Based on data from Klüpfer et al (University of Leipzig) (31), cerebral and peripheral oxygenation is measured with an INVOS 5100 oxygenation monitor (Somanetics, Boulder, CO USA) which has a CE certificate (CE0197) and a neonatal use marketing authorization. The measurement technology is similar to the technology of standard pulse oximetry and is non-invasive and pain-free. Soft sensors are placed on the forehead (to measure cerebral oxygenation) and the lumbar region (to measure renal/visceral oxygenation. The sensors contain light emitting diodes (LED) emitting infra-red light and two optodes for detection of light after tissue penetration.

Measurements of cerebral oxygenation will be performed at:

- 2-4 hours before the transfusion is started (i.e., after the indication for RBCT became apparent and before the blood was delivered from the blood bank.
- while the packed red blood cells are being infused
- 1 h after the transfusion is finished
- 2-4 hours at 24 ± 6 hours after the transfusion ended

Placement and removal of the sensor will be done when the patient is approached for routine care anyway, and the patients sleep will not be disturbed to perform the measurements.

Values for cerebral and peripheral oxygenation are stored in the internal memory of the device and downloaded to a standard computer after the measurements were completed. The downloaded data is sent by e-mail as data file (txt-file) in a pseudo-anonymized fashion labeled only with the patient’s ETTNO-study number and the date of transfusion to:

Prof. Dr. med. Ulrich Thome
University of Leipzig
Dept. of Pediatrics, Neonatology
Liebigstraße 20a
04103 Leipzig
telephone: +49-341-97-26021
fax: +49-341-97-23579
e-mail: ulrich.thome@medizin.uni-leipzig.de

5.10. Documentation

5.10.1. Documentation of Raw Data

All raw data, including laboratory results are considered to be source data and must be documented in the patient's clinical file (case record) and retained in a secure place and made available for review during routine monitoring or audits or on request of the Steering Committee.
The local principal investigator must document in the patient's clinical file that the patient participated in this study. Furthermore a small sign attached at the patient's bed or incubator will inform about the participation in the study and the transfusion trigger threshold to be applied.

The original of the signed informed consent form, the checklist of in- and exclusion criteria have to be retained in the Investigator Site File (ISF, part II: patient data) along with photocopies of the primary and key secondary outcome data (i.e., neurodevelopmental assessment form at follow-up, reports of ophthalmological examinations, chronic lung disease evaluation form).
A photocopy of the signed informed consent form has to be entered into the patient's clinical file and another photocopy of the signed informed consent form has to be given given to the parents / legal guardian of the patient.

Case records (patient's clinical files) and all study relevant material (Investigator Site File) are to be retained for at least 10 years following the end of the study at the local study site.

Beyond the need of documentation for this study, in Germany, the physician in charge of the patient and her/his institution have to document a) the information of the parents with regard to the transfusion, b) the consent of the parents to administer the transfusion to their child, c) the result of the blood group analysis, and d) the effect and any undesirable side effect for at least 15 years, and patient identification (name, first name, date of birth, address), the identification of the transfused erythrocyte concentrate, the administered dose (i.e., the transfused volume) and the day and time of the administration for at least 30 years, according to German legislation (Transfusionsgesetz, §14, (60)). Likewise, study centers outside Germany have to strictly follow their national legislation in this respect.

5.10.2. Database, Remote Data Entry, eCRF

A web-based electronic case report form (eCRF) for encrypted and password protected remote data entry will be used for documentation.

Data is entered into this database exclusively in a pseudo-anonymized form (see below: section 6.8.: Confidentiality / Data Protection).

The database (Koordobas, see http://www.koordobas.de) is validated and fulfils the requirements of ICH-GCP and FDA 21 CFR Part 11.

The person responsible for the database is :

Dr. med. Christoph Meisner
University of Tuebingen

Institute for Medical Biometry
Westbahnhofstr. 55
72070 Tübingen
telephone: +49-7071-29-78253
fax: +49-7071-29-5075
e-mail: christoph.meisner@med.uni-tuebingen.de

The eCRF includes all necessary forms, separated according to the anticipated investigation time-points.
The local principal investigator is responsible for the quality and correct entry of all data documented in the CRF, i.e., the identity of the entered data with the data in the patient's clinical file. All the information on which the entries in the CRF are based must be available in the patient's clinical files, e. g., results of laboratory investigations.

Only the local principal investigator or qualified personnel authorized by him/her are entitled to enter data into the CRF.
All authorized personnel must attend a formal training before get access to the eCRF only after the training is successfully completed.
Each database user has his or her access authorization. It is strictly prohibited to pass the access authorization to any other person. Each database user is responsible for the database entries under his account.

For each database entry the following information is logged:

- User ID
- Time of data entry
- In case of data modification: new and previous value of the modified dtata and the reason for performing modification.

Corrections and additions must be entered into the eCRF by qualified study personnel and can be followed based on the integrated audit trail of the database.

Any remaining questions or missing data will be noted on data clarification forms (Queries, discrepancy forms (DCF)), which will be sent to the local principal investigator. It is the local principal investigator's obligation to complete and return them to the data management as soon as possible.

Case records (the patients' clinical files) and all study relevant material (Investigator Site File) are to be retained for at least 10 years following the end of the study at the local study site. The local principal investigator will ensure that a correct assignment of the CRFs to the corresponding patient's clinical files is possible at any time.
A separate patient identification list must be recorded by the local principal investigator. Patients ID-lists and the patients' clinical files (case records) will be kept separately at the individual study sites by the local principal investigator. The patient ID-List is kept along with the original signed parent consent form in the Investigator Site file.
One photocopy of the signed consent form has to be placed in the patient's clinical file (case record of the patient) and another one has to be returned to the parents / legal guardian.

After prior agreement, a check of the consistency of data between the patient files and the eCRF as well as an inspection of all other documents related to the study (Investigator Site File) can be conducted by the responsible authorities and/or by monitors (inspection/audit/monitoring).

After termination of the study, the local principal investigator will enter all relevant information obtained subsequent to the study in the patient's medical records.

5.11. Data Management

When a patient is enrolled into the study, a checklist of in- and exclusion criteria has to be filled in and stored in the Investigator Site File (part II: patient data)a long with the original written parental consent form.

Missing, questionable, or additional information will be requested on a regular base by the data management. The data management will remind the local principal investigator for missing documentation regularly.

During entry, data will be checked by constraints and triggers. In case of gaps in the data, the responsible center will be asked to supply the missing data. The database will be checked for faults and validated by the database programmer. Complete and incremental data backup will be performed regularly.

All essential study documents will be archived for at least 10 years. Patients ID-lists and medical records will be kept separately at the individual study sites.

The person responsible for data management is:

Dr. Corinna Engel
Center for Paediatric Clinical Studies
Biometry
Frondsbergstr. 23
72076 Tübingen
telephone: +49-7071-29-89111
fax: +49-7071-29-NN
e-mail: corinna.engel@med.uni-tuebingen.de

5.12. Data Quality Assurance / Monitoring

All institutions have previous experience with the conduct of clinical trials in accordance to the Declaration of Helsinki and with Good Clinical practice.
Curricula vitae, descriptions of individual study experience, GCP training, and conflict of interest statements of each investigator (Prüfarzt) are available upon request and attached as annex 7 to the submission to the ethics committees involved ("Angaben zur Qualifikation der Prüfstelle, Lebensläufe und Angaben zur Studienerfahrung der Prüfärzte und deren Stellvertreter, Conflict of Interest Statements der Prüfärzte und deren Stellvertreter (nach Prüfstellen geordnet)").

5.12.1. Monitoring

In order to guarantee a high quality of the study and data retrieval, all participating centers will be visited on a regular base on site by clinical monitors. Data protection rights will be respected.

The trial will be monitored by the "Center for Pediatric Clinical Studies" at the Universitätsklinikum Tübingen. Monitoring will be done according to ICH-GCP guidelines and performed according to Standard Operating Procedures and a monitoring manual (see appendix) specifies all aspects of monitoring including check lists for the initiation visits and routine monitoring visits.

The person responsible for monitoring is:

Dr. Joachim Riethmüller
Center for Pediatric Clinical Studies
University of Tuebingen
Dept. of Pediatrics,
Hoppe-Seyler-Str.1
72076 Tübingen
telephone: +49-7071-29-81391
fax: +49-7071-29-4450
e-mail: joachim.riethmueller@med.uni-tuebingen.de

Before a center begins to recruit patients a "pre-study" initiation visit will be performed to ensure availability and completeness of all study material, approval of the local ethics committee, adequate teaching of local doctors and nurses. Reports of pre-study visits will be provided to the coordinating investigator and the funding agency.

Furthermore, monitoring to control original data and to verify accurate data registration and management will be performed at each center at 3 months after initiation of the center and thereafter at 6-12 monthly intervals. Monitoring will be tailored to the importance of the data. The following data will be submitted to 100% source data verification: existence of the patient, patient identification number, informed consent, correct interpretation of inclusion and exclusion criteria, serious adverse events (timely reporting and follow-up), and primary and key secondary outcome data. All other patient data will be submitted to 10% source data verification and patient files will be sampled at a random basis. The rate of monitoring is increased if a high error rate is detected.

The monitor will also have regular contact by phone and/or e-mail with all participating centers to control the study progression, adherence to the study protocol, and to discuss

problems related to the study. He/she will particularly concentrate on adverse events, the number of drop-outs, and excluded patients. The local principal investigators in the participating centers will support the monitor in his/her activities.

At the end of the study and after completion of the follow up, a study closure visit will be performed at each center.

5.12.2. Compliance with the Protocol

CRFs and the central data base will be continuously assessed with regard to protocol violations. Transfusions which were not triggered by the assigned thresholds must also be reported separately and will be monitored by the Data Monitoring Committee. If a performance problem is detected, advice and / or additional training of study personal will be provided at the respective study site.

6. Ethical and Legal Aspects

6.1. Ethical Considerations

Knowledge about the effects of RBCT thresholds on neurocognitive development is crucial, because RBCT are widely implemented despite the lack of conclusive data on long-term consequences. RBCT guidelines became more restrictive and lower hemoglobin levels are accepted these days in preterm infants (51), however these treatment decisions are not evidence-based. Both, the restrictive and the liberal RBCT guidelines which will be compared in this trial reflect current clinical practice (4; 22; 51; 52). Continued treatment of thousands of premature infants in ignorance of what are safe and effective hemoglobin levels seems to be unethical.

To prevent that insufficient power of the trial may result in an unreliable or incorrect interpretation of the results of this trial, the sample size was determined to achieve a power of 80% to detect an absolute risk reduction by 10 percentage points of the important long-term composite outcome “death or major neurodevelopmental impairment”. The design of the study was adjusted to facilitate later pooling of the data with the data of the PINT study to facilitate later meta-analyses.

Furthermore, the proposed large multicenter trial will allow to study adverse events potentially related to RBCT or anemia.

Most importantly, the research question underlying this clinical trial can not be answered by studying animal or adult humans, but can only be answered by a study in the population concerned: premature infants at risk of both, frequent blood transfusions for anemia of prematurity and impaired neurodevelopmental outcome. The results of the trial may help to improve long-term outcome of premature infants and to reduce society’s health care costs.

6.2. Risk / Benefit Consideration for the Individual Participant and Potential Additional Burden Caused by Participation in the Clinical Trial

The well-being of all study infants will be closely monitored and the care will not differ from other patients in the NICU.
In particular, participation in the study is not associated with any additional pain (e.g., no additional venipunctures, capillary blood samples etc., because study-driven blood sampling will occur at the time of clinically indicated blood samples) or additional physical examinations.

- Study-driven blood loss is directly associated with the need of RBCT, blood will be sampled at the time of clinically indicated blood sampling, and study-driven blood loss will directly be compensated by a small additionally transfused volume of red cell concentrate at the time of RBCT. The volume of study-driven blood loss is negligible compared with the volume of blood loss caused during routine neonatal intensive care.
- Study-driven urine sampling will be non-invasively only.
- Monitoring by pulse oximetry (also used for ancillary study A) will be performed according to the standard operating procedures of the local NICU – and there will only be a study-driven read-out of the memory of the pulse oximetry device.

- Monitoring of cerebral oxygenation at the time of RBCT only requires temporary positioning of a small, soft sensor – similar to that used for standard pulse oximetry - on the forehead and the lumbar region of the child. Placement and removal of the sensor will be done when the patient is approached for routine care anyway, and the patients sleep will not be disturbed to perform the measurements.

Finally, data suggests that sick newborn infants may benefit from participation in a randomized controlled trial (82).

In summary, the trial is associated with a minimal extra burden for the infants enrolled. Considering the possible implications of the study results for the care of future infants, the conduct of the trial seems ethical – if not mandatory.

6.2.1. Risks and Benefits of Liberal versus Restrictive Transfusion Thresholds

For the individual participant, the risks and potential benefits are as follows:
'Liberal' RBCT guidelines may be associated with an increased risk of ROP (12-14), and BPD (15; 16). Although in theory any transfusion carries an extremely small risk of transmitting viral diseases (11) and hence increasing the number of transfusion applying 'liberal' guidelines will increase this risk, transfusion-related viral infections remain extremely unlikely and probably irrelevant given the high risk of other morbidities in this extremely preterm population. After RBCT, the load of free iron may be increased (83), potentially resulting in an increased load of reactive oxygen molecules (induced by the Fenton reaction) and an increased risk of diseases thought to be related to such molecules (15; 16). 'Liberal' RBCT guidelines may have the benefit of improved oxygen transport to all organs.
'Restrictive' RBCT guidelines in contrast carry the theoretical risk of at least temporarily insufficient oxygen transport to organs, especially the brain, and impaired outcome. 'Restrictive' RBCT guidelines will decrease the risk of transfusion-transmitted viral disease and may potentially be associated with a lower incidence of ROP, BPD, and other complications of prematurity thought to be associated with an increased load of reactive oxygen molecules.

To protect study participants, RBCT are possible throughout the trial at the discretion of the attending Neonatologist in the case of both pre-defined and unforeseen emergencies. The attending Neonatologist assesses the need for transfusion (and all other intensive care interventions) continuously based on vital signs routinely monitored during neonatal intensive care.

6.2.2. Risks and Benefits of Longterm Follow-Up

In patients who develop normally without long-term sequelae of prematurity, the process and the results of the neurocognitive, motor and neurological evaluation at 24 months corrected age may be reassuring for the patients and their families. In patients with long-term handicaps after preterm birth, both examination and confrontation with the poor results may be a burden. Nevertheless, these patients are also likely to profit from the long-term follow-up because developmental delays may be identified that may not have been addressed and consequently may not have been treated before. The long-term follow-up implemented by the study may also improve the awareness for the need of a developmental follow-up in the families and the care-taking pediatricians.

6.3. Ethics Committee (EC)

The final study protocol, including the final version of the written informed consent form, must be approved or given a favourable opinion by the ethics committee (EC) responsible for the principal coordinating investigator before commencement of the study.

The ethics committee (EC) responsible for the principal coordinating investigator:

Ethik-Kommission der Medizinischen Fakultät
Herrn Prof. Dr. med D. Luft (Vorsitzender)
Gartenstraße 47
72047 Tübingen

The final study protocol, including the final version of the written informed consent form will also be provided to all local ECs. The list of the local ECs is provided in the Appendix.

The principal coordinating investigator is responsible for informing his EC of any amendment to the protocol.

Local modifications of the protocol are not permitted.

The principal co-ordinating investigator must also provide his EC with any reports of suspected unexpected adverse reactions (by expedited reporting) and any serious adverse events (within the annual safety report) from the study.

6.4. Ethical Conduct of the Study

This study will be conducted following international guidelines for Good Clinical Practice (ICH-GCP) and the Helsinki Declaration. Particular aspects are the study protocol, patient information sheet, informed consent form, submission to EC, administrative documents, data registration, registration of adverse events, preparation for inspection and internal audit by authorised personal or the IDMC, and storage of study documents.
The patient will not pay any costs attributed to the type of procedure or investigation medication.

6.5. Regulatory Authority Approvals/Authorizations/Registration

The national regulatory authority, (here the Paul Ehrlich Institut (PEI)), will be asked for approval of the trial in accordance with European and national legislation.

All local regulatory authorities (Regierungspräsidien) will be notified of the trial after the approval of the PEI and the positive opinion of the ethics committees were obtained. The list of the responsible local regulatory authorities (Regierungspräsidien) is provided in the appendix.

The EUDRACT number (see page 1) was be assigned in the process of application for approval by the PEI.

The study will be registered in the Current Controlled Trials Register (http://www.controlled-trials.com/).

6.6. Subject Information (here: Parents Information) and Consent (here: Parental Consent)

Before inclusion (i. e. before randomization or any other study specific procedure is undertaken), the parents or legal guardians of participating patients need to give written informed consent after full and adequate oral and written information about the nature, purpose, possible risks and benefits of the study.

To obtain informed consent, the parents of patients eligible for the study will be approached at the first or second day of life when the attending neonatologist feels that the situation is appropriate (parents must be able to freely understand and question, extremely vulnerable and / or underage parents will not be approached, if necessary, the neonatologist will be accompanied by an interpreter).

The following information will be provided: explanation of the purposes of the study, entry criteria, expected duration of participation, description of the different procedures including randomization, potential risks and benefits, the impact of the study for the participant and for future patients, the voluntary status of participation, protection of subjects and their data, and contact information.
Parents are informed that refusal to participate will not result in any penalty or disadvantage, and that they are free to withdraw consent, i.e., withdraw their infant from the study, at any time without any disadvantages and without need to give any reason for their decision.
The patients need to be reassured that good clinical practice will be maintained throughout the trial.
The parents will also be informed on and have to agree with data registration and data monitoring and inspections by qualified personal of the principal coordinating investigator or regulatory authorities.

The parents of every participating patient will receive a copy of the parent information and a copy of the signed informed consent form.

Within each centre the local principal investigator will be responsible for obtaining consent. He can delegate the information of the parents to a qualified physician who is informed in full detail about the study. The parents will be given the opportunity to ask questions and allowed time to consider the information provided. The German version of the parent information and the informed consent form will be attached as appendix.

The original of the written informed consent will be kept with the study documentation at the individual study site (Investigator Site File, part: patient data). In addition, a copy of the written informed consent will be archived with the case records. During monitoring, informed consent forms will be checked for each participating patient.

Parent information will be updated if new relevant information gets available changing the risk-benefit assessment. Parents of patients already enrolled into the study will be informed by the local principal investigator especially if patient's safety is concerned.

Parent information and informed consent form need approval by the ethics committee. Local modifications are not permitted.

6.7. Insurance

According to German and European regulations clinical trial insurance will cover compensation to subject(s) for trial-related injury.

The insurance will be provided by:

ECCLESIA mildenberger HOSPITAL GmbH
Klingenbergstraße 4
32758 Detmold

Parents will be informed about the insurance and their obligations with respect to the insurance coverage in the parent information.

6.8. Confidentiality / Data Protection

The written informed consent form will explain that the study data will be stored in a pseudo-anonymized form in a central study database, maintaining confidentiality in accordance with local data legislation and the EU directive 95/46/EG.

The access to this information will be encrypted and password protected and only the physicians and study nurses directly involved in the study will have access.
Subjects in this database will be identified by unique patient identifier (study code) only. The following patient identifiers will be removed for pseudo-anonymization: Name or initials, address, postal code, hospital number, and insurance number. This information will be replaced with a unique identifier identifier.
Only a separate patient identification list, which is securely held by the local principal investigators (in the Investigator Site File, part: patient data) - separately from the patient case records - will enable patient identification.

The parent information will also explain that for data verification purposes, authorized representatives of the Universitätsklinikum Tübingen (the sponsor), the regulatory authorities, the relevant ethics committee, or an institutional review board may require direct access to parts of the medical records relevant to the study, including the subjects' medical history. Only infants whose parents or guardians consent to these inspections according to current legislation will be enrolled into this study.

Specifically, pseudo-anonymity of the patients will be guaranteed when presenting the data at scientific meetings or publishing them in scientific journals.

6.9. Conflict of Interest

None of the investigators has a conflict of interest to declare. This is documented in the annex 6 of the material submitted to the ethics committee ("Angaben zur Qualifikation der Prüfstelle, Lebensläufe und Angaben zur Studienerfahrung der Prüfärzte und deren Stellvertreter, Conflict of Interest Statements der Prüfärzte und deren Stellvertreter (nach Prüfstellen geordnet)")

6.10. Financial Compensation for Study Participants

A Financial Compensation for Study Participants is not intended.

7. Safety

7.1. Independent Data Monitoring Committee (IDMC)

An independent data monitoring committee (IDMC) (consisting of 3 independent experts: 2 pediatricians, 1 biostatistician not involved in this trial and listed below (section 7.3. Members of the IDMC) will be implemented to assess the progress of the clinical trial, the issue of contamination (i.e., transfusions not triggered by the assigned threshold) and cumulative safety data for evidence of treatment harm and benefit.

The IDMC will meet or convene by telephone conference after 100, 300, 500, and 700 patients randomized into the study were discharged from the hospital.

The IDMC may give advice to modify or terminate the trial at any time before complete recruitment of patients if (a) new data become available that suggest that the risk/benefit ratio for the patients is significantly changed and the pursuit of the trail may harm the patients, or (b) successful termination of the study becomes unfeasible because of poor recruitment.

Most importantly, the IDMC will compare the rates of adverse events (i.e., the incidence of ROP, BPD, NEC, intestinal perforation, and brain injury on cranial ultrasound, and the frequency of treatment for PDA, and unforeseen emergencies) in both treatment groups. The IDMC may give advice to modify or terminate the trial if these analyses show higher rates of adverse events in any of the treatment groups.

The IDMC is not responsible to assess critical efficacy endpoints because first measurements of these endpoints will be performed when recruitment and study treatment have been terminated.

7.2. Safety Analyses

Safety analyses will be conducted four times: after 100, 300, 500 and 700 patients have reached the end of their therapy phase (i.e., at discharge home).

The safety analyses will be conducted using coded group labels A and B blinded for the real therapy groups. On request, the IDMC will also be provided with the true group assignment.

The primary parameter for assessing safety is the incidence of major events. A major event is defined as the occurrence of any one of the following events during the period from the beginning up to the end of the study therapy (i.e, from randomization until discharge home):

(1) Death for any reason

(2) Adverse treatment effects

- PDA treated with Indomethacin, Ibuprofen and/or surgical ligation
- NEC or intestinal perforation
- ROP >2
- ROP requiring treatment
- BPD at 36 weeks GA
- Brain injury on cranial ultrasound at 48-72h, 7d and 28d of life
- Nosocomial infection

(3) any (other) serious adverse event (SAE) (see below section 9)

Secondary parameters for assessing safety are the incidences of all individual components of these major events (i.e, the incidences of all single major complication of prematurity (i.e., PDA requiring therapy, necrotizing enterocolitis (NEC), intestinal perforation, ROP, BPD, brain injury on cranial ultrasound, and nosocomial infections)).

Safety analyses will comprise calculation of counts and percentages for adverse events. Adverse event rates will be compared by chi-square test or Fisher's exact test between treatment groups and 95% confidence intervals for the differences in the rates between the treatment groups will be provided.
The safety report will comprise the rate of occurrence of any major event as detailed above as well as the individual rates of these events (i.e., the incidence of ROP, BPD, NEC, intestinal perforation, and brain injury on cranial ultrasound, and the frequency of treatment for PDA, and unforeseen emergencies) .

The results will be reported directly to the members of the IDMC.

Based on these results the IDMC will decide whether or not to recommend to the Steering Committee (SC) to stop the study.

Additionally safety analyses demanded by the Bundesoberbehörde (Paul-Ehrlich Institut Berlin) will be done according to their requirements.

The safety analyses will be performed and reported to the IDMC by:

Dr. Corinna Engel
Center for Paediatric Clinical Studies
Biometry
Frondsbergstr. 23
72076 Tübingen
telephone:+49-7071-29-89111
fax: +49-7071-29-NN
e-mail: corinna.engel@med.uni-tuebingen.de

7.3. Members of the IDMC

Neonatologists:

Prof. Dr. med. Heike Rabe
Consultant Neonatologist, Lead for Research
Brighton and Sussex University Hospitals NHS Trust
Trevor Mann Baby Unit
Royal Sussex County Hospital
Eastern Road
Brighton, BN2 5BE
UK
phone +44-1273-696955 ext 4195/4296
fax: +44-1273-664435
e-mail: Heike.Rabe@bsuh.nhs.uk

Prof. Dr. med. H. U. Bucher
Klinikdirektor
Frauenklinikstrasse 10
CH - 8091 Zürich
Tel: +41 1 255 53 40
FAX: +41 1 255 44 42
e-mail: buh@fhk.usz.ch

Statistician:

Prof. Dr. Josef Högel
University of Ulm
Dept. of Human Genetics
89070 Ulm
telephone:49-731-500- 65458
fax: 49-731-500-NN
e-mail: josef.hoegel@uni-ulm.de

8. Statistical Methods

8.1. Statistical and Analytical Plans

The study has one main objective:
To test the hypothesis, that 'liberal' RBCT practices that intend to keep the hematocrit levels ≥ 28% at all time during the initial hospitalization will improve or impair long-term outcome (i.e., reduce or increase the incidence of death or major neurodevelopmental impairment evaluated at 24 months corrected age) in extremely low birth weight infants if compared with 'restrictive' RBCT guidelines that accept hematocrit levels as low as 21% (according to the RBCT guidelines described in detail above).

All statistical tests will be two-sided at a significance level of 5%. Descriptive statistics will be provided and will include counts and percentages for categorical variables and mean, standard deviation, minimum, first quartile, median, third quartile, maximum for quantitative variables.

8.1.1. Study Populations

Patients fulfilling all inclusion criteria (i.e., all patients admitted during the recruitment period with a birth weight of 400-999g) should be listed in the study center's screening log. Patients with a birth weight of 400-999g but with an exclusion criterion will be documented as screening failures. These patients may not be randomized but the reason for screening failure has to be documented. The rate of screening failures per center should not exceed 50%.

The following populations will be formed:

1. **Screening population:** consists of all screened patients and will be used just to describe the relation between screened and randomized patients and reasons for non-randomization.
2. **Safety population:** consists of all randomized patients, treated according to either investigational transfusion trigger threshold and will be the basis for all safety analysis.
3. **Intention-to-treat (ITT) population:** consists of all randomized patients. Patients who changed therapy and patients who were not treated according to protocol for any reason will be analyzed in the group they have been randomized.
4. **Modified intention-to-treat (mITT) population:** consists of all randomized patients in whom the primary outcome was ascertained. Patients who changed therapy and patients who were not treated according to protocol for any reason will be analyzed in the group they have been randomized.
5. **Per-protocol (PP) population:** consists of all patients of the ITT population with the exception of those patients, who received any transfusion outside the assigned threshold indications including transfusions administered during surgery, and those patients, who were not transfused despite having hematocrit values below the trigger thresholds. In the unlikely event that a patients changed therapy immediately after randomization, this patient will be analyzed in the group he/she has been treated.

A full analysis of all data will be done for the ITT-population. For the PP-population the primary endpoint and the MDI-score will be analyzed.

The analysis of the primary outcome variable in the modified intention-to-treat population of all randomized subjects in whom the primary outcome was ascertained will be considered confirmatory. Other analyses are considered explorative.

8.1.2. Description of the Study Population

All relevant demographic data and baseline characteristics will be analyzed descriptively by treatment group in the mITT population. Demographic data and baseline characteristics of the ITT population will also be analyzed for the interim report after all patients have been discharged from hospital.
For categorical data chi-square tests will be used to compare treatment groups, continuous data will be compared with ANOVA or Wilcoxon tests.

8.1.3. Primary Endpoint

The analysis of the primary outcome variable in the modified intention-to-treat population of all randomized subjects in whom the primary outcome was ascertained will be considered confirmatory.

The primary outcome variable (binary) will be analyzed descriptively by treatment group and by logistic regression with factors treatment, centre and birth weight category at a two-sided significance level of 5% to test the null hypothesis of equal proportions in the two treatment groups.

For the MDI score (as part of the primary outcome variable) in the intention-to-treat analysis, missing values (of children who are so severely handicapped that they can not be tested) will be imputed by a pre-defined scheme:
Children whose severe cognitive impairment or disability precludes the use of the Bayley Scales of Infant Development will be assigned a MDI score of 30 if minimal speech and the ability for minimal communication with the parents are present, and a MDI score of 20 if no speech is present but at least minimal sensory or motor achievements are elicited
For children lost to follow-up, a score will be imputed based on details obtained from the Pediatrician caring for the infant: severely retarded infants whom the Pediatrician rates as non-testable will be imputed by 30 if minimal speech and the ability for minimal communication with the parents are present, and by 20 if no speech is present but at least minimal sensory or motor achievements are elicited. Scores of 50 – 80 will be allocated according to a-priori determined criteria in the assessment of the Pediatrician.

The composite primary outcome will be considered present if 1 or more of the individual components of the composite outcome are known to be present, or absent if all components are known to be absent. If no component was present, 1 or more missing components will cause the primary outcome to be deemed missing.

A worst case scenario analysis in the intention-to-treat population will be performed following imputation of “death or neurodevelopmental impairment” for all infants in whom the primary outcome was not ascertained (i.e., including those infants lost to follow-up). The results will be included in the final report to enable an assessment of the possible impact of missing data.

Additionally the same analyses will be performed in the per-protocol population. In the per-protocol population no strategies for imputing missing values will be applied.

8.1.4. Secondary Endpoints

All analyses of secondary endpoints will be considered explorative in the intention-to-treat population, the modified intention-to-treat population and the per-protocol population.
Because the treatment group assignment is not blinded during the initial treatment phase (i.e., until discharge from hospital), secondary outcomes (i.e., neonatal outcomes) will be reported in the ITT population after discharge of the last patient, before results for the primary outcome become available.

Secondary outcome variables will be analyzed descriptively by treatment group. They will be compared between the treatment groups by logistic regression with factors treatment, center and birth weight category if binary and by ANOVA or ANOVA on ranks with factors treatment, center and birth weight category if quantitative.
Assumption of normal distribution will be visually inspected. In case of departures from normality appropriate transformations may be used or non-parametric tests will be applied.

For secondary outcome variables no imputation strategies will be applied for missing values, because a) death is major secondary outcome and will specifically reported in every analysis, and b) an interim report will summarize all neonatal outcomes (including death) in the ITT-population of all randomized infants at the time of discharge of the last patient before follow-up (i.e., before potential loss of patients).

8.1.5. Subgroup Analyses

The following subgroups will be formed from the ITT-population:

(1) infants with birth weights 400-749g and 750-999g
(2) male and female infants
(3) infants from centers with ‘higher’ and ‘lower’ oxygen saturation targets (where centers with ‘lower’ oxygen saturation targets are defined by a central value of the target oxygen saturation range of less than the median of the central values of the target oxygen saturation ranges of all centers).

Only the primary endpoint, the incidence of cerebral palsy, and the MDI-Score will be analyzed in these subgroups (by logistic regression with factors treatment, center and birth weight category (for primary endpoint and the incidence of cerebral palsy) and by ANOVA or ANOVA on ranks with factors treatment, center and birth weight category (for the MDI score)).

8.1.6. Analyses of Ancillary Studies

Analyses of ancillary studies will be performed in the intention-to-treat populations from the participating centers based on observed data.

Ancillary Study A:

The analysis will be performed in the ITT population.
Infants will be subdivided into 4 categories according to quartiles of
a) total duration of intermittent hypoxemic episodes (hemoglobin-oxygen-desaturations to less than 60% / 80% SpO2),
b) number of intermittent hypoxemic episodes,
c) total duration of bradycardia to less than 60/min / less than 80/min, and
d) number of bradycardias to less than 60/min / less than 80/min.

e) total duration with a blood oxygen content of less than 6.5ml/dl (Hb x SpO2 x 1.34 = 8g/dl x 60% x 1.34ml/g = 6.5 mlO2/dl), 8.0ml/dl, and 9,6ml/dl.

The incidence of the primary outcome variable, the incidence of an MDI score <85 and <70, and the incidence of cerebral palsy will be determined for each category and displayed graphically.
Risk factors for poor neurodevelopmental outcome will be determined using multiple logistic regression with variable selection selecting important risk factors from established risk factors (gestational age <26weeks, male gender, IVH>2°, …) and potential markers of intermittent poor oxygen transport.

Ancillary study B:
VEGF in plasma and urine will be compared between responders and non-responders to RBCT (according to pre-defined changes in heart rate, frequency of desaturations, weight gain, daily intake by bottle feeding (incontrast to NG-tube feeding), and serum lactate) in order to assess the relevance of VEGF as predictor for the need for RBCT in transfused infants. To analyze the diagnostic value of VEGF for predicting a clinical response to RBCT, an optimal cut-off value for VEGF will be determined using ROC-curve analysis, and sensitivity, specificity and positive and negative predictive values will be determined for this cut-off value. Combinations of hematocrit and VEGF values will also be evaluated.

Cerebral oxygen saturation measured by near infrared spectroscopy will be compared between responders and non-responders to RBCT (according to pre-defined changes in heart rate, frequency of desaturations, weight gain, daily intake by bottle feeding (in contrast to NG-tube feeding), and serum lactate) in order to assess the relevance of cerebral oxygen saturation measured by near infrared spectroscopy as predictor for the need for RBCT in transfused infants. An optimal cut-off value for cerebral oxygen saturation as a parameter to predict a clinical response to RBCT will be determined using ROC analysis, and sensitivity, specificity and positive and negative predictive values will be determined for this cut-off value. Combinations of hematocrit and cerebral oxygen saturation values will also be evaluated.

Ancillary Study C:
Urine concentrations of Malondialdehyde, 3-Nitrotyrosine, 2-Hydroxynonenal und 8-Isoprostane of weekly urine samples will be compared between treatment groups in the ITT population using descriptive and graphical methods.
Risk factors for diseases of prematurity for which reactive oxygen species are thought to play a pathophysiological role (i.e., retinopathy >1°, chronic lung disease, white matter injury defined herein as ventricular dilatation) and for an MDI score < 85 will be determined using multiple logistic regression with variable selection selecting important risk factors from established risk factors (gestational age <26weeks and male gender) and from the areas under the concentration curves of the peroxidation products.

Ancillary study D:
Furthermore, the incidence of transfusion related acute lung injury will be estimated in the study population based on an expected number of up to 4500 RBCT (5 RBCT / patient (27)).

8.1.7. No Interim Analysis for Efficacy

An interim analysis for efficacy (i.e., for the primary endpoint) will not be performed, because recruitment will be terminated before follow-up data will be available. However, an interim analysis of secondary outcomes (i.e., neonatal outcomes) will be reported in the ITT population after discharge of the last patient, before results for the primary outcome become available.

Until recruitment of the last patient, the IDMC will monitor the incidence rates of serious adverse events and specific adverse incidents. Analysis of these data will be performed by an independent statistician not involved in this trial (member of the IDMC).

8.1.8. Responsible Biostatistician

Prof. Dr. Martina Kron
University of Ulm
Dept. of Biometry and Medical Documentation
89070 Ulm
telephone: 49-731-500-26904
fax: 49-731-500-26902
e-mail: martina.kron@uni-ulm.de

9. Adverse Events

9.1. Warnings/Precautions

The investigational medical intervention ('restrictive' and 'liberal' RBCT guidelines) are currently used in neonatal centers and there is no evidence that any of these treatments is more or less beneficial or harmful with regard to long-term outcome of very and extremely low birth weight infants. As described in detail in the introduction, there is epidemiological evidence that reducing RBCT may be beneficial – but the concept of a minimal hemoglobin concentration required to provide adequate oxygen transport at all times has not been disproven yet, especially not in infants with apnea and bradycardia of prematurity.

The most important adverse outcome with any of the treatment arms would be an unacceptably high rate of diseases or complications of prematurity (chronic lung disease (defined as oxygen saturation as measured by pulse oximetry of less than 85% at room air at a postmenstrual age of 36 weeks), retinopathy of prematurity (>2°), necrotizing enterocolitis (≥ 2° according to the Bell's criteria)).

To ensure optimal protection of the study participants, monitoring for adverse events, management of these events and reporting of these events are mandatory. The procedure of what to monitor for, what to report, to whom to report and how to report adverse events is described herein and also in the Pharmacovigilance Manual (in the appendix).

Compliance with this protocol will enable meaningful continuous safety analyses performed by the IDMC (see IDMC manual in the appendix).

9.2. Adverse Event Definitions according to ICH GCP

9.2.1. Adverse Event

An adverse event (AE) is any unfavourable and unintended sign (including an abnormal laboratory finding, for example), symptom, or disease in a subject. This does not imply that there is a relationship between the adverse event and the intervention under investigation.

9.2.2. Serious Adverse Event / Definition of Seriousness

Events that pose a threat to a patient's life or functioning are considered "serious".
A serious adverse event (SAE) is any untoward medical occurrence or effect that at any dose

- results in death,
- is life-threatening,
    - NOTE: Life-threatening in the definition of a serious adverse event or serious adverse reaction refers to an event in which the subject was at risk of death at the time of event; it does not refer to an event which hypothetically might have caused death if it were more severe
- requires hospitalisation or prolongation of existing inpatients' hospitalisation,
- results in persistent or significant disability or incapacity,
- is a congenital anomaly or birth defect

Should an AE be considered serious it must be additionally documented and reported on a separate SAE report form.

Medical and scientific judgement should be exercised in deciding whether expedited reporting is appropriate

9.2.3. Expected Adverse Events

RBCT carry the small risk of a) blood transmitted diseases (HIV, Hepatitis B and C, CMV, and others) and b) transfusion reactions (herein defined as any of the following temperature instability, tachycardia (>180/min), arterial hypotension requiring intervention, deterioration of gas exchange, or rash in immediate timely association with a RBCT). Furthermore, death and diseases or complications of prematurity (chronic lung disease, retinopathy of prematurity, necrotizing enterocolitis, PDA requiring treatment, nosocomial infections, intra- or periventricular hemorrhage, PVL, ventriculomegaly), although not related to the study interventions in most infants have to be screened for as expected AEs, because the rate of occurrence may theoretically be affected by the treatment. All the AEs listed in this paragraph have to be considered serious.

As common SAEs (>10%) must be expected:

- death
- nosocomial infections (including blood culture positive sepsis, clinical sepsis, and pneumonia according to the NEOKISS definition)
- chronic lung disease of prematurity
- patent ductus arteriosus requiring therapy
- intraventricular hemorrhage
- periventricular hemorrhage / infarction
- ventriculomegaly
- retinopathy of prematurity >2°

Death after randomization (i.e., after day 4 of life) and diseases of prematurity – although probably not related to the study in most infants have to be classified as rare SAEs (5-10%):

- necrotizing enterocolitis > 2°
- intestinal perforation

All other expected adverse events have to be classified as very rare SAEs (<5%):

- periventricular leukomalacia
- porencephalic cyst
- blood transmitted infection
- transfusion reaction

9.2.4. Unexpected Adverse Event

An adverse reaction, the nature, or severity of which is not consistent with those listed in section 9.2.3. is regarded as unexpected.

9.2.5. Relationship of Adverse Event to Investigational Medical Intervention (here: RBCT according to either RBCT guideline)

For each reported adverse event, the local principal investigator must make an assessment of the relationship of the event to the (in this study) RBCT.
The relationship to the investigational therapy should be assessed using the following definitions:

- **Certain (i.e., the AE is definitely related)**: A clinical event, including laboratory test abnormality, occurring in a plausible time relationship to the RBCT, which cannot be explained by concurrent disease or other drugs or chemicals. The response to

withdrawal of the drug (dechallenge) should be clinically plausible. The event must be definitive pharmacologically or phenomenologically, using a satisfactory rechallenge procedure if necessary.

- **Probable (i.e., the AE is probably related)**: A clinical event, including laboratory test abnormality, with a reasonable time sequence to the RBCT, unlikely to be attributed to concurrent disease or other drugs or chemicals, and which follows a clinically reasonable response to withdrawal (dechallenge). Rechallenge information is not required to fulfil this definition.
- **Possible (i.e., the AE is possibly related)**: A clinical event, including laboratory test abnormality, with a reasonable time sequence to the RBCT, but which could also be explained by concurrent disease or other drugs or chemicals. Information on drug withdrawal may be lacking or unclear.
- **Unlikely (i.e., the AE is probably not related)**: A clinical event, including laboratory test abnormality, with a temporal relationship to the RBCT, which makes a causal relationship improbable, and in which other drugs, chemicals or underlying disease provide plausible explanations.
- **Not assessed**: A clinical event, including laboratory test abnormality, reported as an adverse reaction, which was not judged at the time of reporting, because e.g. more data is essential for a proper assessment or the additional data are under examination.
- **Unassessable**: A report suggesting an adverse reaction which cannot be judged because information is insufficient or contradictory, and which cannot be supplemented or verified.

9.2.6. Adverse Reaction

An Adverse Reaction (AR) of an investigational medical intervention (here RBCT according to either RBCT guideline) is any untoward and unintended response to this investigational medical intervention. The phrase 'reaction/response to a (here) medical intervention' means that a causal relationship between the medical intervention and the adverse event is at least a reasonable possibility, i.e., a causal relationship cannot be ruled out.

In the context of the present study all AE listed in section 9.2.3. will be reported and evaluated as AR, because a causal relationship – although unlikely - cannot be ruled out.

9.2.7. Suspected Expected Serious Adverse Reactions (SESARs)

In the context of the present study all AE listed in section 9.2.3. will be reported and evaluated as SESAR, because a) they fulfil the criteria of seriousness and b) a causal relationship – although unlikely - cannot be ruled out.

9.2.8. Suspected Unexpected Serious Adverse Reactions (SUSARs)

A suspected unexpected serious adverse reaction (SUSAR) is any adverse reaction, which is not consistent with the nature or severity with those listed in section 9.2.3., fulfilling the criteria of seriousness (section 9.2.2.).

9.3. Management of Adverse Events

Adverse events should be managed with appropriate diagnostic work-up and causal and supportive treatment, ensuring that the source of harm is removed.
After stabilization of the patients condition, documentation (section 9.4.) and reporting (9.5.) are the next steps.

9.4. Documentation of Adverse Events

All AEs will be documented in the CRF. AEs will be estimated to be “serious” or “non-serious”. These categories define the documentation and reporting of the corresponding adverse event.

The AEs will be recorded on the appropriate CRF page, including:

- date of onset and date of resolution,
- severity,
- relationship to study drug,
- serious or non-serious,
- and discontinuation of study medication.

Each adverse event will be followed until resolution or through the last day of the study and the post-treatment follow-up visit.
Pre-existing diseases present prior to administration of study medication, will be documented as concomitant diseases as part of patient history in the CRF. Any disease newly occurring or increasing in severity during the course of the study will be documented as an AE.

All the SAE (common, rare, and very rare) listed under heading 9.2.3. (Expected Adverse Events) will be documented in the CRF, entered in the database, and regularly evaluated by the IDMC.
Characterisation of these SAEs listed under 9.2.3. **(with the exception of death and blood culture positive sepsis)** ***is not required because all these foreseeable events are severe, serious, a relationship to the RBCT guidelines can not be excluded (but is unlikely), and the RBCT guidelines should not be abrogated as a consequence of these events.***
Furthermore, because of the fact that these AEs (listed under 9.2.3.) in the vast majority of instances are not related to the investigational medical intervention (i.e., the implementation of liberal versus restrictive RBCT guidelines), these AEs will not be reported separately (in addition to the documentation in the CRF) – **again with the exception of death and blood culture positive sepsis, which both require additional separate reporting and characterisation (with regard to the potential relationship to the transfusion guidelines) as described below.**
Justification:
Although according to ICH-GCP SAEs and SESARs should be reported within 1 day to the principal coordinating investigator and the sponsor this is not possible and not desirable in this high risk study population. According to a recent similar study in a similar population

(birth weight < 1000g) (27) the following rates of the expected SAE / SESARs have to be anticipated:

- death	20%
- blood culture positive sepsis	40%
- chronic lung disease of prematurity	55%
- periventricular hemorrhage / infarction	10%
- retinopathy of prematurity >2°	20%
- intestinal perforation	10%
- necrotizing enterocolitis > 2°	5%
- periventricular leukomalacia	3%
- ventriculomegaly	13%

Consequently, the local study teams and the coordinating study team would have to process more than 1600 SAE / SESAR reports in the 920 patients enrolled, most of which would not be related to the investigational medical intervention but would have to be judged well-known complications of extreme prematurity. This can not be achieved given the limited public funding – and is not desirable because in this surge of SAE reports (which are most likely unrelated to the investigational intervention) important SUSARs may easily be overlooked.

Procedure for DEATH and BLOOD CULTURE POSITIVE SEPSIS:
In addition to documentation in the appropriate forms of the CRFs all deaths and all events of blood culture positive sepsis have to be reported and characterized both on the AE CRF and also on the SAE report form and immediately report to the principal coordinating investigator as described in section 9.5.

9.5. Reporting of Serious Adverse Events

Every SAE, regardless of causal relationship, occurring in the course of the study has to be documented in the CRF.
The local principal investigator will inform the principal coordinating investigator as soon as possible, latest within 24 hours about any SAE (except those listed under 9.2.3. as justified in section 9.4. above).
All SAEs (except those listed under 9.2.3 – but including deaths and cases of blood culture positive sepsis) will be evaluated by the principal coordinating investigator whether they fulfil the criteria of seriousness and expectedness. The principal coordinating investigator will notify the IDMC about all such SAEs.
The IDMC may re-evaluate cases and judge whether or not such an event has to be classified as SUSAR.
The principal coordinating investigator will inform his ethic committee, the competent authority (PEI) and all investigators about any relevant changes of the risk benefit ratio.

All suspected adverse reactions related to an investigational medicinal product/intervention (i.e., the transfusion trigger thresholds) which occur in this trial, and which are both unexpected and serious (SUSARs) are subject to expedited reporting.
The principal coordinating investigator informs all investigators concerned of relevant information about SUSARs that could adversely affect the safety of the patients.

9.5.1. Notification of the Ethics Committee and Competent Authority

Any suspected unexpected serious adverse reaction will be reported to the competent authority (PEI) and the responsible ethics committee (Tübingen).

- **Fatal or life-threatening SUSARs**
  The competent authority and the ethics committee will be notified about all fatal or life-threatening SUSARs as soon as possible, but not later than 7 calendar days after the sponsor has first knowledge of the minimum criteria for expedited reporting. In each case relevant follow-up information will be sought and a report completed as soon as possible. It will be communicated to the competent authority and the Ethics Committee within an additional 8 calendar days.
- **Non fatal and non life-threatening SUSARs**
  All other SUSARs will be reported to the competent authority and the ethics committee as soon as possible, but not later than 15 calendar days after the sponsor has first knowledge of the minimum criteria for expedited reporting. Further relevant follow-up information will be given as soon as possible.

In case of incomplete information at the time of initial reporting, all the appropriate information for an adequate analysis of causality should be actively sought from the reporter or other available sources.
Within 15 days the sponsor informs the ethics committee and the competent authority about all circumstances that lead to re-evaluation of the risk-benefit ratio.

SAEs not classified as SUSAR will only be reported within the annual safety report.

9.5.2. Information of the Investigators

If new information becomes available that differs from the scientific information given in the information on the investigational medical intervention (see section 3. Introduction) and that may interfere with the safety of randomized patients or with the conduct of the study the principal coordinating investigator will provide this information to all investigators.

9.5.3. Notification of the Marketing Authorisation Holder

In addition, the sponsor reports all SUSARs to the responsible blood bank / manufacturer of the RBC concentrate including information on the previous notification to the competent authority (PEI) and ethics committee.

9.5.4. Notification Summary

Adverse Event (AE)

Local Center	informs	Principal Investigator (within 1 month)
Principal Investigator	informs	Sponsor (within 1 month)
		Authority (i.e., PEI) (upon request)
		Responsible EC (upon request)

Serious Adverse Event (SAE) – listed in 9.2.3.
(i.e., complications of prematurity)
(with the exception of death and blood culture positive sepsis)

Local Center	informs	Principal Investigator (within CRF)
Principal Investigator	informs	Sponsor (within IDMC report)
		Authority (i.e., PEI) (annual safety report)
		Responsible EC (annual safety report)

Serious Adverse Event (SAE) – not listed in 9.2.3.
(including death and blood culture positive sepsis)

Local Center	informs	Principal Investigator (immediately - within 24h)
Principal Investigator	informs	Sponsor (immediately - within 24 h)
		Authority (i.e., PEI) (annual safety report)
		Responsible EC (annual safety report)

Suspected Expected Serious Adverse Reaction (SESAR)

Local Center	informs	Principal Investigator (immediately - within 24h)
Principal Investigator	informs	Sponsor (immediately - within 24 h)
		Authority (annual safety report)
		Responsible EC (annual safety report)

Suspected Unexpected Serious Adverse Reaction (SUSAR)

Local Center	informs	Principal Investigator (immediately - within 24h)
Principal Investigator	informs	Sponsor (immediately - within 24 h)
		Authority (within 15 d)
		Responsible EC (within 15 d)
		Responsible Blood Bank

Fatal / Life Threatening Suspected Unexpected Serious Adverse Reaction (SUSAR)

Local Center → Principal Investigator (immediately - within 1 day)
Principal Investigator → Sponsor (immediately - within 1 day)

Local Center	informs	Principal Investigator (immediately - within 24h)
Principal Investigator	informs	Sponsor (immediately - within 24 h)
		Authority (within 7 d)
		Responsible EC (within 7 d)
		Responsible Blood Bank

Circumstances that Lead to a New Risk / Benefit Ratio

Principal Investigator	informs	Sponsor (immediately - within 24 h)
		All Local Investigators (within 15 d)
		Authority (within 15 d)
		Responsible EC (within 15 d)
Local Center	informs	all patients currently treated

9.6. Annual Safety Reports

In addition to the expedited reporting, the principal coordinating investigator will submit, once a year throughout the clinical trial or on request a safety report to the competent authority (PEI) and the ethics committee (Tübingen), taking into account all new available safety information received during the reporting period.

9.6.1. Content of the annual safety report of a clinical trial

- a listing of all SUSARs
- and an aggregate summary tabulation of SESARs

9.6.2. Reporting time frame for annual safety report

The reporting time frame for annual reports starts with the date of the first authorisation of the clinical trial by the competent authority (PEI).
This date is designated as the cut off for data to be included in the annual safety report. The principal investigator will submit annual reports within 60 days of the data lock point.

10. Use of Data and Publication

10.1. Reports

10.1.1. Interim Reports

When patient recruitment and investigational interventions are stopped or finished an interim analysis on the short term results will be performed. Other interim reports are not intended unless required by ethic committees or competent authorities.

10.1.2. Notification of Completion of the Clinical Trial

The principal coordinating investigators will inform the EC and the regulatory authorities (PEI) at latest 90 days after the last patient completed the 24 months follow-up examination about the closing of the clinical trial.

10.1.3. Final Report

The final report will present the results of the trial, including appropriate tables and figures in the spirit of an unbiased objectivity. The principal coordinating investigators will provide the EC and the regulatory authorities (PEI) with a summary of the trial's outcome at latest 1 year after the last patient completed follow-up.

10.2. Publication

Results of the study will be published after approval by the principal coordinating investigator, the members of the steering committee, and the statistician in an international scientific journal and presented on international scientific congresses. After termination of the study final results will be published. All publications will fulfil the CONSORT requirements (84-86). The first author will be the principal coordinating investigator. The order of co-authors depends on the number of infants contributed to the final analysis. The Steering Committee of ETTNO protects the academic interest of all local investigators: if a Journal does not accept at least 1 co-author from every center, the publication will be under a group authorship (The ETTNO Study Group) with all investigators listed in the Appendix.

All publications will maintain data protection of patient data as well as data of the participating investigators.

The publishing of data from a single study center is only permitted after analysis and primary publication of the final results. Publication of study results or data, including data of a single study center has to be reviewed and permitted by the principal coordinating investigator.

The "Uniform requirements for manuscripts submitted to biomedical journals: International Committee of Medical Journal Editors" (87) will be followed.

With signing the study protocol the local principal investigator agrees that the results are provided to regulatory authorities. Additionally, he agrees that herewith his name, address, qualification, and the extent of his involvement are disclosed.

11. Changes in the Conduct of the Study or Planned Analysis / Amendments

In order to maintain comparable conditions in all study centers and to obtain an unobjectionable data analysis, changes of the protocol are not intended.

If nonetheless changes to the protocol become necessary they are reported as amendment. The amendment has to be agreed upon by the principal coordinating investigator, the steering committee, the statistician, and the IDMC. It will be signed by the principal coordinating investigator and is thereafter part of the protocol.

An amendment has to be approved by the ethics committee responsible for the primary coordinating investigator and must be submitted to regulatory authorities when appropriate.

12. List of Appendices

11.1. ETTNO - Parent Information (in German)
11.2. ETTNO - Informed Consent Form and Data Protection Statement (in German)
11.3. ETTNO - List of Study Sites and Investigators
11.4. ETTNO - Local Authorities
11.5. ETTNO - Independent Data Monitoring Committee Manual (pending)
11.6. ETTNO - Monitoring Manual (pending)
11.7. ETTNO - Pharmacovigilance Manual (pending)
11.8. ETTNO - CRF (pending)
11.9. NEOKISS criteria for nosocomial infections
11.10. Insurance Certificate (pending)
11.11. ETTNO - SOP / Guideline for Trend-configuration and Data-Download from study pulse oximeter (pending)
11.12. Examination form of the Bayley Scales of Infant Development (2nd edition) in the German version
11.13. Initial publication of the PINT trial and of the Iowa trial on the effect of transfusion thresholds in preterm infants and all available follow-up data of these trials

13. References

1 Bifano EM, Curran TR. Minimizing donor blood exposure in the neonatal intensive care unit. Current trends and future prospects. Clin Perinatol 1995;**22**:657-69
2 Widness JA, Seward VJ, Kromer IJ, et al. Changing patterns of red blood cell transfusion in very low birth weight infants. J Pediatr 1996;**129**:680-7
3 Asch J, Wedgwood JF. Optimizing the approach to anemia in the preterm infant: is there a role for erythropoietin therapy? J Perinatol 1997;**17**:276-82
4 Linderkamp O, Zilow EP, Zilow G. [The critical hemoglobin value in newborn infants, infants and children]. Beitr Infusionsther 1992;**30**:235-46; discussion 247-64
5 Blanchette VS, Hume HA, Levy GJ, Luban NL, Strauss RG. Guidelines for auditing pediatric blood transfusion practices. Am J Dis Child 1991;**145**:787-96
6 Maier RF, Metze B, Obladen M. Low degree of regionalization and high transfusion rates in very low birthweight infants: a survey in Germany. J Perinat Med 1998;**26**:43-8
7 Bednarek FJ, Weisberger S, Richardson DK, et al. Variations in blood transfusions among newborn intensive care units. SNAP II Study Group [see comments]. J Pediatr 1998;**133**:601-7
8 Ringer SA, Richardson DK, Sacher RA, Keszler M, Churchill WH. Variations in transfusion practice in neonatal intensive care. Pediatrics 1998;**101**:194-200
9 Hume H. Red blood cell transfusions for preterm infants: the role of evidence-based medicine. Semin Perinatol 1997;**21**:8-19
10 Bassler D, Weitz M, Bialkowski A, Poets CF. Restrictive Versus liberal Red Blood Cell Transfusion Strategies for Preterm Infants:
A Systematic Review of Randomized Controlled Trials. Current Pediatric Reviews 2008;**4**:143-150
11 Busch MP, Kleinman SH, Nemo GJ. Current and emerging infectious risks of blood transfusions. Jama 2003;**289**:959-62
12 Cooke RW, Clark D, Hickey-Dwyer M, Weindling AM. The apparent role of blood transfusions in the development of retinopathy of prematurity. Eur J Pediatr 1993;**152**:833-6
13 Hesse L, Eberl W, Schlaud M, Poets CF. Blood transfusion. Iron load and retinopathy of prematurity. Eur J Pediatr 1997;**156**:465-70
14 Inder TE, Clemett RS, Austin NC, Graham P, Darlow BA. High iron status in very low birth weight infants is associated with an increased risk of retinopathy of prematurity. J Pediatr 1997;**131**:541-4
15 Cooke RW, Drury JA, Yoxall CW, James C. Blood transfusion and chronic lung disease in preterm infants. Eur J Pediatr 1997;**156**:47-50
16 Silvers KM, Gibson AT, Russell JM, Powers HJ. Antioxidant activity, packed cell transfusions, and outcome in premature infants. Arch Dis Child Fetal Neonatal Ed 1998;**78**:F214-9
17 Stainsby D, Jones H, Wells AW, Gibson B, Cohen H. Adverse outcomes of blood transfusion in children: analysis of UK reports to the serious hazards of transfusion scheme 1996-2005. Br J Haematol 2008;**141**:73-9
18 Swanson K, Dwyre DM, Krochmal J, Raife TJ. Transfusion-related acute lung injury (TRALI): current clinical and pathophysiologic considerations. Lung 2006;**184**:177-85

19 Silliman CC. The two-event model of transfusion-related acute lung injury. Crit Care Med 2006;**34**:S124-31

20 Joshi VH, Joshi PV, Webert K, et al. Does transfusion related acute lung injury (TRALI) occur in newborns? PAS-Abstracts 2006;**59**:460

21 Alagappan A, Shattuck KE, Malloy MH. Impact of transfusion guidelines on neonatal transfusions. J Perinatol 1998;**18**:92-7

22 Franz AR, Pohlandt F. Red blood cell transfusions in very and extremely low birthweight infants under restrictive transfusion guidelines: is exogenous erythropoietin necessary? Arch Dis Child Fetal Neonatal Ed 2001;**84**:F96-F100

23 Bell EF, Strauss RG, Widness JA, et al. Randomized trial of liberal versus restrictive guidelines for red blood cell transfusion in preterm infants. Pediatrics 2005;**115**:1685-91

24 Boedy RF, Mathew OP. Randomized trial of liberal versus restrictive guidelines for red blood cell transfusion in preterm infants. Pediatrics 2005;**116**:1048-9; author reply 1049-50

25 Murray N, Roberts I, Stanworth S. Red blood cell transfusion in neonates. Pediatrics 2005;**116**:1609; author reply 1609-10

26 Swamy RS, Embleton ND. Red blood cell transfusions in preterm infants: is there a difference between restrictive and liberal criteria? Pediatrics 2006;**117**:257-8; author reply 258-9

27 Kirpalani H, Whyte RK, Andersen C, et al. The Premature Infants in Need of Transfusion (PINT) study: a randomized, controlled trial of a restrictive (low) versus liberal (high) transfusion threshold for extremely low birth weight infants. J Pediatr 2006;**149**:301-307

28 Bell EF. Transfusion thresholds for preterm infants: how low should we go? J Pediatr 2006;**149**:287-9

29 Whyte RK, Kirpalani H, Asztalos EV, et al. Neurodevelopmental outcome of extremely low birth weight infants randomly assigned to restrictive or liberal hemoglobin thresholds for blood transfusion. Pediatrics 2009;**123**:207-13

30 Tschirch E, Weber B, Koehne P, et al. Vascular endothelial growth factor as marker for tissue hypoxia and transfusion need in anemic infants: a prospective clinical study. Pediatrics 2009;**123**:784-90

31 Knüpfer M, Seidel T, Gebauer C, et al. Wer soll transfundiert werden? Eine Selektion mittels Messung der Gewebeoxigenierung. Klinische Pädiatrie 2010;**222**:S88

32 Zhang B, Schmidt B. Do we measure the right end points? A systematic review of primary outcomes in recent neonatal randomized clinical trials. J Pediatr 2001;**138**:76-80

33 Krägeloh-Mann I, Ikonomidou C. Standardisierte Nachuntersuchung von Frühgeborenen - Konsensuspapier. Monatsschr Kinderheilk 2004;**152 Suppl 1**:KHF12.4

34 SCPE-working-, group. Surveillance of cerebral palsy in Europe: a collaboration of cerebral palsy surveys and registers. Surveillance of Cerebral Palsy in Europe (SCPE). Dev Med Child Neurol 2000;**42**:816-24

35 Christine C, Dolk H, Platt MJ, et al. Recommendations from the SCPE collaborative group for defining and classifying cerebral palsy. Dev Med Child Neurol Suppl 2007;**109**:35-8

36 Palisano R, Rosenbaum P, Walter S, et al. Development and reliability of a system to classify gross motor function in children with cerebral palsy. Dev Med Child Neurol 1997;**39**:214-23

37 International-Commmitee. The International Classification of Retinopathy of Prematurity revisited. Arch Ophthalmol 2005;**123**:991-9

38 Walsh MC, Wilson-Costello D, Zadell A, Newman N, Fanaroff A. Safety, reliability, and validity of a physiologic definition of bronchopulmonary dysplasia. J Perinatol 2003;**23**:451-6

39 Papile LA, Burstein J, Burstein R, Koffler H. Incidence and evolution of subependymal and intraventricular hemorrhage: a study of infants with birth weights less than 1,500 gm. J Pediatr 1978;**92**:529-34

40 de Vries LS, Eken P, Groenendaal F, van Haastert IC, Meiners LC. Correlation between the degree of periventricular leukomalacia diagnosed using cranial ultrasound and MRI later in infancy in children with cerebral palsy. Neuropediatrics 1993;**24**:263-8

41 Widdowson EM, Spray CM. Chemical development in utero. Arch Dis Child 1951;**26**:205-14

42 Yao AC, Moinian M, Lind J. Distribution of blood between infant and placenta after birth. Lancet 1969;**2**:871-3

43 Franz AR, Pohlandt F, Bode H, et al. Intrauterine, early neonatal, and postdischarge growth and neurodevelopmental outcome at 5.4 years in extremely preterm infants after intensive neonatal nutritional support. Pediatrics 2009;**123**:e101-9

44 Steinmacher J, Pohlandt F, Bode H, et al. Neuro-developmental follow-up at 5.6 years corrected age in infants born at 22 - 25 completed weeks of gestation following pro-active management at a gestational age of >/= 23 weeks. J Pediatr 2008;**152**:771-776

45 Steinmacher J, Pohlandt F, Bode H, et al. Randomized trial of early versus late enteral iron supplementation in infants with a birth weight of less than 1301 grams: neurocognitive development at 5.3 years' corrected age. Pediatrics 2007;**120**:538-46

46 Maier RF, Obladen M, Kattner E, et al. High-versus low-dose erythropoietin in extremely low birth weight infants. The European Multicenter rhEPO Study Group. J Pediatr 1998;**132**:866-70

47 Maier RF, Obladen M, Muller_Hansen I, et al. Early treatment with erythropoietin beta ameliorates anemia and reduces transfusion requirements in infants with birth weights below 1000 g. J Pediatr 2002;**141**:8-15

48 Franz AR, Bauer K, Schalk A, et al. Measurement of interleukin 8 in combination with c-reactive protein reduced unnecessary antibiotic therapy in newborn infants: a multicenter randomized controlled trial. Pediatrics 2004;**114**:1-8

49 Paul DA, Leef KH, Locke RG, Stefano JL. Transfusion volume in infants with very low birth weight: a randomized trial of 10 versus 20 ml/kg. J Pediatr Hematol Oncol 2002;**24**:43-6

50 Böhler T, Janecke A, Linderkamp O. Blood transfusion in late anemia of prematurity: effect on oxygen consumption, heart rate, and weight gain in otherwise healthy infants. Infusionsther Transfusionsmed 1994;**21**:376-9

51 Maier RF, Sonntag J, Walka MM, et al. Changing practices of red blood cell transfusions in infants with birth weights less than 1000 g. J Pediatr 2000;**136**:220-4

52 Murray NA, Roberts IA. Neonatal transfusion practice. Arch Dis Child Fetal Neonatal Ed 2004;**89**:F101-7

53 Wardle SP, Yoxall CW, Crawley E, Weindling AM. Peripheral oxygenation and anemia in preterm babies. Pediatr Res 1998;**44**:125-31
54 Wardle SP, Garr R, Yoxall CW, Weindling AM. A pilot randomised controlled trial of peripheral fractional oxygen extraction to guide blood transfusions in preterm infants. Arch Dis Child Fetal Neonatal Ed 2002;**86**:F22-7
55 Kayiran SM, Ozbek N, Turan M, Gurakan B. Significant differences between capillary and venous complete blood counts in the neonatal period. Clin Lab Haematol 2003;**25**:9-16
56 Mock DM, Bell EF, Lankford GL, Widness JA. Hematocrit correlates well with circulating red blood cell volume in very low birth weight infants. Pediatr Res 2001;**50**:525-31
57 Hebert PC, Wells G, Blajchman MA, et al. A multicenter, randomized, controlled clinical trial of transfusion requirements in critical care. Transfusion Requirements in Critical Care Investigators, Canadian Critical Care Trials Group. N Engl J Med 1999;**340**:409-17
58 Lacroix J, Hebert PC, Hutchison JS, et al. Transfusion strategies for patients in pediatric intensive care units. N Engl J Med 2007;**356**:1609-19
59 Bell EF. When to transfuse preterm babies. Arch Dis Child Fetal Neonatal Ed 2008;**93**:F469-73
60 Gesetz zur Regelung des Transfusionswesens. www.gesetze-im-internet.de/bundesrecht/tfg/gesamt.pdf
61 Richtlinien zur Gewinnung von Blut und Blutbestandteilen und zur Anwendung von Blutprodukten (Hämotherapie) gemäß §§ 12 und 18 des Transfusionsgesetzes (Novelle 2005). http://www.bundesaerztekammer.de/page.asp?his=0.7.45.3242
62 Querschnitts-Leitlinie zur Therapie mit Blutkomponenten und Plasmaderivaten. http://www.bundesaerztekammer.de/downloads/Blutkomponente_Querschnittsleitlinie_11032010.pdf
63 Dienstanweisung Vorbereitung und Durchführung von Bluttransfusionen am Universitätsklinikum Tübingen. http://www.med.uni-tuebingen.de/uktmedia/Mitarbeiter_Portal-p-170/PDF_Archiv-p-174/Transfusion/DABlut_UKT.pdf
64 Platt MJ, Krägeloh-Mann I, Cans C. Surveillance of Cerebral Palsy in Europe: Reference and Training Manual Medical Education 2009;**43**:495-496
65 Rabe H, Reynolds G, Diaz-Rossello J. A systematic review and meta-analysis of a brief delay in clamping the umbilical cord of preterm infants. Neonatology 2008;**93**:138-44
66 Franz AR, Mihatsch WA, Sander S, Kron M, Pohlandt F. Prospective randomized trial of early versus late enteral iron supplementation in infants with a birth weight of less than 1301 grams. Pediatrics 2000;**106**:700-6
67 Rönnholm KA, Siimes MA. Haemoglobin concentration depends on protein intake in small preterm infants fed human milk. Arch Dis Child 1985;**60**:99-104
68 Brown MS, Shapiro H. Effect of protein intake on erythropoiesis during erythropoietin treatment of anemia of prematurity. J Pediatr 1996;**128**:512-7
69 Agostoni C, Buonocore G, Carnielli V, et al. Enteral Nutrient Supply for Preterm Infants: Commentary From the European Society for Paediatric Gastroenterology, Hepatology, and Nutrition Committee on Nutrition. J Pediatr Gastroenterol Nutr 2010;**50**:85-91

70 Worthington-White DA, Behnke M, Gross S. Premature infants require additional folate and vitamin B-12 to reduce the severity of the anemia of prematurity. Am J Clin Nutr 1994;**60**:930-5
71 Maier RF, Obladen M, Messinger D, Wardrop CA. Factors related to transfusion in very low birthweight infants treated with erythropoietin. Arch Dis Child Fetal Neonatal Ed 1996;**74**:F182-6
72 Ohlsson A, Aher SM. Early erythropoietin for preventing red blood cell transfusion in preterm and/or low birth weight infants. Cochrane Database Syst Rev 2006;**3**:CD004863
73 Juul S. Recombinant erythropoietin as a neuroprotective treatment: in vitro and in vivo models. Clin Perinatol 2004;**31**:129-42
74 Roberts D, Dalziel S. Antenatal corticosteroids for accelerating fetal lung maturation for women at risk of preterm birth. Cochrane Database Syst Rev 2006;**3**:CD004454
75 Soll RF, Morley CJ. Prophylactic versus selective use of surfactant in preventing morbidity and mortality in preterm infants. Cochrane Database Syst Rev 2001:CD000510
76 Schmidt B, Roberts RS, Davis P, et al. Long-term effects of caffeine therapy for apnea of prematurity. N Engl J Med 2007;**357**:1893-902
77 Halliday HL, Ehrenkranz RA, Doyle LW. Early (< 8 days) postnatal corticosteroids for preventing chronic lung disease in preterm infants. Cochrane Database Syst Rev 2009:CD001146
78 Askie LM, Henderson-Smart DJ, Ko H. Restricted versus liberal oxygen exposure for preventing morbidity and mortality in preterm or low birth weight infants. Cochrane Database Syst Rev 2009:CD001077
79 Askie LM, Henderson-Smart DJ, Irwig L, Simpson JM. Oxygen-saturation targets and outcomes in extremely preterm infants. N Engl J Med 2003;**349**:959-67
80 Carlo WA, Finer NN, Walsh MC, et al. Target ranges of oxygen saturation in extremely preterm infants. N Engl J Med 2010;**362**:1959-69
81 Group TS-RS. Supplemental Therapeutic Oxygen for Prethreshold Retinopathy Of Prematurity (STOP-ROP), a randomized, controlled trial. I: primary outcomes. Pediatrics 2000;**105**:295-310
82 Schmidt B, Gillie P, Caco C, Roberts J, Roberts R. Do sick newborn infants benefit from participation in a randomized clinical trial? J Pediatr 1999;**134**:151-5
83 Dani C, Martelli E, Bertini G, et al. Effect of blood transfusions on oxidative stress in preterm infants. Arch Dis Child Fetal Neonatal Ed 2004;**89**:F408-11
84 Moher D, Schulz KF, Altman DG. The CONSORT statement: revised recommendations for improving the quality of reports of parallel-group randomised trials. Lancet 2001;**357**:1191-4
85 Moher D, Hopewell S, Schulz KF, et al. CONSORT 2010 explanation and elaboration: updated guidelines for reporting parallel group randomised trials. BMJ 2010;**340**:c869
86 Schulz KF, Altman DG, Moher D. CONSORT 2010 statement: updated guidelines for reporting parallel group randomised trials. BMJ 2010;**340**:c332
87 Editors ICoMJ. Uniform requirements for manuscripts submitted to biomedical journals. JAMA 1997;**277**:927-34
88 Strauss RG. Anaemia of prematurity: pathophysiology and treatment. Blood Rev 2010;**24**:221-5

89 Fredrickson LK, Bell EF, Cress GA, et al. Acute physiological effects of packed red blood cell transfusion in preterm infants with different degrees of anaemia. Arch Dis Child Fetal Neonatal Ed 2010 (epub ahead of print)

90 Alkalay AL, Galvis S, Ferry DA, Simmons CF, Krueger RC, Jr. Hemodynamic changes in anemic premature infants: are we allowing the hematocrits to fall too low? Pediatrics 2003;**112**:838-45

Amendment 1

Effects of transfusion thresholds on neurocognitive outcome of extremely low birth weight infants (ETTNO)

a blinded randomized controlled multicenter trial

Sponsor's Study Code: DFG Fr 1455/6-1

EudraCT-Number: 2010-021576-28

Version/Date: 1.0 / 23.10.2014

Summary: **Increase of sample size from 920 to 980 to adjust for currently anticipated higher than initially expected rate of loss-to-follow-up**

Universitätskinderklinik, Abt. Neonatologie
Calwerstr. 7, 72076 Tübingen

Date	Prof. Dr. med. Axel Franz<br>Principal Investigator ETTNO<br>Representative of the Sponsor

I. ORIGINAL SAMPLE SIZE

Summary:

**SAMPLE SIZE**	To be allocated to trial (i.e., randomized): (n = 920)<br>To be analysed: (n = 780, i.e., 2 x 390)

Original Sample Size and Power Calculation
The required sample size was calculated for the hypothesis underlying the research question:
"Do 'liberal' versus 'restrictive' RBCT improve or impair long-term outcome (i.e., reduce or increase the incidence of death or major neurodevelopmental impairment evaluated at 24 months corrected age) in extremely low birth weight infants?"

The required sample size was calculated based on the data recently reported for PINT participants [Whyte RK et al Pediatrics 2009].

Calculations were based on a X^2-test assuming a power of 80%, a two-sided significance level of 5%, an incidence of death or major neurodevelopmental impairment (where cognitive delay is defined as MDI<85) of 128/208 (61%) vs. 109/213 (51%) in the restrictive threshold and the liberal threshold group respectively [Whyte RK et al Pediatrics 2009]. Based on these assumptions, 390 patients are required in each arm (i.e., 390 patients with 'restrictive' RBCT guidelines and 390 with 'liberal' RBCT guidelines) to detect this absolute risk reduction of 10 percentage points.

Original Compliance / Rate of loss to follow up
Based on our recently completed study of long-term follow-up at 5.5 years corrected age in very low birth weight infants [Steinmacher J et al. Pediatrics 2007; Steinmacher J et al. J Pediatr 2007], we conservatively assume a 15% loss to follow-up rate for NICU survivors until 24 months corrected age. Consequently, approximately 920 have to be enrolled into the trial to ascertain the primary outcome in 780 patients.

II. *NEW* SAMPLE SIZE

Summary:

**SAMPLE SIZE**	To be allocated to trial (i.e., randomized): (n = 980)<br>To be analysed: (n = 780, i.e., 2 x 390)

Sample Size and Power Calculation (unchanged):
The required sample size was calculated for the hypothesis underlying the research question:
"Do 'liberal' versus 'restrictive' RBCT improve or impair long-term outcome (i.e., reduce or increase the incidence of death or major neurodevelopmental impairment evaluated at 24 months corrected age) in extremely low birth weight infants?"

The required sample size was calculated based on the data recently reported for PINT participants [Whyte RK et al Pediatrics 2009].

Calculations were based on a X^2-test assuming a power of 80%, a two-sided significance level of 5%, an incidence of death or major neurodevelopmental impairment (where cognitive delay is defined as MDI<85) of 128/208 (61%) vs. 109/213 (51%) in the restrictive threshold and the liberal threshold group respectively [Whyte RK et al Pediatrics 2009]. Based on these assumptions, 390 patients are required in each arm (i.e., 390 patients with 'restrictive' RBCT guidelines and 390 with 'liberal' RBCT guidelines) to detect this absolute risk reduction of 10 percentage points.

New Anticipated Rate of Loss-to-Follow-up

There no data of 24 months follow-up rates in the participating 40 ETTNO centers.
We adjusted the assumed rate of lost-to-follow-up to **20%** (previously 15%) for NICU survivors until 24 months corrected age, because initial entry into the study database is slow and local investigators report problems with follo-up. Consequently, approximately **980** (previously 920) have to be enrolled into the trial to ascertain the primary outcome in 780 patients.

Justification:
Since the initial application for the ETTNO trial in 2009, German multicenter follow-up experience has been gathered that raises concerns of lower follow-up rates: E.g., the German Neonatal Network Study (an epidemiological and genetic risk factor study) had follow-up rates at 2 years <70% (Prof. W. Göpel, Lübeck, personal communication). The Neurosis study (NCT01035190) currently *has ascertained complete long-term follow-up so far in about 75% of study infants, who should have completed follow-up to date (although the definite rate of lost to follow-up is only 10% so far and efforts are ongoing (Prof. D. Bassler, Zürich/Tübingen, personal communication)). Follow-up rates of the Phelbi trial are not available for technical reasons (Prof. U. Thome, Leipzig, personal communication). To prevent that less than desirable follow-up will reduce power of the trial - despite all efforts that are currently taken to achieve the ideal of >90% follow-up (!) - an amendment to the protocol seems to be required to enroll additional 60 patients (total now 980), thereby compensating for up to 20% lost-to follow-up.*

Funding for additional patients

The funding agency (DFG, Deutsche Forschungs Gemeinschaft) has approved the increase in sample size. The letter of approval is pending. (Dr. Wissing, DFG, personal communication)

Feasibility of Recruitment:

At the last safety report (August 2014) 800 patients have been enrolled into the ETTNO trial with acurrent recruitment of 25 patients per month.

ulm university universität uulm

Universität Ulm | 89069 Ulm | Germany

Medizinische Fakultät
Institut für Epidemiologie und Medizinische Biometrie

Leitung: Prof. Dr. D. Rothenbacher

Schwabstraße 13
89075 Ulm, Germany

Silvia Sander
Tel: +49 731 50-26904
Fax: +49 731 50-26902
silvia.sander@uni-ulm.de
http://www.uni-ulm.de/med/
epidemiologie-biometrie

ulm university universität
uulm

Statistical Analysis Plan
Version: 1
Date: 23-October-2018

for study

**Effects of transfusion thresholds on neurocognitive outcome of extremely low birth weight infants (ETTNO)
a blinded randomized controlled multicenter trial**

Sponsor's Study Code:	DFG Fr 1455/6-1
EudraCT-Number:	2010-021576-28
Phase:	IV (a study according to §4 para. 23 AMG)
Intervention:<br>Control Intervention:	Implementation of "liberal" versus "restrictive" guidelines for red blood cell transfusions (RBCT) in extremely low birth weight (ELBW) infants. Because neither "liberal" nor "restrictive" guidelines for red blood cell transfusions can be considered "standard" therapy in preterm infants, one will serve as control for the other.
Indication:	Treatment of anemia of prematurity and prevention of long-term neuro-developmental sequelae of prematurity
Design:	Prospective, observer-blinded, parallel group, randomized, controlled multicenter trial
Principal Investigator:	Axel Franz, Prof. Dr. med.<br>Universitätskinderklinik, Abt. Neonatologie, Calwerstr. 7, 72076 Tübingen
Sponsor:	Universitätsklinikum Tübingen<br>represented by M. Bamberg, Prof. Dr. med. and G. Sonntag and delegated Sponsorship to Universitätskinderklinik, Abt. Neonatologie represented by Christian-F. Poets, Prof. Dr. med., Calwerstr. 7, 72076 Tübingen
Version/Date:	1.3 / 18-MAY-2011
Amendment 1:	23-OCTOBER-2014

ETTNO is supported by the Deutsche Forschungsgemeinschaft (DFG Fr 1455/6-1, 6-2).
The protocol is the property of the Pediatric Center for Clinical Studies at the University Children's Hospital of Tuebingen.

Rainer Muche

Prof. Dr. Rainer Muche
23.10.2018

Prof. Dr. Martina Kron

Prof. Dr. Axel Franz

ulm university universität **uulm**

Statistical Analysis Plan

Version: 1

Date: 23-October-2018

for study

Effects of transfusion thresholds on neurocognitive outcome of extremely low birth weight infants (ETTNO) a blinded randomized controlled multicenter trial

Sponsor's Study Code:	DFG Fr 1455/6-1
EudraCT-Number:	2010-021576-28
Phase:	IV (a study according to §4 para. 23 AMG)
Intervention:<br>Control Intervention:	Implementation of "liberal" versus "restrictive" guidelines for red blood cell transfusions (RBCT) in extremely low birth weight (ELBW) infants. Because neither "liberal" nor "restrictive" guidelines for red blood cell transfusions can be considered "standard" therapy in preterm infants, one will serve as control for the other.
Indication:	Treatment of anemia of prematurity and prevention of long-term neuro-developmental sequelae of prematurity
Design:	Prospective, observer-blinded, parallel group, randomized, controlled multicenter trial
Principal Investigator:	Axel Franz, Prof. Dr. med.<br>Universitätskinderklinik, Abt. Neonatologie, Calwerstr. 7, 72076 Tübingen
Sponsor:	Universitätsklinikum Tübingen<br>represented by M. Bamberg, Prof. Dr. med. and G. Sonntag and delegated Sponsorship to Universitätskinderklinik, Abt. Neonatologie represented by Christian-F. Poets, Prof. Dr. med., Calwerstr. 7, 72076 Tübingen
Version/Date:	1.3 / 18-MAY-2011
Amendment 1:	23-OCTOBER-2014

ETTNO is supported by the Deutsche Forschungsgemeinschaft (DFG Fr 1455/6-1, 6-2). The protocol is the property of the Pediatric Center for Clinical Studies at the University Children's Hospital of Tuebingen.

M. Kron

Prof. Dr. Rainer Muche	Prof. Dr. Martina Kron	Prof. Dr. Axel Franz
	24-OKT-2018	

ulm university universität uulm

Statistical Analysis Plan
Version: 1
Date: 23-October-2018

for study

Effects of transfusion thresholds on neurocognitive outcome of extremely low birth weight infants (ETTNO) a blinded randomized controlled multicenter trial

Sponsor's Study Code:	DFG Fr 1455/6-1
EudraCT-Number:	2010-021576-28
Phase:	IV (a study according to §4 para. 23 AMG)
Intervention: Control Intervention:	Implementation of "liberal" versus "restrictive" guidelines for red blood cell transfusions (RBCT) in extremely low birth weight (ELBW) infants. Because neither "liberal" nor "restrictive" guidelines for red blood cell transfusions can be considered "standard" therapy in preterm infants, one will serve as control for the other.
Indication:	Treatment of anemia of prematurity and prevention of long-term neuro-developmental sequelae of prematurity
Design:	Prospective, observer-blinded, parallel group, randomized, controlled multicenter trial
Principal Investigator:	Axel Franz, Prof. Dr. med. Universitätskinderklinik, Abt. Neonatologie, Calwerstr. 7, 72076 Tübingen
Sponsor:	Universitätsklinikum Tübingen represented by M. Bamberg, Prof. Dr. med. and G. Sonntag and delegated Sponsorship to Universitätskinderklinik, Abt. Neonatologie represented by Christian-F. Poets, Prof. Dr. med., Calwerstr. 7, 72076 Tübingen
Version/Date:	1.3 / 18-MAY-2011
Amendment 1:	23-OCTOBER-2014

ETTNO is supported by the Deutsche Forschungsgemeinschaft (DFG Fr 1455/6-1, 6-2).
The protocol is the property of the Pediatric Center for Clinical Studies at the University Children's Hospital of Tuebingen.

Prof. Dr. Rainer Muche   Prof. Dr. Martina Kron   Prof. Dr. Axel Franz
24.10.2018

Table of Contents:

1. Introduction 5
2. Study Design 5
3. Sample Size Calculation 8
4. Analyses Sets 9
5. Handling of Missing Values and Analysis Conventions 11
6. Subject Accountability 12
7. Protocol Deviations 13
8. Premature Discontinuation and Final Study Status 13
9. Patient Characteristics 14
   9.1 Screening Visit 14
   9.2 Baseline Visit 14
   9.3 Hematocrit and Red Blood Cell Transfusion 16
10. Efficacy Analysis 18
   10.1 Primary Endpoint 18
   10.2 Secondary and Other Endpoints 19
   10.3 Other Post Baseline Assessments 21
   10.4 Subgroup Analysis 25
11. Safety Analyses 25
12. List of Data provided by the Data Management in addition to ETTNO 26
database read-out ....

List of Abbreviations

AE	Adverse Event
AWMF	Arbeitsgemeinschaft der wissenschaftlich-medizinischen Fachgesellschaften
BPD	Bronchopulmonary Dysplasia
CI	Confidence Interval
CP	Cerebral Palsy
CPAP	Continuous Positive Airway Pressure
CPCS	Center for Pediatric Clinical Studies
CRF	Case Report Form
EC	Exclusion Criteria
ELBW	Extremely Low Birth Weight
FU	Follow up
GA	Gestational age
IC	Inclusion Criteria
ITT	Intention-To-Treat
IVH	Intraventricular Hemorrhage
MDI	Mental Developmental Index
mITT	modified Intention-To-Treat
NEC	Necrotizing Enterocolitis
NICU	Neonatal Intensive Care Unit
PDA	Patent Ductus Arteriosus
PDI	Physical Developmental Index
PMA	postmenstrual age
PP	Per-Protocol
PVL	Periventricular Leukomalacia
RBCT	Red blood cell transfusion
ROP	Retinopathy of Prematurity
SAE	Serious Adverse Event

1. Introduction

This Statistical Analysis Plan (SAP) describes the analyses for the study "Effects of transfusion thresholds on neurocognitive outcome of extremely low birth weight infants (ETTNO) - a blinded randomized controlled multicenter trial" protocol dated 18. May 2011 and amendment 1 dated 23. October 2014.
The objective of the SAP is to ensure the maximum credibility of all study findings by means of a prespecified data analysis. This plan will not repeat all the definitions given in the protocol, but will provide further details of the analyses planned therein. Further to the information set out in the protocol, minor and major protocol deviations are defined.
Data management, data checking, queries and data corrections were done by the Center for Pediatric Clinical Studies (CPCS), Ressort IV, University of Tübingen.
This SAP specifies the analysis of data until discharge plus additional 24 months data.
The analysis of the ancillary study A, B, C, D will be described in a separate SAP.
The statistical analysis will be done with SAS Version 9.4. The output of the statistical analyses (e.g. tables, figures, listings) will be provided to the University of Tübingen for preparation of the integrated report.

2. Study Design

This study is a prospective, observer-blinded, parallel group, randomized, controlled trial and was designed to compare the effect of "liberal" versus "restrictive" red blood cell transfusion (RBCT) guidelines on long-term neurodevelopmental outcome in extremely low birth weight (ELBW) infants.
ELBW infants were randomized to one of the two groups:

- RBCT according to **"liberal"** guidelines
- RBCT according to **"restrictive"** guidelines

The randomization was stratified for center and the two birth weight categories (400-749g / 750-999g).
There wasn't any blinding or study-specific label on the red blood cell concentrates, because these blood products were not in any way different from standard blood products and did not differ between the study groups. The only investigational procedure is the difference in hematocrit thresholds between treatment groups triggering the indication for the RBCT.

The examinations and study procedures are illustrated in Figure 1.

Figure 1: Study Time Table for the individual patient

AGE	Day 1-2	48-72 hours	Day <3	…	Discharge	12 months	24 months corrected age
	Hospital Admission						
ACTION	Screen for eligibility.<br><br>If eligible: approach & inform parents.	Perform cranial ultrasound<br><br>Randomize patient if still eligible and if consent was obtained	Follow RBCT guidelines	Follow RBCT guidelines until discharge from hospital in all patients.<br><br>In all patients until discharge:<br>Iron and protein supplementation as defined in the study protocol<br><br>Neonatal care according to awmf- and center guidelines as defined in the study protocol<br><br>Monitor blood count, growth, markers of oxidative stress, etc as defined in the study protocol<br><br>Document all adverse events as defined in the study protocol	Remind parents to Follow-up assessment FU.<br><br>Inform Pediatrician about study and FU.<br><br>Continue iron supple-mentation until 12 months as recommen-ded.	Send birth day card and<br><br>Remind parents of FU	Obtain anthropo-metric data.<br><br>Perform formal neurological examination.<br><br>Perform Bayley Scales of Infant Development.<br><br>Verify ophthamological report.<br><br>Fill in 24-months questionnaire.

The planned number of patients (screening to determination) is shown in Figure 2.

Figure 2: Study Flow Chart

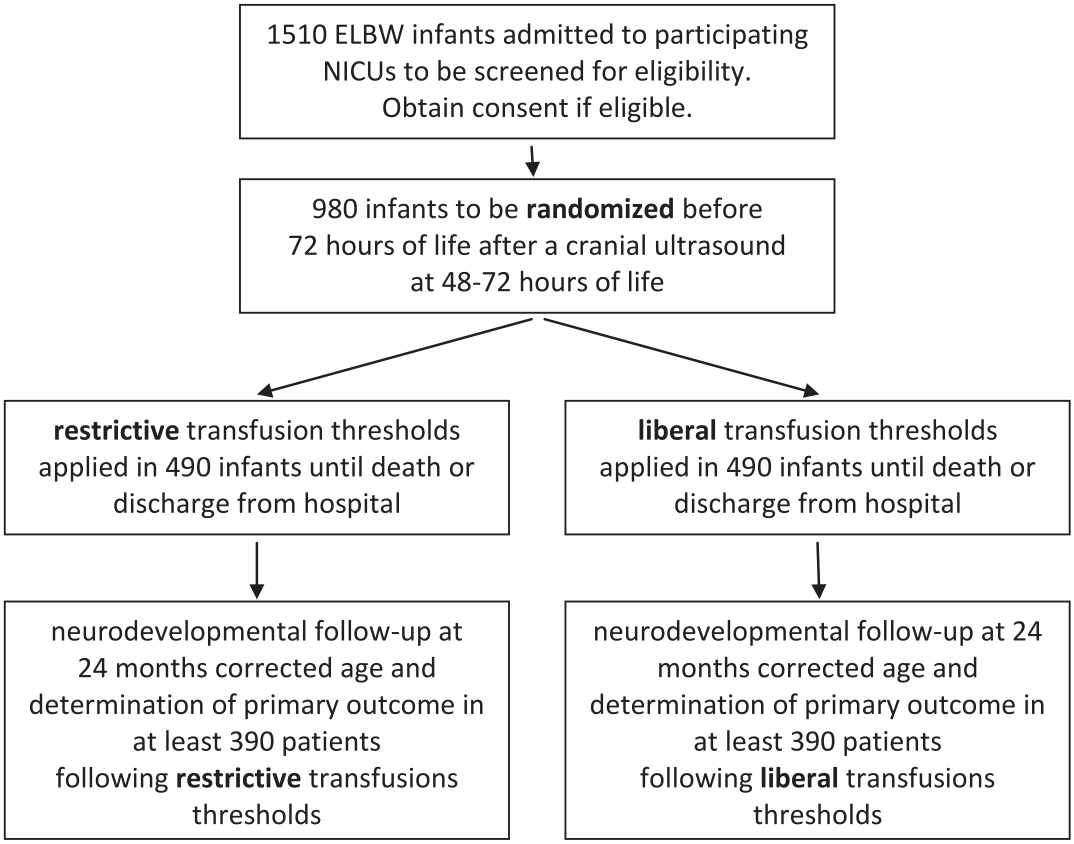

The following thresholds for RBCT were applied:

Figure 3: Thresholds for RBCT

	Restrictive RBCT Thresholds (venous hematocrit)		***Liberal RBCT Thresholds*** (venous hematocrit)	
State of health	'critical'	'non-critical'	'critical'	'non-critical'
After randomization:				
≤7 days of age	<34%	<28%	<41%	<35%
8-21 days of age	<30%	<24%	<37%	<31%
>21 days of age	<27%	<21%	<34%	<28%

Where a '*critical* state of health' was defined as the presence of any of the following:

- requirement of mechanical ventilation (any mode, excluding continuous positive airway pressure (CPAP))
- requirement of CPAP with FiO2>0,25 for >12h per 24h
- patent ductus arteriosus (PDA) requiring therapy
- more than 6 apnea that require stimulation per 24h or more than 4 desaturations to SpO2<60% per 24h despite methylxanthines and CPAP
- acute sepsis or acute NEC requiring inotropic or vasopressor support

Centers may choose to use thresholds of hemoglobin concentrations (Hb in g/dl) rather than of hematocrit values (as listed above). The respective hemoglobin thresholds are derived by dividing the hematocrit value displayed in Figure 3 above by 3. These hemoglobin thresholds have then to be used throughout the study.

The primary outcome measure is the incidence of death or major neurodevelopmental impairment determined at 24 months of age corrected for prematurity.

Secondary long-term outcomes are the incidences of individual components of the composite primary outcome, the mental and physical developmental index scores of the Bayley Scales of Infant Development (II edition), growth and mortality until follow-up but also short-term outcomes such as duration of respiratory support and hospital stay, in-hospital growth and mortality, and the incidences of the major diseases of prematurity (i.e., bronchopulmonary dysplasia (BPD), retinopathy of prematurity (ROP), necrotizing enterocolitis (NEC), intestinal perforation, brain injury on cranial ultrasound, and patent ductus arteriosus (PDA) requiring therapy).

Safety analyses will assess the incidences of all major diseases of prematurity.

3. Sample Size Calculation

SAMPLE SIZE To be allocated to trial (i.e., randomized): (n = 980)
To be analyzed: (n = 780, i.e., 2 x 390)

Sample Size and Power Calculation:
The required sample size was calculated for the hypothesis underlying the research question: “Do ‘liberal’ versus ‘restrictive’ RBCT improve or impair long-term outcome (i.e., reduce or increase the incidence of death or major neurodevelopmental impairment evaluated at 24 months corrected age) in extremely low birth weight infants?”
The required sample size was calculated based on the data recently reported for PINT participants [Whyte RK et al Pediatrics 2009].
Calculations were based on a χ^2-test assuming a power of 80%, a two-sided significance level of 5%, an incidence of death or major neurodevelopmental impairment (where cognitive delay is defined as Mental Developmental Index (MDI)<85) of 128/208 (61%) vs. 109/213 (51%) in the restrictive threshold and the liberal threshold group respectively [Whyte RK et al Pediatrics 2009].
Based on these assumptions, 390 patients are required in each arm (i.e., 390 patients with ‘restrictive’ RBCT guidelines and 390 with ‘liberal’ RBCT guidelines) to detect this absolute risk reduction of 10 percentage points.
The assumed rate of lost-to-follow-up is **20%** for Neonatal Intensive Care Unit (NICU) survivors until 24 months corrected age, because initial entry into the study database is slow and local investigators report problems with follow-up. Consequently, approximately **980** have to be enrolled into the trial to ascertain the primary outcome in 780 patients.

4. Analyses Sets

The following study populations will be defined:

- **Screening population:** consists of all screened patients and will be used just to describe the relation between screened and randomized patients and reasons for non-randomization.
- **Safety population:** consists of all randomized patients, treated according to either investigational transfusion threshold and will be the basis for all safety analysis.
- **Intention-to-treat (ITT) population:** consists of all randomized patients. Patients who changed therapy and patients who were not treated according to protocol for any reason will be analyzed in the group they have been randomized to.
- **Modified intention-to-treat (mITT) population:** consists of all randomized patients in whom the **primary outcome** was ascertained. Patients who changed therapy and patients who were not treated according to protocol for any reason will be analyzed in the group they have been randomized to.
- **Per-protocol (PP) population:** consists of all patients of the ITT population with the exception of those patients, who received any transfusion outside the assigned threshold indications and those patients, who were not transfused despite having hematocrit values below the thresholds. In the unlikely event that a patient changed therapy immediately after randomization, this patient will be analyzed in the group he/she has been treated.

Criteria for defining the **Screening** population

Dataset		*Condition*
SC		all patients in dataset

All patients screened for the study – even if screening failure – belong to the screening population.

Criteria for defining the **Safety** population

Dataset	*Variable*	*Label*	*Condition*
BA	RANDOM_TREATMENT	Treatment assigned	1 (=restrictive RBCT) or<br>2 (=liberal RBCT)

A patient belongs to the safety population if the patient has been randomized, that means if a study therapy is documented.

Criteria for defining the **ITT** population

Dataset	*Variable*	*Label*	*Condition*
BA	RANDOM_TREATMENT	Treatment assigned	1 (=restrictive RBCT) or<br>2 (=liberal RBCT)

The safety population and the ITT population are identical.

Criteria for defining the **modified ITT (mITT)** population

Dataset	*Variable*	*Label*	*Condition*
BA	RANDOM_TREATMENT	Treatment assigned	1 (=restrictive RBCT) or<br>2 (=liberal RBCT)
FU<br>CPCS_CD	MDI or<br>MDI_ADD<br><br><br>FU_KLASSIFIKATION<br><br>FU_VORHANDEN<br><br>FU_BEURTEILUNG05<br><br>FU_BEURTEILUNG06	MDI score or<br>alternative classification of cognitive delay<br><br>Overall classification<br><br>type of CP<br><br>Severe visual impairment<br><br>Hearing aid prescribed	not missing or<br>not missing (i.e., 0 or 1) }<br><br>and<br>Overall classification =1 or 2,<br>if Overall classification =3<br>then type of CP not missing }<br>and<br>not missing<br>and<br>not missing
	or		
FU<br>CPCS_CD	MDI or<br>MDI_ADD<br><br><br>FU_VORHANDEN<br><br>FU_BEURTEILUNG05<br><br>FU_BEURTEILUNG06	MDI score or<br>alternative classification of cognitive delay<br><br><br>type of CP<br><br>Severe visual impairment<br><br>Hearing aid prescribed	<85 or<br>1 (=cognitive delay present) }<br><br>or<br>1,2,3 or 4<br>or<br>yes<br>or<br>yes
	or		
ES	REASON_EOS	Reason for study termination	2 (=death before discharge)<br>3 (=death after discharge)
	or		
NOEH	REASON_EOH	Reason for end of hospitalization	1 (=infant died)

The modified ITT-population consists of all patients of the ITT-population for whom the primary endpoint is available. To define the primary endpoint the items mentioned above are used. If the above mentioned conditions are fulfilled, the primary endpoint can be assigned a value and therefore the child belongs to the modified ITT-population.

Per-protocol (PP) population:

Criteria for defining the **PP** population will be defined prior to unblinding in a separate document.

5. Handling of Missing Values and analysis conventions

In the ITT analysis for the MDI score as part of the primary outcome variable cognitive delay (yes or no), missing values (of children who are so severely handicapped that they cannot be tested or for children lost to follow-up and rated as non-testable (because of a severe cognitive deficit) by the Pediatrician caring for the infant) have been imputed according to a pre-defined scheme as described in the study protocol:

- Children whose severe cognitive impairment or disability precludes the use of the Bayley Scales of Infant Development will be assigned an MDI score of 30 if minimal speech and the ability for minimal communication with the parents are present, and an MDI score of 20 if no speech is present but at least minimal sensory or motor achievements are elicited.
- Children who are tested but reach a raw score corresponding to an MDI value of less than 50, will be assigned an MDI score of 49.

This scheme is only used for the definition of a cognitive delay (yes or no). I.e., for evaluation of the quantitative MDI score, the imputed values are not used.
If a different neurocognitive assessment was performed instead of Bayley 2 MDI, this other test only can be used for the definition of a cognitive delay (yes or no).
A permanent SAS-file (‘CPCS_CD’) was created by the coordinating investigator, an independent psychologist, the data management and the lead monitor. In this file, other neurocognitive assessments were rated (variable: MDI_ADD) indicating a cognitive delay (yes if MDI_ADD =1 or no if MDI_ADD=0). These assignments were done while strictly being blinded to treatment group assignment. Only unanimous ratings among coordinating investigator, independent psychologist and lead monitor were used. If available data were insufficient to reach a unanimous rating the criterion “cognitive delay” was deemed missing.

Primary endpoint

The composite primary outcome “death or major neurodevelopmental impairment” will be considered present (i.e. “yes”), if 1 or more of the individual components of the composite outcome are known to be present (i.e. have been documented as “yes”), or absent (i.e. “no”), if all components are known to be absent (i.e. have been documented as “no”). If no component is present, 1 or more missing components will cause the primary outcome to be deemed missing.

Only in the ITT population a worst case scenario analysis will be performed following imputation of “death or neurodevelopmental impairment” for all infants in whom the primary outcome was not ascertained (i.e., including those infants lost to follow-up).
In the PP population no strategies for imputing missing values will be applied.

Secondary endpoints
For secondary outcome variables no imputation strategies for missing values will be applied.

General conventions
Time intervals are usually calculated as days from birth until the event.
In addition, for analysis of the corrected age of the child at follow-up, the “estimated date of delivery / date of term equivalent (PMA 40 weeks 0 days)” is calculated as DATE_PMA23 (date of postmenstrual age of 23 weeks and 0 days) + 119 days.
Also the DATE_PMA36 (date of PMA of 36 weeks and 0 days) is calculated as DATE_PMA23 + 91days.

6. Subject Accountability

An overview of the number of subjects in each analysis set by treatment group will be provided overall and by center.

Furthermore, frequencies of potential reasons for non-inclusion of screened patients will be listed / tabulated according to the following variables:

Dataset	*Variable*	*Label*	*Condition*
SC<br><br><br><br>CPCS_ BW	INCLUSION_WEIGHT_BIRTH<br><br>WEIGHT_SC_MOD<br><br>CAUSE1	Birth weight 400 - 999 g<br><br>Birth weight<br><br>Birth weight out of range according to free text field in SC-eCRF	no<br>or<br><400<br>or<br>1=birth weight >999g<br>0=unknown
SC	INCLUSION_GESTAGE	Gestational age (GA) at birth 22+0/7 to 29+6/7 weeks	no
	EXCLUSION_DEATH	Baby died before 48h	yes
	EXCLUSION_VIABLE	Not considered viable	yes
	EXCLUSION_ABBERRATIONS	Chromosomal Aberrations	yes
	EXCLUSION_OUTCOME	Syndromes affecting long-term outcome	yes
	EXCLUSION_MAL-FORMATIONS	Congenital malformations	yes
	EXCLUSION_HEART_ DISEASE	Cyanotic heart disease	yes
	STUDY_AVAILABLE	Higher order infant	yes
	STUDY_PARTICIPATION	Randomized into another study	yes
	PARENT_APPROACHING	Parents approached	no
	CONSENT_OBTAINED	Consent obtained	no

7. Protocol Deviations

ICH-Protocol deviations are defined as follows:

- Violation of Inclusion Criteria (IC)/ Exclusion Criteria (EC)
  - IC: Birth weight 400-999g
  - IC: Gestational age at birth 22 0/7 to 29 6/7 weeks
  - EC: Baby died before 48 hours
  - EC: Baby not considered viable or comfort care only at 48 hours
  - EC: Known or strongly suspected chromosomal aberrations
  - EC: Known or strongly suspected syndromes affecting long-term outcome
  - EC: Known or strongly suspected major congenital malformations
  - EC: Known or strongly suspected cyanotic heart disease
  - EC: second / third born or higher order infant of a multiple delivery of which any lower order infant is available for the study
  - EC: randomized into another study
- Wrong treatment (definition and analysis see separate document defining the PP population)
- Prohibited concomitant medication: erythropoietin after randomization (documented by onsite monitoring visits on handwritten monitoring reports): all monitoring reports were reviewed and no erythropoietin treatment after randomisation was documented in any patient

Protocol deviations will be analyzed by absolute and relative frequencies in each treatment group for the ITT and mITT analysis set.

8. Premature Discontinuation and Final Study Status

The reasons for study termination will be analyzed in the ITT and the mITT population by absolute and relative frequencies in each treatment group and overall. Reasons for study termination will be:

- end of study according to protocol
- death (before and after discharge home)
- lost to follow-up (after transfer before discharge home and after discharge home)
- withdrawn consent (before and after discharge home)
- other

In addition, interval until end of study and interval until death including whether an autopsy has been performed will be analyzed. All infants who died will be listed with cause of death by treatment group according to the classification provided in a permanent SAS-file ('CPCS_RD') by the data management, where reason (variable: REASONDEATH) for death will be:

- Pulmonary Complications (=1)
- Cardiac complication (=2)
- Gastrointestinal complication (=3)
- Sepsis/Infection (=4)
- IVH/PVL (=5)

- Renal complication (=6)
- Multiorgan failure (=7)
- Prematurity/ELBW (=8)
- Other reasons for death (=9)

9. Patient Characteristics

Descriptive statistics will be provided and will include counts and percentages for categorical variables and mean, standard deviation, minimum, first quartile, median, third quartile and maximum for quantitative variables.
For categorical data chi-square tests or Fisher’s exact test will be used to compare treatment groups, continuous data will be compared between treatment groups with ANOVA or Wilcoxon tests. Statistical tests will only be applied if assigned in the following.

9.1 Screening Visit

The following variables collected at the screening visit will be analyzed in the Screening population, ITT population and mITT population.
Gestational age in weeks+days at birth (statistical test) (CRF: SC, variable: WEEK_GESTAGE_BIRTH, ADDDAY_GESTAGE_BIRTH)

9.2 Baseline Visit

The following variables collected at the baseline assessment before and at randomization will be analyzed in the ITT population and mITT population.

Prior medication and supplements before randomization: (CRF: BA)
- Surfactant (variable: BA_ANTWORT01)
- Caffeine or other methylxanthine (variable: BA_ANTWORT02)
- Catecholamines (variable: BA_ANTWORT03)
- Systemic corticosteroids (variable: BA_ANTWORT04)
  if yes, reason for systemic corticosteroids (variable: REASON_CORTICO)
- Inhaled corticosteroids (variable: BA_ANTWORT05)
- Inhaled nitric oxide (variable: BA_ANTWORT06)
- Indomethacin / ibuprofen prophylaxis (variable: BA_ANTWORT07)

Complications of prematurity before randomization: (CRF: BA)
- Early onset sepsis (variable: BA_ANTWORT08)
- PDA requiring therapy (NOT prophylaxis) (variable: BA_ANTWORT09)
- if yes, Mode of therapy (variable: BA_BEHANDLUNG01)
- Focal intestinal perforation (variable: BA_ANTWORT10)

- if yes, Mode of therapy (variable: BA_BEHANDLUNG02)
- NEC > IIa (variable: BA_ANTWORT11)
- if yes, Mode of therapy (variable: BA_BEHANDLUNG03)
- Air leak requiring pleural drainage (variable: BA_ANTWORT12)
- Pulmonary hemorrhage requiring transfusion (variable: BA_ANTWORT13)
- **HUS at Day 0:** (CRF: XHU)
  - Normal result of HUS (variable: RESULT_HUS)
    if not normal,
    Maximum grade of intraventricular / periventricular hemorrhage (variable: HUS_UNNORMAL01)
    Flare (variable: HUS_UNNORMAL02)
    Cystic periventricular leukomalacia (PVL) (variable: HUS_UNNORMAL03)
    Porencephalic cyst (variable: HUS_UNNORMAL04)
    Ventriculomegaly (variable: HUS_UNNORMAL05)
    Cortical atrophy (variable: HUS_UNNORMAL06)
    Other result (variable: HUS_UNNORMAL07)
  - HUS result group: HUS_UNNORMAL01=3,4 or HUS_UNNORMAL03=1,2 or
    HUS_UNNORMAL04=yes

Treatment for anemia of prematurity before randomization: (CRF: BA)
- Enteral iron supplementation (variable: BA_ANTWORT14)
- Parenteral iron supplementation (variable: BA_ANTWORT15)
- Erythropoietin (variable: BA_ANTWORT16)
- Red blood cell transfusion (variable: BA_ANTWORT17)
  if yes, Number of red blood cell transfusions (variable: ANZAHL_RBC)
  Total volume [ml] of transfused red blood cells (variable: VOL_RBC)
  overall: Number of red blood cell transfusions (variable: ANZAHL_RBC)
  Total volume [ml] of transfused red blood cells (variable: VOL_RBC)
  where ANZAHL_RBC and VOL_RBC is set to “0”
  in patients with no red blood cell transfusion

Respiratory support at randomization: (CRF: BA)
- Supplemental oxygen (variable: SUPPL_OXYGEN)
- Positive airway pressure (variable: BA_ANTWORT18)

Maternal data at delivery: (CRF: MD)
- Maternal age in years at birth (variable: MATERNAL_DATE_BIRTH)
- Maternal ethnicity (variable: MATERNAL_ETHNICITY)
- Family constellation (variable: FAMILY)
- Maternal education (variable: EDUCATION_MATERNAL)
- Maternal occupation (variable: OCCUPATION_MATERNAL)
- Antenatal steroids given (variable: MD_MED01)
- Mother received tocolytic therapy (variable: MD_MED02)
- Mother received antibiotics (variable: MD_MED03)

- Mother received insulin for insulin-dependent or gestational diabetes (variable: MD_MED04)
- Membranes rupture more than 24h before delivery (variable: MD_ERKRANKUNG01)
- Clinical diagnosis of chorioamnionitis (variable: MD_DIAGNOSE01)
- Histological diagnosis of chorioamnionitis (variable: MD_DIAGNOSE02)
- Placental abruption occur (variable: MD_ERKRANKUNG02)
- Mode of delivery (variable: MD_KLASSIFIKATION)

Indication for delivery (multiple answers permitted): (CRF: MD)
- Unsupressible labor (variable: MD_ART01)
- Pre-eclampsia, HELLP, pregnancy induced hypertension (variable: MD_ART02)
- Chorioamnionitis (variable: MD_ART03)
- Decelarations on CTG, flat CTG or pathological doppler examination (variable: MD_ART04)

Infant data at birth: (CRF: ID)
- Weight (statistical test) (CRF: SC, variable: WEIGHT_SC_MOD)
- Weight group: WEIGHT_SC_MOD 400-749g vs. WEIGHT_SC_MOD 750-999g
- Gender (statistical test) (variable: GENDER)
- Location of birth (variable: BIRTH_LOCATION)
- Multiple birth (variable: BIRTH_MULTIPLE)
- Birth order of study baby (variable: BIRTH_ORDER)
- Procedure of DCC or milking of the cord performed (variable: ID_UNTERSUCHUNG)
- Intubation at birth (variable: ID_BEHANDLUNG01)
- Chest compression at birth (variable: ID_BEHANDLUNG02)
- Adrenaline / epinephrine at birth (variable: ID_BEHANDLUNG03)
- Umbilical cord pH, venous (variable: CORDPH_VENOUS)
- Umbilical cord pH, arterial (variable: CORDPH_ARTERIAL)
- Apgar score at 1 minute (variable: APGAR_1)
- Apgar score at 5 minutes (variable: APGAR_5)
- First temperature [°C] after admission to the NICU (variable: TERMPERATURE)
- Mode of temperature measurement (variable: ID_METHODE)
- Body length at birth [cm] (variable: LENGTH_ID_MOD)
- Head circumference at birth [cm] (statistical test) (variable: HEAD_CIRCUMFENCE_ID_MOD)

9.3 Hematocrit and Red Blood Cell Transfusion during study

Hematocrit values (CRF: WA, HEMATOCRIT01 - HEMATOCRIT07) from the weekly assessment will be displayed as mean course over time by treatment group in ITT population and mITT population. No statistical test will be performed.

Red blood cell transfusions (CRF: RBC) will be described as follows:
- Number of blood transfusions (total and per infant)
- Total volume transfused (ml) (variable: VOL_RBC) during study treatment
- Volume transfused (ml/kg) for each transfusion and during study treatment
- Infants clinical condition state (variable: RBC_EREIGNIS01)

For each clinical condition state (“critical”, “non-critical”) (variable: RBC_EREIGNIS01):
- Number of blood transfusions (total and per infant)
- Total volume transfused (ml) (variable: VOL_RBC) during study treatment
- Volume transfused (ml/kg) for each transfusion and during study treatment

Additionally, if clinical condition state = “critical” (variable: RBC_EREIGNIS01=critical):
- Requirement of mechanical ventilation via ET-tube (variable: RBC_EREIGNIS02)
- Requirement of CPAP with FiO2 > 0.25 for > 12h per 24h (variable: RBC_EREIGNIS03)
- PDA requiring therapy (variable: RBC_EREIGNIS04)
- More than 6 apnea that required stimulation per 24h (variable: RBC_EREIGNIS05)
- Acute sepsis or acute NEC requiring inotropic or vasopressor support (variable: RBC_EREIGNIS06)

- Transfusion according to the pre-defined hematocrit / hemoglobin transfusion thresholds (variable: RBC_BEHANDLING)
  - if NO, Major hemorrhage (variable: RBC_GRUND01)
    - Lactic acidosis > 4 mmol/l (variable: RBC_GRUND02)
    - Major surgery (variable: RBC_GRUND03)
    - Other unforeseen emergency (variable: RBC_GRUND04),
      - if yes, *this transfusion* (indicated by RBC_date) being classified in permanent SAS-file (‘CPCS_EM’) as sufficiently justified (variable: justified=1)

These analyses of the transfusions will be done until PMA 36 weeks. Additionally for PMA>36 weeks the following variables will be analyzed:
- Number of blood transfusions (total and per infant)
- Total volume transfused (ml) (variable: VOL_RBC) during study treatment
- Volume transfused (ml/kg) for each transfusion and during study treatment

10. Efficacy Analysis

The analysis of the primary outcome variable in the mITT population of all randomized subjects in whom the primary outcome was ascertained will be considered confirmatory.
Analyses of secondary and other endpoints as well as other post baseline assessments will be done in the ITT population and are considered explorative.

As a sensitivity analysis, primary, secondary and other endpoints will be analyzed in the PP-population

All statistical tests will be two-sided at a significance level of 5%.
Confidence intervals (CI) will be presented using the 95% confidence level.
Analysis of PP population will be omitted if the difference in total sample size to the mITT-set is less than 10%.

Follow-Up assessments for the study children were scheduled at 24 months +/- 1 month corrected age to determine the primary outcome.

10.1 Primary Endpoint

The primary outcome of this study will be the long-term neurocognitive development measured as the occurrence of at least one of the following components:

- **Death** or
- **Major neurodevelopmental impairment determined at 24 months of age corrected for prematurity,** where major neurodevelopmental impairment is defined as any of the following four components:
    - **cognitive delay** defined as mental developmental index (**MDI**) score of the Bayley Scales of Infant Development (2nd edition) < 85 or other neurocognitive assessments indicating cognitive delay,
    - **cerebral palsy**,
    - **severe visual impairment**,
    - **hearing impairment**

  (as defined below).

Death (CRF: ES, variable: REASON_EOS=2, 3)
Cognitive delay will be defined as an MDI<85 or other neurocognitive assessments including formal neurocognitive assessments subthreshold (e.g., defined as one standard deviation below the standardized mean) or ratings by family pediatricians (CRF: FU, variable: MDI or TEST_MDI_OTHER, defined and documented in dataset 'CPCS_CD' (variable: MDI_ADD=1) indicating that cognitive delay is present) by data management center CPCS Tübingen

Cerebral palsy: for the functional assessment the overall classification will be used (CRF: FU, variable: FU_KLASSIFIKATION=3) and type of CP is specified (CRF: FU, variable: FU_VORHANDEN=1,2,3,4).
Severe visual impairment will be defined as the best corrected vision in the better eye of visual acuity of 6m/60m or less (CRF: FU, variable: FU_BEURTEILUNG05=yes).
Severe hearing impairment is defined as a hearing loss requiring amplification or the insertion of a cochlear implant (CRF: FU, variable: FU_ BEURTEILUNG06=yes).

The primary outcome variable (binary) will be analyzed descriptively by treatment group. 95% confidence intervals for proportions will be calculated. A logistic regression with factors treatment, center and birth weight category (400-749g vs. 750-999g) will be applied to test the null hypothesis of equal proportions in the two treatment groups. ORs with 95% confidence interval and p-value will be presented.

10.2 Secondary and Other Endpoints

Key secondary endpoints at 24 months are:
- the individual **components of the composite primary outcome** described above (separately and in combination):
  - **death** (CRF: ES, variable: REASON_EOS=2, 3)
  - **cognitive delay** (MDI<85) (CRF: FU, variable: MDI) or subthreshold values from other neurocognitive assessments indicating cognitive delay (dataset: CPCS_CD, variable: MDI_ADD=1)
  - **cerebral palsy** (CRF: FU, variable: FU_KLASSIFIKATION=3 and FU_VORHANDEN= 1,2,3,4)
  - **severe visual impairment** (CRF: FU, variable: FU_BEURTEILUNG05=yes)
  - **severe hearing impairment** (CRF: FU, variable: FU_ BEURTEILUNG06=yes).
- the incidence of **cognitive delay** defined as MDI<70 (to match the more restrictive anglo-american definition) (CRF: FU, variable: MDI)
- **MDI** score (CRF: FU, variable: MDI), all values ≥50 were evaluable, imputed values (<50) will not be analyzed
- the physical developmental index (**PDI**) score (CRF: FU, variable: PDI),

Further secondary endpoints on short-term outcomes and long-term outcomes at 36 weeks postmenstrual age (CRF: RS) and follow-up assessment (CRF: FU) are:
- Score by **Gross motor function** classification system (variable: FU_GRAD)
- Measures of growth until follow-up assessment. Growth will be determined as
  - raw data: **length** (variable: LENGTH_RS_MOD, LENGTH_FU_MOD)
    weight (variable: WEIGHT_RS_MOD, WEIGHT_FU_MOD)
    head circumference (variable: HEAD_CIRCUMFENCE_RS_MOD, HEAD_CIRCUMFENCE_FU_MOD)

  - **SDS-Score** of length, weight, head circumference documented by data management center CPCS Tübingen (dataset: ‘CPCS_SDS’, variables: SDS-L-36, SDS-W-36, SDS-H-36, SDS-L-FU, SDS-W-FU, SDS-H-FU)
  - **SDS-Score-difference** from birth to 36 weeks PMA and to FU (where: difference from birth to 36 weeks PMA = SDS-W-36 – SDS-W-B, etc.)
- **length of hospital stay** (CRF: NOEH, variable: DATE_EOH) (final discharge home including the duration of care after referral to other centers)
- **time to last discontinuation** of
  - positive pressure respiratory support (CRF: NOEH, variable: EOH_ENDDATUM02)
  - respiratory stimulation with methylxanthines (CRF: NOEH, variable: EOH_ENDDATUM04)
  - gavage feeding (CRF: NOEH, variable: EOH_ENDDATUM05).

Other endpoints include all major diseases of prematurity:
- **BPD** (CRF: RS, variable: RS_FRAGE03, RS_FRAGE04, RS_FRAGE05)
  BPD=yes: RS_FRAGE03=yes
  or RS_FRAGE03=no and RS_FRAGE04=yes and RS_FRAGE05=yes
  BPD=no: RS_FRAGE03=no and RS_FRAGE04=no
  or RS_FRAGE03=no and RS_FRAGE04=yes and RS_FRAGE05=no
- Retinopathy of prematurity **ROP** (CRF: NOEH, variable: EOH_GRAD, EOH_GRAD02, EOH_BEHANDLUNG01, EOH_BEHANDLUNG02)
  - For ROP the maximum grade from *both eyes* is used:
    ROP=yes: EOH_GRAD>0 or EOH_GRAD02>0
    ROP=no: EOH_GRAD=0 and EOH_GRAD02=0
  - ROP severity group (maximum grade from *both eyes*): ROP mild (EOH_GRAD or EOH_GRAD02 =1,2) and severe (EOH_GRAD or EOH_GRAD02 = 3,4,5)
  - ROP therapy group (*both eyes*): EOH_BEHANDLUNG01>1 or EOH_BEHAND LUNG02>1
  - ROPsidedlight: ROP non eye (EOH_GRAD=0 *and* EOH_GRAD02=0)
    ROP one side (only EOH_GRAD>0 or EOH_GRAD02>0)
    ROP both eyes (EOH_GRAD>0 *and* EOH_GRAD02>0)
  - ROPsidedmaximal: ROP non eye (EOH_GRAD<3 *and* EOH_GRAD02<3)
    ROP one side (only EOH_GRAD≥3 or EOH_GRAD02≥3)
    ROP both eyes (EOH_GRAD≥3 *and* EOH_GRAD02≥3)
- necrotizing enterocolitis ≥ IIa after randomisation (**NEC**) (CRF: NOEH, variable: EOH_ERKRANKUNG03)
  - age on NEC occurrence
- **Focal intestinal perforation** after randomisation (CRF: NOEH, variable: EOH_ERKRANKUNG02)
  - age on occurrence of focal intestinal perforation,
- **PDA requiring therapy** (CRF: NOEH, variable: EOH_FRAGE03, EOH_ANTWORT03)
- **Nosocomial infections** (CRF: NOEH, variable: EOH_ANZAHL>0 or EOH_ANZAHL02>0 or EOH_ANZAHL03>0 or EOH_ANZAHL04>0)

- **Brain injury on cranial ultrasound** (CRF: HUS, variable: HUS_UNNORMAL01)
  - maximum grade of IVH/PVHI (1-4) over time
  - first day of maximum grade
  - cystic PVL
  - first day of cystic PVL

Secondary and other endpoints will be analyzed descriptively by treatment group providing counts, percentages or categorical endpoints and mean, standard deviation, minimum, first quartile, median, third quartile, maximum for quantitative endpoints. 95% confidence intervals for proportions will be calculated.
Binary endpoints and categorical endpoints with more than 2 values will be compared between treatment groups by logistic regression with factors treatment, center, and birth weight category (400-749g vs. 750-999g). ORs with 95% confidence interval and p-value will be presented.
Quantitative endpoints will be compared between treatment group by ANOVA with factors treatment, center and birth weight category (400-749g vs. 750-999g). Differences between means with 95% confidence interval and p-value will be presented.

10.3 Other post baseline Assessments

The following other post-baseline assessments will be collected during treatment, at the end of hospitalization, at defined points of time, at the end of study and at 24 month corrected age.

Neonatal outcome at EoH: (CRF: NOEH)
- Reason for end of hospitalization (variable: REASON_EOH)
- Days until transfer (variable: DATE_TRANSFER),
- Any air leaks requiring pleural drainage (variable: EOH_FRAGE01)
- Any pulmonary hemorrhages requiring transfusion (variable: EOH_FRAGE02)
- Diagnosis of PDA (variable: EOH_FRAGE03)
  if yes, Treated with Indomethacin (variable: EOH_ANTWORT01),
  Treated with Ibuprofen (variable: EOH_ANTWORT02),
  Surgical ligation performed (variable: EOH_ANTWORT03),
- Number of infants with at least one episode of blood or CSF culture-proven sepsis (variable: EOH_ANZAHL, 0=no, 1–>5=yes)
  Number of episodes of blood or CSF culture-proven sepsis (variable: EOH_ANZAHL, 0–>5)
- Number of infants with at least one episode of blood culture-proven sepsis with coagulase negative staphylococci (variable: EOH_ANZAHL02, 0=no, 1–>5=yes)
  Number of episodes of blood culture-proven sepsis with coagulase negative staphylococci (variable: EOH_ANZAHL02, 0–>5)
- Number of infants with at least one episode of presumed, but not blood culture-proven sepsis (variable: EOH_ANZAHL03, 0=no, 1–>5=yes)

Number of episodes of presumed, but not blood culture-proven sepsis (variable: EOH_ANZAHL03, 0–>5)

- Number of infants with at least one episode of pneumonia (variable: EOH_ANZAHL04, 0=no, 1–>5=yes)

  Number of episodes of pneumonia (variable: EOH_ANZAHL04, 0–>5)
- Any blood transmitted infection (variable: EOH_ERKRANKUNG)
- Focal intestinal perforation group: EOH_ERKRANKUNG02 0 vs 1 vs 2,3,
  EOH_ERKRANKUNG02 0 vs 1,2,3
- NEC group: EOH_ERKRANKUNG03 0 vs 1 vs 2,3
  EOH_ERKRANKUNG03 0 vs 1,2,3
- Number of ROP examinations (variable: EOH_ANZAHL05, 0–>5)
- Maximum grade of ROP at right eye (variable: EOH_GRAD)
- Plus disease at right eye (variable: EOH_ERKRANKUNG04)
- Mode of therapy at right eye (variable: EOH_BEHANDLUNG01)
- Maximum grade of ROP at left eye (variable: EOH_GRAD02)
- Plus disease at left eye (variable: EOH_ERKRANKUNG05)
- Mode of therapy at left eye (variable: EOH_BEHANDLUNG02)
- Respiratory supports:

  Is the discontinuation date (variable: EOH_ENDEDATUM01, EOH_ENDEDATUM02, EOH_ENDEDATUM03, EOH_ENDEDATUM04, EOH_ENDEDATUM05) = DATE_BIRTH (duration=0 days) then the infant did never get the support.

  If discontinuation date (variable: EOH_ENDEDATUM01, EOH_ENDEDATUM02, EOH_ENDEDATUM03, EOH_ENDEDATUM04, EOH_ENDEDATUM05) < DATE_EOH the patient got the support during hospital stay (DURATION_01_GR, DURATION_02_GR, DURATION_03_GR, DURATION_04_GR, DURATION_05_GR = inside).

  If the discontinuation date (variable: EOH_ENDEDATUM01, EOH_ENDEDATUM02, EOH_ENDEDATUM03, EOH_ENDEDATUM04, EOH_ENDEDATUM05) ≥ DATE_EOH then the infant continued the support at home (DURATION_01_GR, DURATION_02_GR, DURATION_03_GR, DURATION_04_GR, DURATION_05_GR = outside).
    - DURATION_01_GR
    - Days until mechanical ventilation via ET-tube was finally discontinued for DURATION_01_GR=inside (variable: EOH_ENDEDATUM01)
    - DURATION_02_GR
    - Days until positive airway pressure (CPAP) was finally discontinued for DURATION_02_GR=inside (variable: EOH_ENDEDATUM02)
    - DURATION_03_GR
    - Days until supplemental oxygen was finally discontinued for DURATION_03_GR=inside (variable: EOH_ENDEDATUM03)
    - DURATION_04_GR
    - Days until methylxanthine administration was finally discontinued for DURATION_04_GR=inside (variable: EOH_ENDEDATUM04)
    - DURATION_05_GR

- Days until tube / gavage feedings were finally discontinued for DURATION_05_GR=inside (variable: EOH_ ENDEDATUM05)

- Doxapram administered at any time for apnea of prematurity (variable: EOH_ BEHANDLUNG03)
- Any re-intubation primarily for severe idiopathic apnea of prematurity (variable: EOH_BEHANDLUNG04)
- Transfusion guidelines applied until discharge (variable: EOH_BEHANDLUNG05)
  if others, days until drop-out / premature withdrawal from assigned guideline (variable: DATE_DROPOUT) including listing of these patients with reasons (variable: TXT_DROPOUT)

HUS at Day 0, Day 7, day 28, week 36 PMA: (CRF: XHU)
- Postnatal age (in days after birth) until head ultrasound
- Normal result of HUS (variable: RESULT_HUS) (statistical test) at each time point and overall
  if not normal,
  - Maximum grade of intraventricular / periventricular hemorrhage (variable: HUS_UNNORMAL01)
  - Flare (variable: HUS_UNNORMAL02)
  - Cystic periventricular leukomalacia (PVL) (variable: HUS_UNNORMAL03)
  - Porencephalic cyst (variable: HUS_UNNORMAL04)
  - Ventriculomegaly (variable: HUS_UNNORMAL05)
  - Cortical atrophy (variable: HUS_UNNORMAL06)
  - Other result (variable: HUS_UNNORMAL07)
- HUS result group: HUS_UNNORMAL01=3,4 or HUS_UNNORMAL03=1,2 or HUS_UNNORMAL04=yes, at each time point and overall

Respiratory Status at 36 weeks PMA: (CRF: RS)
- Postmenstrual age (in weeks) at assessment of respiratory status
- Any postnatal dexamethasone for BPD therapy (variable: RS_FRAGE01)
- Any postnatal inhaled steroids for BPD therapy (variable: RS_FRAGE02)
- Does the baby receive any of the listed supports (variable: RS_FRAGE03),
  if no, Does the baby receive any supplemental oxygen (variable: RS_FRAGE04),
  if yes, oxygen saturation outside given ranges (variable: RS_FRAGE05),
- BPD, definition see chapter secondary endpoints
  RS_FRAGE03, RS_FRAGE04, RS_FRAGE05 in combination

Follow-Up assessment at 24 months +/- 1 month corrected age: (CRF: FU)
- Corrected age (in months after PMA40weeks) at anthropometric measures at follow-up (variable: DATE_ANTHRO)
  time until measure (<20 months, 20-28 months, >28 months), including listing of children with time until measure <20 months or >28 months
- Report of anthropometric measure (variable: REPORT_ANTHRO)
- Corrected age (in months after PMA40weeks) at final Bayley assessment at follow-up (variable: DATE_BAYLEY)

time until Bayley assessment (<20 months, 20-28 months, >28 months), including listing of children with time until Bayley assessment <20 months or >28 months

- Report of Bayley test (variable: REPORT_BAYLEY)
- Corrected age (in months after PMA40weeks) at neurological examination (variable: DATE_NEURO)
  time until examination (<20 months, 20-28 months, >28 months), including listing of children with time until examination <20 months or >28 months
- Report of neurological examination (variable: REPORT_NEURO)
- Overall classification (variable: FU_KLASSIFIKATION)
  if cerebral palsy, type of CP (variable: FU_VORHANDEN), including listing of patients with FU_KLASSIFIKATION=3 and FU_VORHANDEN=0
- Grasp / grip with right hand (variable: FU_BEURTEILUNG01)
- Grasp / grip with left hand (variable: FU_BEURTEILUNG02)
- Stand, walk, run (variable: FU_BEURTEILUNG03)
- Speech (variable: FU_BEURTEILUNG04)
- Family situation at time of follow-up (variable: FAMILY_SITUATION)
- Caregiver education at time of follow-up (variable: EDUCATION)
- Caregiver occupation at time of follow-up (variable: OCCUPATION)
- Number of re-admissions to hospital since discharge (variable: READMISSIONS)
- CNS seizures since discharge (variable: CNS_SEIZURES)

The above other post-baseline assessments will be analyzed descriptively by treatment group providing counts, percentages or categorical endpoints and mean, standard deviation, minimum, first quartile, median, third quartile, maximum for quantitative endpoints. 95% confidence intervals for proportions will be calculated.
Statistical tests will be applied as assigned in the above.
Binary endpoints, as Normal results of HUS, will be compared between treatment groups by logistic regression with factors treatment, center, and birth weight category (400-749g vs. 750-999g). ORs with 95% confidence interval and p-value will be presented.

Overall Survival
Overall survival is defined as time interval from birth (CRF: BA; variable: DATE_BIRTH) until death (event, CRF: ES; variable: DATE_DEATH) or last observation (censored, CRF: FU; variable: last date of DATE_ANTHRO, DATE_BAYLEY, DATE_NEURO).
Overall survival will be analyzed according to the method of Kaplan-Meier by treatment group.

10.4 Subgroup Analysis

The following subgroups will be analyzed in the ITT-population:

(1) infants with birth weights 400-749g and 750-999g
(2) male and female infants
(3) infants from centers with ‘higher’ and ‘lower’ oxygen saturation targets (where centers with ‘lower’ oxygen saturation targets are defined by a central value of the target oxygen saturation range of less than the median of the central values of the target oxygen saturation ranges of all centers (dataset: ‘CPCS_SPO2’).

Only the primary endpoint, the incidence of cerebral palsy, and the MDI-Score will be analyzed in these subgroups by logistic regression or by ANOVA with factor treatment, and if applicable center and birth weight category (400-749g vs. 750-999g).

11. Safety Analyses

Adverse Events (AE)

AE data will be analyzed descriptively. The following analyses will be provided:

a) AE overview with counts and percentages by treatment group for the following categories:
   - AE
   - Serious Adverse Event (SAE)
   - AE leading death
   - AE by relationship
   - AE by severity
   - AE by SOC/PT

b) Listing of SAEs, AEs leading to death
c) AE overview according to a) with counts and percentages by treatment groups will be provided for the following subgroups: weight category (400-749g vs. 750-999g), gender

12. List of Data provided by the Data Management in addition to ETTNO database read-out

Data-Set-Title	Variables indicated (in addition to patient screening number)	Meaning
CPCS_CD	MDI_ADD	1= cognitive delay present<br>0= no cognitive delay
CPCS_BW	CAUSE1	1 = birth weight >999g<br>0 = unknown
CPCS_EM	JUSTIFIED	1 = text for other emergency reason is justified<br>0= text for other emergency reason is *not* justified
CPCS_SPO2	SPO2-Target<br><br><br><br><br><br>SPO2_DATE	1=high (center value>90%)<br>2=low (center value≤90%)<br>3=changed during study period from low to high<br>4= changed during study period from high to low<br>Date of change
CPCS_SDS	SDS-W-B<br>SDS-L-B<br>SDS-H-B<br>SDS-W-36<br>SDS-L-36<br>SDS-H-36<br>SDS-W-FU<br>SDS-L-FU<br>SDS-H-FU	at birth:<br><br><br>at 36 weeks PMA:<br><br><br>at Follow-up:
CPCS_RD	ReasonDeath	Pulmonary Complications (=1)<br>Cardiac complication (=2)<br>Gastrointestinal complication (=3)<br>Sepsis/Infection (=4)<br>IVH/PVL (=5)<br>Renal complication (=6)<br>Multiorgan failure (=7)<br>Prematurity/ELBW (=8)<br>Other reasons for death (=9)
